# Human iPSC-derived cerebral organoids model features of Leigh syndrome and reveal abnormal corticogenesis

**DOI:** 10.1242/dev.199914

**Published:** 2022-07-06

**Authors:** Alejandra I. Romero-Morales, Gabriella L. Robertson, Anuj Rastogi, Megan L. Rasmussen, Hoor Temuri, Gregory Scott McElroy, Ram Prosad Chakrabarty, Lawrence Hsu, Paula M. Almonacid, Bryan A. Millis, Navdeep S. Chandel, Jean-Philippe Cartailler, Vivian Gama

**Affiliations:** 1 Department of Cell and Developmental Biology, Vanderbilt University, Nashville, TN 37232, USA; 2 Feinberg School of Medicine, Department of Medicine, Division of Pulmonary and Critical Care Medicine, Northwestern University, Chicago, IL 60611, USA; 3 Creative Data Solutions, Vanderbilt Center for Stem Cell Biology, Vanderbilt University, Nashville, TN 37232, USA; 4 School of Economics and Finances, Universidad EAFIT, Colombia; 5 Vanderbilt Biophotonics Center, Vanderbilt University, Nashville, TN 37232, USA; 6 Feinberg School of Medicine, Department of Biochemistry and Molecular Genetics, Northwestern University, Chicago, IL 60611, USA; 7 Vanderbilt Brain Institute, Vanderbilt University, Nashville, TN 37232, USA

**Keywords:** Leigh syndrome, Stem cells, Glycolysis, Oxidative phosphorylation, Mitochondria, Neural precursor cells, Neural rosettes, Brain organoids

## Abstract

Leigh syndrome (LS) is a rare, inherited neurometabolic disorder that presents with bilateral brain lesions caused by defects in the mitochondrial respiratory chain and associated nuclear-encoded proteins. We generated human induced pluripotent stem cells (iPSCs) from three LS patient-derived fibroblast lines. Using whole-exome and mitochondrial sequencing, we identified unreported mutations in pyruvate dehydrogenase (GM0372, PDH; GM13411, MT-ATP6/PDH) and dihydrolipoyl dehydrogenase (GM01503, DLD). These LS patient-derived iPSC lines were viable and capable of differentiating into progenitor populations, but we identified several abnormalities in three-dimensional differentiation models of brain development. LS patient-derived cerebral organoids showed defects in neural epithelial bud generation, size and cortical architecture at 100 days. The double mutant MT-ATP6/PDH line produced organoid neural precursor cells with abnormal mitochondrial morphology, characterized by fragmentation and disorganization, and showed an increased generation of astrocytes. These studies aim to provide a comprehensive phenotypic characterization of available patient-derived cell lines that can be used to study Leigh syndrome.

## Abstract (precis)

**Summary:** Induced pluripotent stem cell-derived model systems reveal defects in early neurogenesis due to dysregulation of key metabolic pathways associated with Leigh syndrome.

## INTRODUCTION

Leigh syndrome (LS), or sub-acute necrotizing encephalomyelopathy, is an inherited neurometabolic disorder that affects the central nervous system (CNS) (Baertling et al., 2014; Gerards et al., 2016; Leigh, 1951; Sorbi and Blass, 1982). LS is a rare, progressive, early-onset disease with a prevalence of 1 in 40,000 live births (Lake et al., 2016). The pathologic features of LS are focal, bilateral lesions in one or more areas of the CNS, including the brainstem, thalamus, basal ganglia, cerebellum, cortex and spinal cord (Alves et al., 2020; Sofou et al., 2018). The most common underlying cause is defective oxidative phosphorylation (OXPHOS) due to mutations in genes encoding complexes of the mitochondrial respiratory chain (Baertling et al., 2014; Lake et al., 2015, 2016).

The availability of animal models (Ferrari et al., 2017; Jain et al., 2016, 2019) and brain tissue from biopsies has provided crucial insight into this disease. However, our understanding of the etiology and pathology of complex neurological diseases like LS would benefit from human-derived platforms such as induced pluripotent stem cell (iPSC)-derived models (Quadrato et al., 2016). The ability to reprogram somatic cells into iPSCs, followed by differentiation into specific lineages, has become a useful tool for complex disease modeling (Di Lullo and Kriegstein, 2017; Kelava and Lancaster, 2016; Paşca, 2018; Quadrato et al., 2016). In the context of LS, iPSCs have been successfully generated from patients with mutations in mitochondrially encoded ATP synthase membrane subunit 6 (*MT-ATP6*) (Galera-Monge et al., 2016; Grace et al., 2019; Lorenz et al., 2017; Ma et al., 2015), mitochondrially encoded NADH:ubiquinone oxidoreductase core subunit 3 (*MT-ND3*) subunit (Hattori et al., 2016) and the nuclear-encoded gene surfeit locus protein 1 (*SURF1*) (Inak et al., 2021). These iPSC-model systems have been proposed for drug discovery (Inak et al., 2017; Lorenz et al., 2017) as well as testing platforms for potential metabolic rescue treatments (Ma et al., 2015).

Many studies have used LS patient fibroblasts commercially available at the Coriell Institute (Galera-Monge et al., 2016; Hinman et al., 1989; Huh et al., 1990; Iyer et al., 2012; Johnson et al., 2019; Ma et al., 2015; Sorbi and Blass, 1982; Vo et al., 2007; Zheng et al., 2016a). Here, we report our findings on the genomic and phenotypic characterization of iPSCs generated from these LS patient-derived fibroblast lines. Whole-exome sequencing (WES) and mitochondrial sequencing revealed previously unidentified mutations in these patient-derived cell lines. Three-dimensional differentiation of LS patient-derived iPSCs into neural rosettes (NRs) and cerebral organoids resulted in severe abnormalities. LS patient-derived cerebral organoids grown for 100 days showed defects in the generation of neural epithelial buds and impaired corticogenesis. These results indicate that aberrant corticogenesis may drive LS pathogenesis and demonstrate the utility of iPSC-derived systems to recapitulate CNS phenotypes and test potential strategies to restore neurogenesis in LS.

## RESULTS

### Genomic characterization of LS fibroblasts

**Fig. 1.**
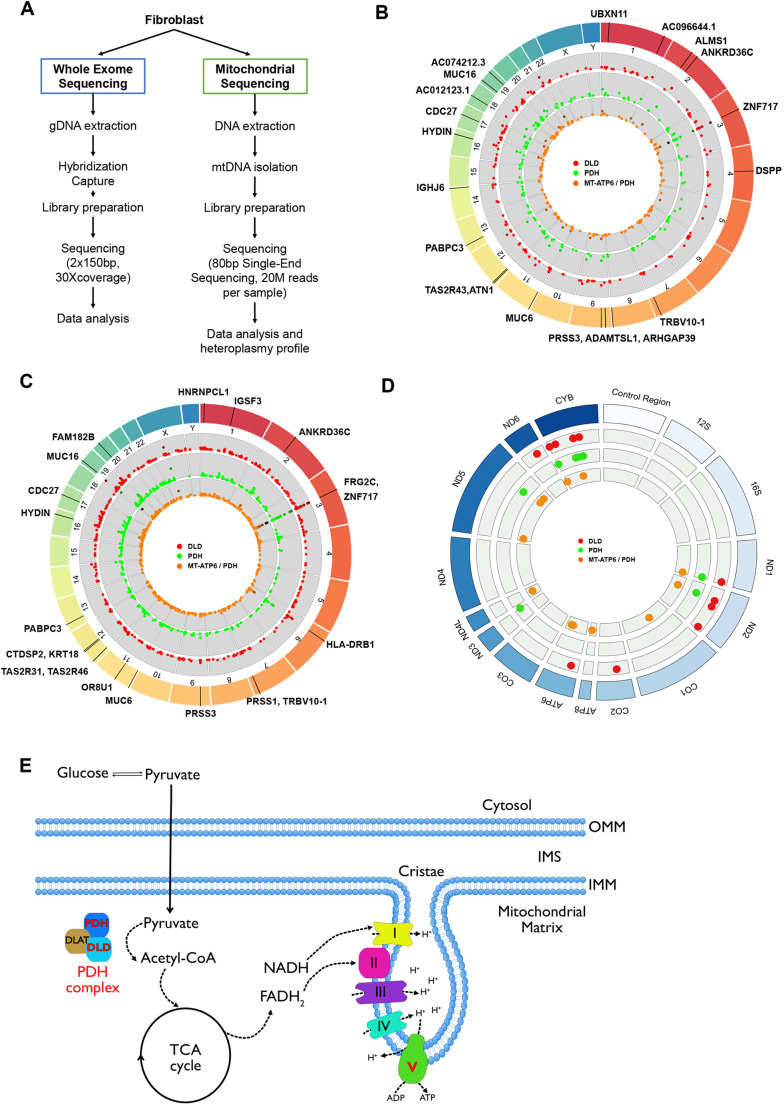
**Whole-exome sequencing identifies novel mutations in LS fibroblasts.** (A) Schematic of the WES and mitochondrial sequencing workflow. (B,C) Representation of WES data, highlighting the top 20 genes containing high impact indels (B) and top 20 genes containing high impact SNPs (C; increased likelihood of disrupting protein function). (D) Mitochondrial sequencing identifies novel mutations in LS fibroblasts. Representation of mitochondrial sequencing data, highlighting mitochondrial genes containing mutations (transitions, deletions or transversions). Red dots, DLD line; green dots, PDH line; orange dots, MT-ATP6/PDH line. (E) Representation of the affected proteins in the LS cell lines. PDH and DLD are part of the pyruvate dehydrogenase complex (PDHc). MT-ATP6 is a subunit of the ATP synthase, represented here as the electro transport chain complex V. DLD, dihydrolipoyl dehydrogenase; IMM, inner mitochondrial membrane; IMS, inner mitochondrial space; MT-ATP6/PDH, mitochondrially encoded ATP synthase membrane subunit 6/pyruvate dehydrogenase; OMM, outer mitochondrial membrane; PDH, pyruvate dehydrogenase.

Due to the limited genomic information available for the three cell lines (Table S1), we performed WES and mitochondrial sequencing of the fibroblasts before reprogramming (Fig. 1A-D; Fig. S1; data repository can be found at https://www.ncbi.nlm.nih.gov/sra/PRJNA626388 and https://vandydata.github.io/Romero-Morales-Gama-Leigh-Syndrome-WES/). Comparison between the high impact, moderate impact and all variants for identified insertion/deletions (INDELs) and single-nucleotide polymorphisms (SNPs) showed significant overlap between the three cell lines (Fig. S1A,B). In-depth analysis of the top 15 high-impact SNPs (Fig. S1C) also confirmed an overlap between genotypes, with only three genes with confirmed SNPs related to neurological diseases (*FRG2C* for bipolar disorder, and *CDC27* and *KIR2DL4* for white matter microstructure measurements) (Buniello et al., 2019).

Targeted analysis of the genes associated with LS (Lake et al., 2016) revealed a loss-of-function INDEL frameshift in pyruvate dehydrogenase complex (PDHc) E1 alpha 1 subunit or pyruvate dehydrogenase (*PDHA1*, c.79delC, p.Arg27fs) in the lines GM03672 and GM13411 (Fig. 1E). An SNP in the PDHc E3 subunit or dihydrolipoyl dehydrogenase (*DLD*, c.100A>G, p.Thr34Ala) was identified in GM01503 (Fig. 1E). In addition to being part of PDHc, DLD is also a component of the α-ketoglutarate and branched-chain α-ketoacid dehydrogenase complexes (Craigen, 1996). Despite the lack of genomic data, dysfunction of PDHc has been previously suggested as the main driver of the disease in these patients (Hinman et al., 1989; Huh et al., 1990; Sorbi and Blass, 1982) (Table S1). To our knowledge, mutations in the nuclear genome of GM13411 have not been reported.

Mitochondrial sequencing identified several SNPs in all the lines (Fig. 1D). A loss-of-function SNP in the *MT-ATP6* gene was identified in the GM13411 line. This mutation was reported in the original clinical case (Pastores et al., 1994). The authors described the T-to-G mutation at position 8993 that results in the substitution of a highly conserved leucine residue for an arginine (L156R). MT-ATP6 is part of the F0 domain of ATP synthase, which functions as a proton channel (Fig. 1E). The L156R substitution prevents the induction of c-ring rotation of ATP synthase (Kühlbrandt and Davies, 2016), resulting in decreased ATP synthesis (Uittenbogaard et al., 2018). Heteroplasmy analysis of fibroblasts showed a 92% frequency of this mutation in the cell population, consistent with previous reports (Galera-Monge et al., 2016; Iyer et al., 2012; Pastores et al., 1994).

### Characterization of iPSCs derived from commercially available LS fibroblasts

Reprogramming of fibroblasts was performed as previously described (Takahashi et al., 2007) (Fig. S2A). Pluripotency was evaluated using the microarray-based analysis PluriTest (Müller et al., 2011). All three LS cell lines showed a high pluripotency score and a low novelty score (Fig. S2B,C), congruent with the transcriptional profile of pluripotent stem cells. Moreover, all the reprogrammed cells expressed the pluripotency markers *NANOG* and *POU5F1* (*OCT4*) (Fig. S2D). The MT-ATP6/PDH cell line showed increased levels of NANOG (*P*<0.0001) compared with control.

To assess the ability of the LS and control cell lines to differentiate into the three germ layers, we performed trilineage differentiation as previously described (Kuang et al., 2019; Roberts et al., 2019) and measured expression of several genes using real time quantitative PCR (RT-qPCR). Commitment to ectodermal fate was evaluated by expression of the genes *GATA3* and *PAX6*, endoderm fate was evaluated by the expression of the genes *CDX2* and *SOX17*, and mesodermal fate was evaluated by the expression of the genes *TBXT* and *NCAM* (*NCAM1*) (Fig. S2E). Although all the mutant cell lines can generate cells positive for the three germ layer markers without statistical differences, we observed an inherent variability in the differentiation efficiency among clones that may be due to differences in the genetic backgrounds (heteroplasmy or potential X-linked gene silencing) (Juchniewicz et al., 2021; Lissens et al., 2000; Migeon, 2020).

### Two-dimensional neural differentiation is not significantly altered by LS-associated mutations

**Fig. 2.**
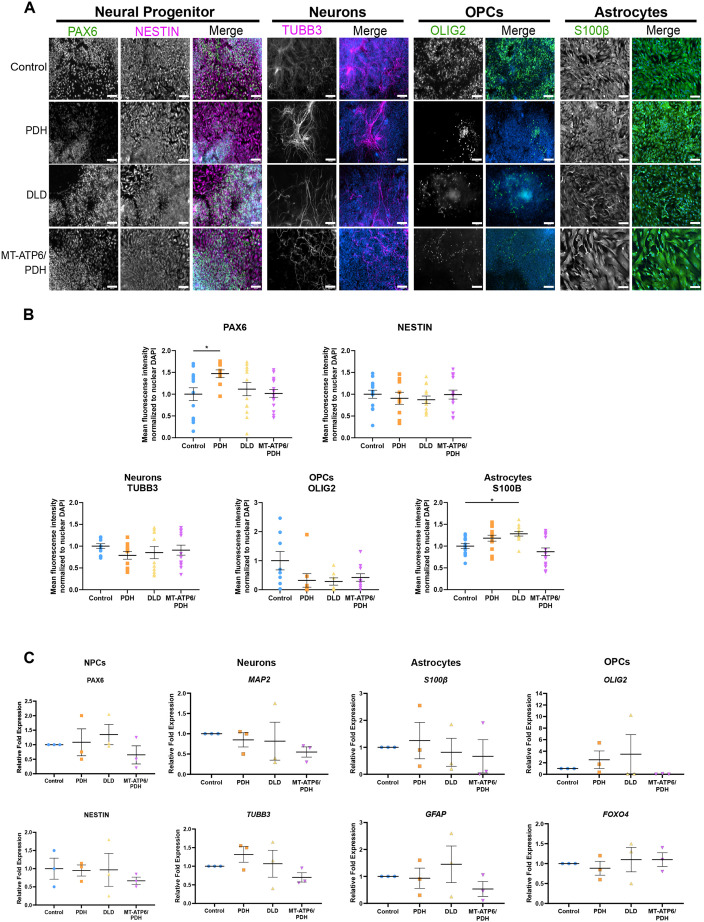
**Leigh syndrome-derived NPCs are multipotent.** (A) Representative images of the expression of neural multipotency markers. NPCs stained by PAX6 and NES (NESTIN), neurons marked with βIII-tubulin (TUBB3), oligodendrocyte progenitor cells (OPCs) stained with Olig2, and astrocytes marked with S100β. Merged panels show the color image of the grayscale lineage marker and the nuclear staining DAPI in blue. Scale bars: 100 μm. (B) Immunofluorescence quantification. A slight increase in the number of PAX6+ cells was observed in PDH (*P=0.494; one-way ANOVA). S100β+ cells were also increased in the DLD mutant line (*P=0.0185; one-way ANOVA). Three independent differentiations were performed. Positive nuclei number for nuclear markers, and mean fluorescence intensity for cytoplasmic markers, were normalized to the nuclear DAPI intensity/number and the intensity values of control. (C) RT-qPCR analysis of the NPC markers *PAX6* and *NES*, as well as the multipotency markers *MAP2* and *TUBB3* for neuronal lineage, *S100B* and *GFAP* for astrocytic lineage, and *OLIG2* and *FOXO4* for OPCs. Fold change normalized to GPI and GAPDH as housekeeping genes. Graphs show mean±s.e.m.

To determine whether the LS mutations impact the commitment and development of the neural lineage, neural precursor cells (NPCs) (a mixed population of neural stem and progenitor cells) were generated by a dual SMAD inhibition protocol (Chambers et al., 2009) (Fig. S3A). NPCs expressed expected neural markers: PAX6, nestin (NES) and SOX2 (Fig. 2A; Fig. S3B). A slight increase was observed in PAX6+ nuclei in the PDH mutant (*P*=0.494, Fig. 2B), but no other differences were identified (Fig. 2B; Fig. S3B). The multipotent capacity of NPCs to generate neurons, astrocytes and oligodendrocytes was evaluated using immunostaining and RT-qPCR (Fig. 2A-C). We identified an increase in the mean fluorescence intensity of the astrocyte marker S100β in the DLD mutant line (*P*=0.0185), suggesting a propensity of these cells to commit to the astrocyte lineage.

Neural cell death is a hallmark of LS; thus, we performed a cell viability assay to investigate the sensitivity of the LS patient-derived NPCs to different apoptotic stimuli (Fig. S3C). Treatment with DNA damaging agents, etoposide and neocarzinostatin, and the microtubule depolymerizing agent nocodazole, did not show increased sensitivity to cell death. Treatment with CCCP, a mitochondrial uncoupler, did not show increased susceptibility of the NPCs to mitochondrial damage. Thus, LS-causing mutations do not affect the sensitivity of the NPCs to apoptotic stimuli.

To investigate the metabolic effects of LS-causing mutations at the iPSC and NPC states, we performed metabolic analyses using the Seahorse Mito Stress Test. This assay provides a readout of bioenergetic function by assessing several parameters including oxygen consumption rate (OCR) and extra cellular acidification rate (ECAR). Previous studies show that iPSCs mainly rely on glycolysis to generate ATP and intermediates that contribute to pluripotency and self-renewal (Chandel et al., 2016; Folmes et al., 2011; Hamanaka and Chandel, 2010; Kondoh et al., 2007). The low levels of OXPHOS have been attributed, at least in part, to an immature and fragmented mitochondrial network (Cho et al., 2006; Chung et al., 2010; Folmes et al., 2011; Prigione et al., 2010; Zhang et al., 2011). Although LS patient-derived iPSCs do not show significant differences in OCR (Fig. S4A), ECAR (proxy of glycolysis) was reduced in the MT-ATP6/PDH mutants compared with control (Fig. S4B). Analysis of other bioenergetic parameters in these cells also showed dysregulation in the non-mitochondrial OCR (*P*=0.0284, Fig. S4C), which has been associated with highly proliferative cells (Herst and Berridge, 2007; Krisher and Prather, 2012; Manes and Lai, 1995; Muller et al., 2019; Starkov, 2008).

Differentiated cells have more complex mitochondrial networks and use OXPHOS as the main source of ATP (Mandal et al., 2011; Suhr et al., 2010; Wu et al., 2016; Yanes et al., 2010). The metabolic switch from glycolysis to OXPHOS is a hallmark of NPC differentiation (Agathocleous et al., 2012; Zheng et al., 2016b). Although all lines showed similar levels of glycolysis, only PDH and DLD NPCs showed similar levels of OXPHOS compared with control (Fig. S4D,E). OCR values in MT-ATP6/PDH mutant cells were significantly lower after FCCP treatment (Fig. S4D), which translates into a reduced spare respiratory capacity (Fig. S4F, *P*=0.0354), reflecting lower metabolic fitness and a deficiency in engaging the metabolic switch during differentiation. Non-mitochondrial oxygen consumption was also lower in MT-ATP6/PDH NPCs (*P*=0.0317).

### LS mutations cause morphological alterations in three-dimensional models of neurodevelopment

Previous studies using cells from LS patients carrying homozygous *SURF1* (c.769G>A and c.530T>G) and *MT-ATP6* (m.9185T>C) mutations showed an abnormal generation of neural lineages (Lorenz et al., 2017) and impaired neurogenesis in cerebral organoids (Inak et al., 2021). Therefore, we investigated the effects of the PDH, DLD and MT-ATP6/PDH mutations on neurogenesis using three-dimensional models of neural development (Lancaster and Knoblich, 2014; Romero-Morales et al., 2019 preprint).

**Fig. 3.**
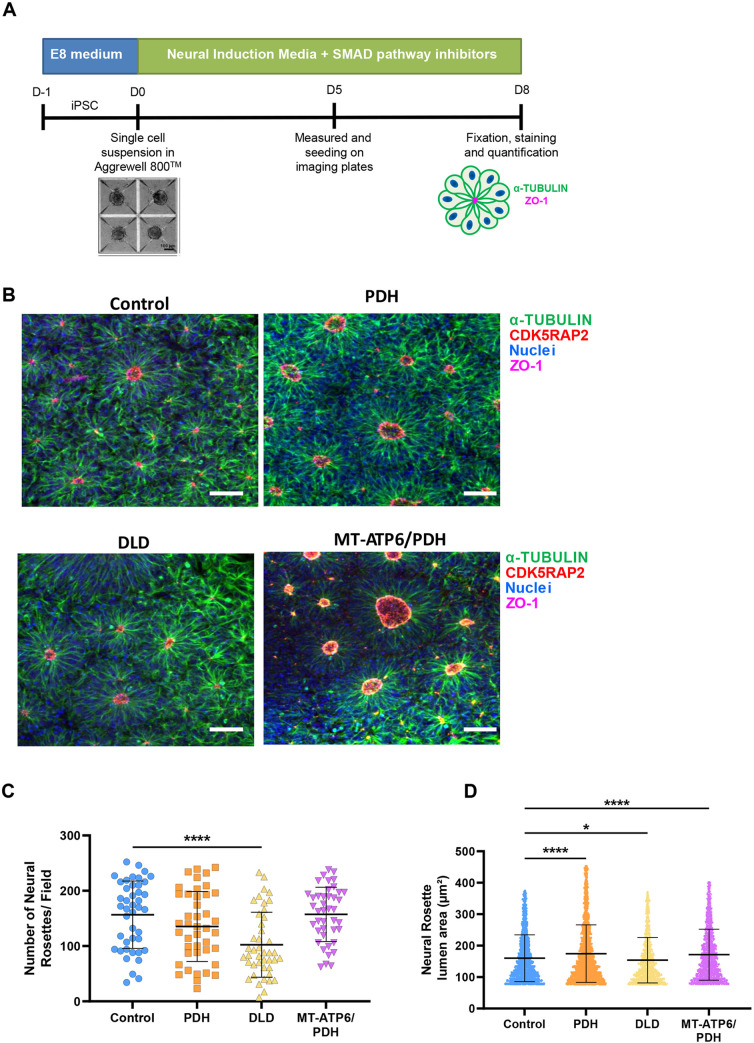
**Three-dimensional differentiation reveals abnormalities during induction of neural rosettes in LS cell lines.** (A) Schematic of neural rosette (NR) generation protocol. (B-D) Representative confocal images of NRs (B) show decreased numbers of NRs per field in the DLD mutant line (C). Quantification of the lumen area (μm^2^; D) indicates increased lumen area in the PDH and MT-ATP6/PDH mutant cell lines and a decreased lumen area in the DLD mutant line. Scale bars: 50 μm. *P<0.05; ****P<0.0001. Quantification was performed with images acquired using a 10× objective. Representative figures were acquired at 20× magnification to better appreciate the differences in morphology. Graphs show mean±s.e.m.

To examine the effects of LS-associated mutations in the early stages of CNS development, we generated NR using embryoid bodies (EBs) grown in the presence of SMAD inhibitor media (Fig. 3A) (Elkabetz et al., 2008; Zhang et al., 2001). These structures have previously been shown to recapitulate the early neural tube formation stage of development (Elkabetz et al., 2008; Wilson and Stice, 2006). NRs were stained for the tight junction marker ZO-1 (Elkabetz et al., 2008; Hříbková et al., 2018) and the centrosomal marker CDK5RAP2 (Fig. 3B). Quantification of the number of NRs per field of view showed fewer of these structures in the DLD mutant (Fig. 3C, *P*<0.001). Lumen area quantification revealed that PDH and MT-ATP6/PDH mutants have larger lumen areas, whereas the DLD mutant line showed a smaller area relative to controls (Fig. 3D; PDH: *P*<0.0001, DLD: *P*=0.0236 and MT-ATP6/PDH: *P*<0.0001). The NRs obtained from all cell lines followed the expected morphological changes described previously (Hříbková et al., 2018). The polymerization of α-tubulin and generation of the ZO-1 ring at the apical region of the rosettes are conserved in the LS mutants. Increased NR lumen size has previously been associated with activation of the TGFβ pathway (Medelnik et al., 2018), Notch and sonic hedgehog (SHH) pathway, and inhibition of WNT (Elkabetz et al., 2008). Large rosette formation is thought to be a consequence of coalescence or fusion of smaller rosettes (Fedorova et al., 2019) or apical domain opening and expansion (Medelnik et al., 2018) rather than a process dependent on cell proliferation.

### LS-associated mutations disrupt corticogenesis in cerebral organoids

Cortical abnormalities in LS have been associated with developmental delay and disease progression. Imaging studies have shown an incidence of cortical lesions in over 20% of the patients, with this finding being highly associated with mitochondrial DNA (mtDNA) etiology (Alves et al., 2020). To investigate the effects of these mutations during corticogenesis, we generated cerebral organoids from LS patient-derived iPSCs (Fig. S5A).

Differences between the cell lines became apparent as early as the neuroepithelial bud expansion phase. After Matrigel embedding, the MT-ATP6/PDH mutant cell line showed poor budding with large areas of non-neuroepithelial cells (Fig. S5B). Defective organoid formation in this cell line was significantly higher than in the control and the other two LS cell lines (Fig. S5C). A previous report showed that iPSCs generated from fibroblasts harboring the same T8993G mtDNA mutation failed to form EBs. There was rapid regression and death after 7 days in suspension, whereas the monolayer culture did not show obvious deficits in cell growth (Grace et al., 2019). Given that the neuroectoderm expansion phase happens during days 7-10, the degeneration of the MT-ATP6/PDH organoids after embedding is consistent with these reports. Higher metabolic requirements are associated with NPC proliferation and migration in three-dimensional scaffolds and development than in monolayer cultures (Fang et al., 2020; Homem et al., 2015). As the PDH mutant line did not show this particular phenotype at this stage, the presence of the mitochondrial mutation in the MT-ATP6/PDH line may be responsible for the reduction in organoid formation efficiency.

Given that undirected/unpatterned brain organoid protocols can generate different regions of the CNS (Kanton et al., 2019; Quadrato et al., 2016; Sidhaye and Knoblich, 2021), we characterized day 15 organoids by comparing the expression of dorsal forebrain markers with the fetal brain (Fig. S5D). Dorsal forebrain markers *SOX2* and *TBR2* (*EOMES*) were expressed in the control organoids as expected. *SOX2* expression was increased in PDH (*P*=0.0026) and DLD (*P*=0.0029), whereas *TBR2* expression was lower in control (*P*=0.0479), PDH (*P*=0.0275), and DLD (*P*=0.0366) organoids when compared with the fetal brain control. Markers for the telencephalic ventral fate were expressed at very low levels in all genotypes (*P*<0.0001 in all cases). *GATA3*, an early marker for mesencephalic fate, was reduced in all three LS organoids (PDH: *P*=0.0096, DLD: *P*=0.0136, MT-ATP6/PDH: *P*=0.0019). *OTX2*, expressed in the diencephalon, mesencephalon and choroidal plexus (Larsen et al., 2010), was upregulated in the control (*P*=0.0295), PDH (*P*=0.0009) and DLD (*P*=0.0494) organoids when compared with the fetal brain RNA expression. Lastly, the diencephalon marker *GBX2* was expressed similarly among samples, whereas *PCP4* was lower in all genotypes (control: *P*=0.0010, PDH: *P*=0.0003, DLD: *P*=0.0048, MT-ATP6/PDH: *P*=0.0003). These data indicate that LS-associated mutations do not compromise the ability of cells at early stages to commit to a telencephalic fate.

To assess the effect of the LS mutations during the first stages of neural development, we collected mRNA of day 30 organoids and evaluated the expression of NPC and cortical markers by RT-qPCR (Fig. S6A). *SOX2*, an NPC marker, showed reduced expression in all three LS mutants (PDH: *P*=0.0156, DLD: *P*=0.0303, MT-ATP6/DPH: *P*<0.0001). The expression of NPC markers *NES* and *PAX6* was increased in PDH mutant organoids (*P*=0.0156 and *P*=0.0134, respectively). MT-ATP6/PDH organoids showed a reduction in the expression of *PAX6* (*P*=0.0231) and an increase in the expression of the intermediate progenitor cell (IPC) marker *TBR2* (*P*=0.0224). The cortical plate marker *CTIP2* (*BCL11B*) was found to be reduced in both DLD (*P*=0.0080) and MT-ATP6/PDH (*P*<0.0001); and the neuronal marker βIII-tubulin (*TUBB3*) was reduced in MT-ATP6/PDH (*P*=0.0302). No significant differences were noted in expression of the glycoprotein *RELN* or the cortical plate marker *TBR1* among the different genotypes.

The reduction in expression of NPC markers *SOX2* and *PAX6* in MT-ATP6/PDH mutant organoids with a concomitant increase in *TBR2* may suggest a premature commitment to IPCs (Englund, 2005; Hutton and Pevny, 2011; Sansom et al., 2009). This premature differentiation into IPCs and reduced expression of committed neuronal markers, such as *CTIP2* and *TUBB3*, may suggest an inability to acquire a neuronal fate in this genotype.

Brain organoids were sectioned and stained for ventricular zone (VZ), subventricular zone (sVZ) and cortical plate (CP) markers (Fig. S6B-E). Day 30 organoids were obtained from at least three independent batches of differentiation, and representative images were obtained from at least four individual organoids per batch. Quantification of immunofluorescence images revealed no significant differences in the number of NPCs positive for SOX2, PAX6 and NES or the IPC marker TBR2 (Fig. S6F). In agreement with the defective neuroepithelial expansion, the overall architecture in MT-ATP6/PDH organoids was compromised. Few ventricle-like structures were present, and the foci of PAX6+ cells were not organized in the expected radial pattern. Migration of early-born neurons *in vivo* depends on pioneer Cajal-Retzius neurons that are positive for the glycoprotein RELN (Lancaster et al., 2017). Cells positive for this marker were identified in superficial regions of all organoids. Early-born neurons positive for CTIP2 and TBR1 were observed in all genotypes. The neuronal marker microtubule-associated protein 2 (MAP2) was also present in all samples at similar levels to control (Fig. S6E,F). Expression of the outer radial glia (oRG) marker homeodomain-only protein (HOPX) was significantly reduced in the PDH (*P*<0.0001) and MT-ATP6/PDH mutants (*P*=0.0417). Metabolic stress has been correlated with reduced specification in organoids, especially in oRG and newborn neurons (Bhaduri et al., 2020). Hence, lower levels of HOPX+ cells in the cell lines harboring a PDH mutation may be associated with defects in cellular fate specification at this time point.

**Fig. 4.**
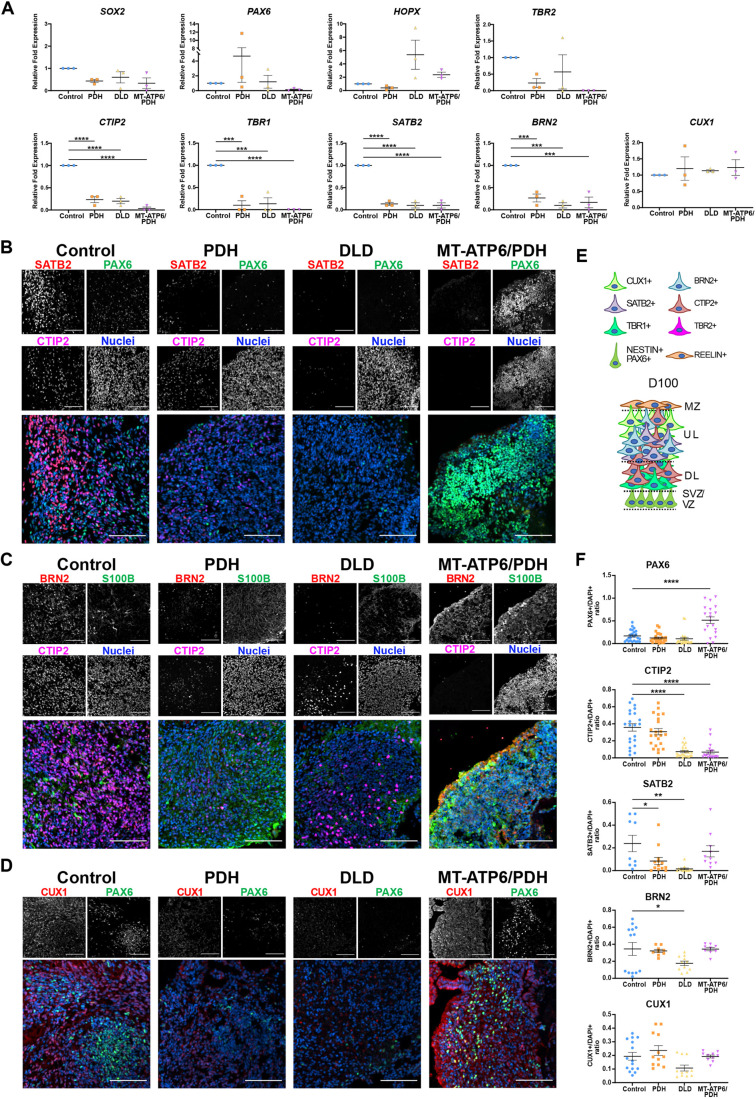
**Leigh syndrome patient-derived brain organoids show defects in cortical layer formation at day 100.** (A) RT-qPCR quantification. NPC populations were evaluated by the expression of *SOX2* and *PAX6*. IPCs were identified with the marker *TBR2* and oRG was evaluated by the expression of *HOPX*. Markers *CTIP2*, *TBR1*, *SATB2*, *BRN2* and *CUX1* were assessed for cortical development. (B-D) Representative confocal images. LS patient-derived brain organoids present reduced expression of the upper layer markers SATB2 (B) and BRN2 (C) and deep layer marker CTIP2 (B,C). Expression of the astrocyte marker S100β was also observed in the cell lines (C). LS patient-derived brain organoids express the upper layer marker CUX1 and NPC marker PAX6 (D). Images were generated from at least three different organoids per genotype from independent organoid batches. (E) Schematic of the expected organization of the brain organoids at day 100. (F) Quantification of immunofluorescence staining. Upper layer marker SATB2 was significantly reduced in PDH mutant. DLD mutant presented reduced expression of the cortical layer markers CTIP2, SATB2 and BRN2. The double mutant MT-ATP6/PDH showed a significant increase in the PAX6+ population, as well as reduced expression of the cortical plate marker CTIP2. Data are shown as mean±s.e.m. *P<0.05; **P<0.01; ***P<0.001; ****P<0.0001 (ordinary one-way ANOVA with a Dunnett's multiple comparisons test post-hoc). DL, deep layers; MZ, marginal zone; sVZ, subventricular zone; UL, upper layers; VZ, ventricular zone. Scale bars: 100 μm (B-D).

To assess cortical layer fate specification during development, we grew cerebral organoids until day 100 and probed for upper cortical layer markers (Florio and Huttner, 2014; Lui et al., 2011; Saito et al., 2011). RT-qPCR analysis of the gene expression at this time point showed no significant differences in expression of NPC markers *SOX2*, *PAX6*, oRG marker *HOPX* and IPC marker *TBR2*. Major dysregulation was observed in the neuronal markers (Fig. 4A). Cortical layer markers *CTIP2* (*P*<0.0001 in all cases), *TBR1* (PDH and DLD: *P*=0.0002, MT-ATP6/PDH: *P*<0.0001), *SATB2* (*P*<0.0001 in all cases) and *BRN2* (*POU3F2*; PDH: *P*=0.0005, DLD: *P*=0.0001, MT-ATP6/PDH: *P*=0.0002) were significantly reduced at this time point. Pan-neuronal marker *TUBB3* (PDH: *P*=0.0013, DLD: *P*=0.0002, MT-ATP6/PDH: *P*=0.0003) was also lower for all three mutants, suggesting a reduced capacity of commitment to a neuronal fate. Interestingly, the neuronal marker *CUX1* did not show significant differences in expression among cell lines. Although CUX1 is predominantly expressed in pyramidal neurons of the upper layers II-IV of the developing cortex (Leone et al., 2008; Nieto et al., 2004), its expression has been reported in the sVZ (Nieto et al., 2004) and cortical plate (Saito et al., 2011). It has also been reported as being co-expressed with PAX6+ and TBR2+ cells (Cipriani et al., 2015). Owing to the reduced expression of the cortical and neuronal markers, the maintained expression of *CUX1* could reflect its conserved expression in NPC and IPC populations rather than in committed upper-layer neurons.

Quantification of the immunofluorescence images (Fig. 4B-F) showed that the late-born superficial layer marker SATB2 (layer IV) was reduced in PDH (*P*=0.0305) and DLD (*P*=0.0013) organoids (Fig. 4B,F). Cortical layer III marker BRN2 was reduced in the DLD mutant (*P*=0.0455, Fig. 4C,F). CTIP2+ cells were also reduced in DLD and MT-ATP6/PDH organoids (*P*<0.0001 in both cases, Fig. 4B,C,F). On day 100, MT-ATP6/PDH organoids had a significant increase in PAX6+ cells (*P*<0.0001, Fig. 4B,C,F) suggesting an aberrant persistence of NPCs and lack of commitment to neuronal cell fate.

**Fig. 5.**
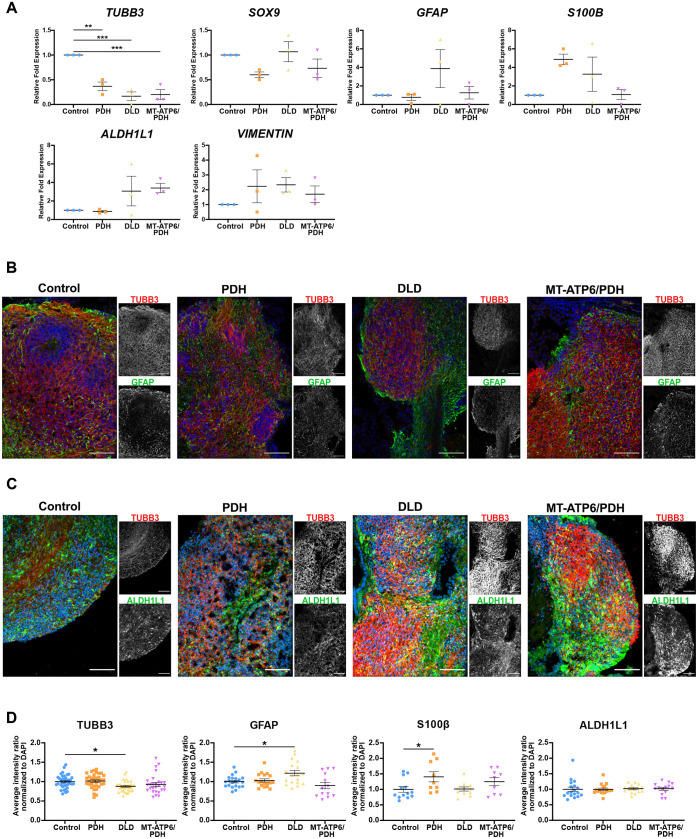
**Leigh syndrome patient-derived derived brain organoids show dysregulation of neuronal and glial markers at day 100.** (A) RT-qPCR analysis of neuronal and astrocytic genes. Neuronal marker *TUBB3* and astrocytic markers *SOX9*, *GFAP*, *S100B*, *VIM* and *ALDH1L1* were evaluated. A significant decrease in the expression of the neuronal marker *TUBB3* was observed. Fold change normalized to GPI and GAPDH as housekeeping genes. (B,C) Immunofluorescence images of astrocytic markers GFAP (B) and ALDH1L1 (C), and neuronal marker βIII tubulin (TUBB3). Nuclei in the merged image correspond to the blue channel. Scale bars: 100 μm. (D) Immunofluorescence quantification of neuronal and astrocytic staining. DLD mutant presented decreased staining in the neuronal marker TUBB3 and an increase in the astrocytic marker GFAP. PDH mutant shows a significant increase in the S100β+ population. Data are shown as mean±s.e.m. *P<0.05; **P<0.01; ***P<0.001; ****P<0.0001 (ordinary one-way ANOVA with a Dunnett's multiple comparisons test post-hoc).

As astrogliosis is a hallmark for LS (Lake et al., 2015), we looked at the expression of astrocyte markers at the mRNA and protein level. RT-qPCR analysis of the neuronal marker *TUBB3* revealed a marked downregulation in all three lines (PDH: *P*=0.0013, DLD: *P*=0.0002, MT-ATP6/PDH: *P*=0.0003) that may be associated with the reduction in the number of cortical neurons. Astrocyte marker *SOX9* (Sun et al., 2017) did not show major differences in expression. Analysis of other astrocyte markers such as glial fibrillary acidic protein *(GFAP*), S100 calcium-binding protein-β *(S100B*), aldehyde dehydrogenase family 1 member L1 (*ALDH1L1*) and vimentin (*VIM*) showed an increased, yet not significant, upregulation in some genotypes (Fig. 5A). In the case of DLD, all the above-mentioned markers were increased compared with the control. The double mutant MT-ATP6/PDH had increased expression of *VIM* and *ALDH1L1*, while PDH showed an increment in *S100B* and *VIM*. At a protein level, DLD and PDH cerebral organoids showed increased staining of astrocyte markers GFAP and S100β, respectively, at day 100 (Figs 4C and 5B,D). S100β was also increased in the double mutant, but the results were not statistically significant. Staining for ALDH1L1 did not show major differences between the genotypes (Fig. 5C,D). Immunofluorescence staining of the organoids for β3-tubulin showed a statistical difference between control and DLD (*P*=0.0174), but not in the other two mutants. The decrease in diversity of neuronal cell types and increase in the presence of S100β+ cells in the double mutants may suggest a switch to astrocyte fate during cortical development. Interestingly, the DLD organoids had higher GFAP staining (*P*=0.0141), which may suggest an increase in the reactivity of the astrocyte population. Upregulation of the astrocyte markers GFAP, S100β and VIM have been associated with astrocyte reactivity and in response to injury (Clarke et al., 2018; Escartin et al., 2021; Liddelow and Barres, 2017; Liddelow et al., 2017; Qi et al., 2017; Zamanian et al., 2012). Although the gene expression of these pan-reactive markers was not significant, it may suggest activation of the glial population in response to the metabolic dysregulation in LS organoids.

### LS-associated mutations disrupt the mitochondrial network in the VZ of cerebral organoids

**Fig. 6.**
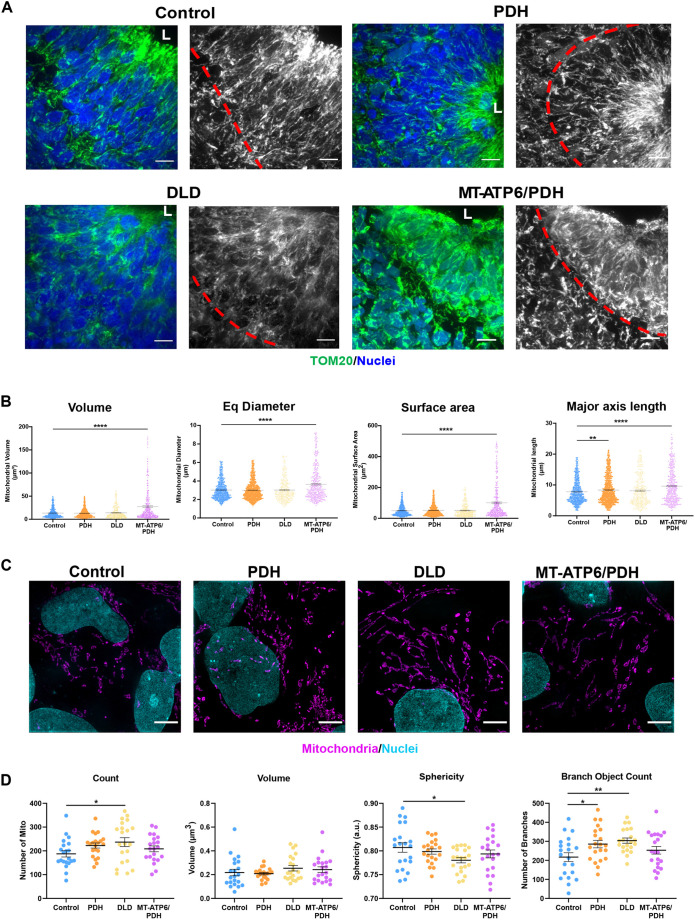
**Leigh syndrome patient-derived organoids show defects in mitochondrial morphology in the sVZ compartment.** (A) Representative confocal images of day 30 brain organoids showing mitochondrial morphology (TOM20). The red line divides the Sox2+ NPCs surrounding the lumen (L) from newly committed neurons. MT-ATP6/PDH mutant organoids showed disorganization and fragmentation of the mitochondrial network. (B) Quantification of average mitochondrial volume, diameter, surface area, and major axis length are shown. Graphs represent mean±s.e.m. from at least three independent subventricular zones (sVZs) per phenotype from three independent organoid batches. Quantification was performed by three-dimensional reconstruction of the mitochondrial network of interest. (C) Representative super-resolution images of mitochondrial morphology in LS and control NPCs. (D) Quantification of average mitochondrial number, volume, mitochondrial sphericity and mitochondrial branching are shown. Graphs represent mean±s.e.m. from at least three independent experiments (*n*>20 cells per genotype). *P<0.05; **P<0.01; ****P<0.0001 (one-way ANOVA). Scale bars: 10 μm (A); 5 μm (C).

Mitochondria in murine NPCs form an elongated network (Khacho et al., 2016), which fragments as cells undergo neurogenesis (Iwata et al., 2020). Mitochondrial morphology was evaluated in the VZ NPCs of the cerebral organoids. Cells positive for SOX2 demonstrated elongated mitochondrial networks that extend radially from the ventricle-like lumen (Fig. 6A). These results are significant because it is the first evidence demonstrating that mitochondrial networks are remodeled in the developing human brain as reported in the developing mouse cortex (Iwata et al., 2020; Khacho et al., 2016). PDH mutant organoid NPCs have an increased mitochondrial axis length compared with control (*P*=0.0078, Fig. 6B). As mentioned earlier, the stereotypical arrangement of the VZ was compromised in most MT-ATP6/PDH organoids. In the few areas where ventricle-like structures were identified with a conserved SOX2+ VZ, the mitochondrial network appeared to be more aggregated. This morphology was also observed in the clusters of SOX2+ cells that were scattered throughout the organoid. Quantification of the mitochondrial network for this mutant (Fig. 6B) showed increased mitochondrial volume, diameter, surface area and axis length (*P*<0.0001, in all cases), which suggests a mitochondrial aggregation phenotype in the VZ. Moreover, the difference in mitochondrial length may also correlate with the increased expression of *TBR2* observed by RT-qPCR (Iwata et al., 2020).

As NPCs generated by a dual SMAD monolayer method did not show major differences among the different genotypes, we looked at their mitochondria morphology to evaluate whether the differences observed in the organoids were recapitulated in this paradigm. Characterization and quantification of various parameters of the mitochondrial network using structured illumination microscopy (SIM) revealed that, although control human NPCs showed elongation of the mitochondrial network, the DLD mutant displayed an increase in mitochondrial number and decreased sphericity. Both DLD and PDH mutants had a significant increase in the number of branches in the network (Fig. 6C,D), which may reflect an increase in fusion events (Sukhorukov et al., 2012; Westrate et al., 2014). These changes suggest that the mitochondrial network in DLD and PDH lines may be more fragmented than in the control, which could be linked to the underlying changes in metabolic capacity (Rafelski, 2013) and developmental defects (Westrate et al., 2014).

### Metabolic dysregulation in LS-derived cerebral organoids

**Fig. 7.**
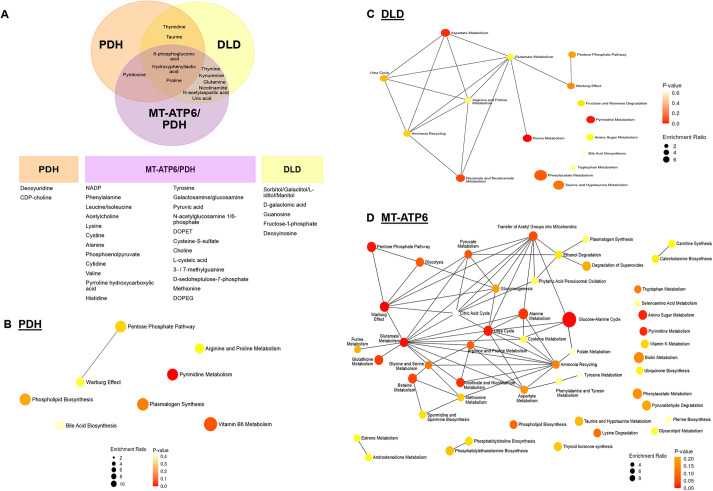
**Day 40 LS organoids show changes in their metabolic profiles.** (A) A total of 43 metabolites were dysregulated (*P*<0.05 and FDR=0.01) and segregated by affected cell line. Three batches of 40-day organoids per line (four independent organoids per line per batch) were analyzed. (B-D) Metabolite set enrichment analysis for dysregulated metabolites enriched in the PDH (B), DLD (C) and MT-ATP6/PDH mutant (D). Diameter of the node is determined by the level of enrichment and the color of the node represents the *P*-value of the interaction.

To explore changes in metabolites, we performed metabolomic profiling of day 40 organoids. Metabolomic analysis showed 43 different metabolites that were significantly dysregulated in the LS organoids (Table S2; Fig. S7). Out of these metabolites, eight were dysregulated in PDH, 16 in DLD and 32 in MT-ATP6/PDH (Fig. 7A; Fig. S7).

The metabolites proline, 6-phosphogluconic acid and hydroxyphenyllactic acid were dysregulated in all three LS mutants. High levels of proline have been associated with negative effects in brain function by interference in glutamatergic neurotransmission (Gogos et al., 1999; Vorstman et al., 2009). On the other hand, 6-phosphogluconic acid was reduced in all three LS cell lines. High concentrations of this metabolite have been associated with an active pentose phosphate pathway in early brain development in rats (Hakim et al., 1980) and its supplementation increased the diameter of neurospheres derived from the embryonic Ts1Cje mouse model of Down syndrome (Seth et al., 2020). Hydroxyphenyllactic acid was elevated in DLD and PDH mutant organoids but downregulated in MT-ATP6/PDH. High levels of this metabolite have been reported in association with high lactate and pyruvate in pediatric lactic acidosis in patients with PDHc deficiency (Kumps et al., 2002; Stern, 1994).

Pyruvate was also increased in the MT-ATP6/PDH mutant organoids, correlating with the lactic acidosis expected in the organoids based on the patient phenotypes and the presence of the PDH mutation that hinders flux from pyruvate into the TCA cycle through acetyl-CoA. Moreover, the glycolysis/gluconeogenesis intermediate phosphoenolpyruvate was also elevated in these mutants. Increased levels of phosphoenolpyruvate in rat brains after ischemic injury are thought to have a protective role in cerebral ischemia *in vivo* (Geng et al., 2021) and in oxygen/glucose deprivation *in vitro* (Jiang et al., 2019).

Besides the previously mentioned metabolites, MT-ATP6/PDH mutant organoids presented increased levels of choline, cytidine and leucine. Choline is a crucial metabolite for normal CNS development. Neural tube defects have been associated with a lack of choline during early pregnancy (Zeisel, 2006). It has also been shown to increase cell proliferation and decrease apoptosis in fetal rat hippocampal progenitor cells (Albright et al., 1999a,b; Zeisel and Niculescu, 2006). Choline is also crucial for the production of the neurotransmitter acetylcholine, the sphingolipid sphingomyelin, and myelin (Oshida et al., 2003). Concomitantly, cytidine is used with choline for the generation of cytidine-5-diphosphocholine, a crucial intermediate in the biosynthesis of the cell membrane phospholipids phosphatidylcholine and phosphatidylethanolamine (Cansev, 2006; Rema et al., 2008). Increased abundance of the branched-chain amino acid leucine has been associated with the metabolic illness maple syrup urine disease and can be extremely neurotoxic (Bridi et al., 2005; García-Cazorla et al., 2014). This amino acid is considered ketogenic as its end products can enter the TCA cycle for energy generation or act as precursors for lipogenesis and ketone body production (Manoli and Venditti, 2016).

Pathway analysis of the dysregulated metabolites (Tables S3-S5) shows overlap in pyrimidine metabolism, taurine and hypotaurine metabolism, pentose phosphate pathway, arginine and proline metabolism, and aminoacyl-tRNA biosynthesis. Pyrimidine nucleotides are essential precursors for nucleic acid synthesis and are involved in polysaccharide and phospholipid biosynthesis, detoxification processes, and protein and lipid glycosylation (Fumagalli et al., 2017). Taurine and hypotaurine are osmotic regulators in the brain, as well as agonists to GABAergic and glycinergic neurons (Albrecht and Schousboe, 2005). Its presence in the developing brain is necessary for the correct development of axons and the formation of synaptic connections (Sturman, 1993). Dysregulation of the aminoacyl-tRNA biosynthesis pathways is well documented as causal etiology for several neurodevelopmental disorders such as leukoencephalopathies, microcephaly and LS (Francklyn and Mullen, 2019; Ognjenović and Simonović, 2018).

We also performed metabolite set enrichment analysis (Fig. 7B-D). In addition to the previously mentioned pathways, the Warburg effect, or aerobic glycolysis, was also shared among the LS mutants. Although this effect is considered one of the hallmarks of cancer, it has also been associated with several homeostatic processes, including cell turnover and proliferation, and brain development (Bubici and Papa, 2019). Energy generation through aerobic glycolysis as a compensatory mechanism to overcome the metabolic deficiency in LS could suggest a survival adaptation of the cerebral organoids. Moreover, the shutoff of aerobic glycolysis is crucial to neuronal differentiation in human NPCs. Inability to transition to neuronal OXPHOS causes apoptosis due to excessive conversion of pyruvate to lactate, and potentially a cell fate shift into GFAP+ glial cells (Zheng et al., 2016b). Considering our observations that there is a marked deficit in MT-ATP6/PDH mutants to commit and generate neuronal subtypes and an increased signal in astroglial markers, these mutations may be impairing the ability to transition from aerobic glycolysis to OXPHOS as previously described with SURF mutations (Inak et al., 2021). The preferential switch to a glial fate may be promoted by astrocytes having low expression levels or lower activity levels of the PDHα subunit (Bélanger et al., 2011; Halim et al., 2010; Itoh et al., 2003; Laughton et al., 2007).

## DISCUSSION

LS is a rare inherited neurometabolic disease with more than 75 causal genes identified in both nuclear and mitochondrial DNA. It has an early onset, affecting most patients within their first year of life, although cases during teenage years and adulthood have been reported (Finsterer, 2008; Lake et al., 2016). As it is a highly heterogeneous disease, establishment of animal and *in vitro* models have been challenging and limited to only select mutations. Here, we report the characterization and the subsequent generation of brain organoids from three commercially available LS fibroblast cell lines and age-matched control.

Three-dimensional differentiation generates higher numbers of NPCs and more mature neurons than two-dimensional differentiation (Chandrasekaran et al., 2017; Di Lullo and Kriegstein, 2017; Muratore et al., 2014; Paşca et al., 2015) in part because of an improved spatial cellular environment that influences cell fate specification. We observed that all the LS cerebral organoids failed to thrive at different time points. Although organoid development initially appeared to be normal in cell lines with nuclear-encoded LS mutations, at later time points the developmental program was compromised, presumably because of a failure to generate upper-layer neurons.

Although the number of cells positive for the upper neural markers appears to be reduced, the organoids still maintain a cellular density similar to control. Further analysis of these organoids at later times of maturation using single cell RNA-sequencing (Kanton et al., 2019; Velasco et al., 2019) or mass cytometry (Brockman et al., 2021; Leelatian et al., 2017) would be useful to identify the effects of the LS-associated mutations in cortical cell fate specification.

Clinical data from LS patients report marked gliosis as part of the characteristic findings (Baertling et al., 2014, 2016; Lake et al., 2015; Schubert Baldo and Vilarinho, 2020). Although this gliosis phenotype is potentially associated with a reactive process secondary to neuronal damage, an intriguing alternate possibility is that NPCs may have an increased propensity to differentiate down the astrocyte lineage due to LS-causative mutations and mitochondrial-associated dysregulations. Previous studies have shown that reactive astrocytes acquire molecular hallmarks of radial glial cells. It was also shown through genetic fate mapping that mature astroglial cells can dedifferentiate and resume proliferation (Robel et al., 2009, 2011). Thus, the increase in the glial-specific marker S100β in PDH organoids and DLD multipotency cultures, as well as GFAP staining in DLD organoids and the upregulation, albeit not significant, in mRNA expression of the astrocyte markers, could either reflect that chronic metabolic stress induced by LS mutations activates a brain injury response, or that the inhibition of mitochondrial metabolism in NPCs could cause defects in lineage selection (Escartin et al., 2021). Owing to the differential expression and activation levels of the PDH complex in astrocytes (Bélanger et al., 2011; Halim et al., 2010), a predisposition of these cell lines to commit to an astroglial fate cannot be ruled out. Culturing these organoids for longer than 100 days is required to analyze the gliosis phenotype in more detail. Analysis of A1 specific reactivity markers may clarify whether the upregulation of *GFAP*, *S100B*, *ALDH1L1* and *VIM* is associated with a neuroinflammation response to neuronal damage (Escartin et al., 2021; Liddelow et al., 2017).

The formation of lesions in LS has been described as the result of OXPHOS dysfunction and subsequent ATP depletion. Neuronal dysfunction is suspected to trigger chronic gliosis (Baertling et al., 2016). In patients, the gliosis phenotype can be accompanied by vascular hypertrophy and the production of excess ROS, which increases neuronal damage (Lake et al., 2015). However, owing to the lack of vascularization in the organoid model, replicating the vascular abnormalities associated with LS is not feasible.

In a previous study (Hattori et al., 2016), the metabolic signature analysis of iPSCs derived from a mitochondrial-encoded LS mutation (m.10191T>C) showed differences in the abundance of pyruvate and lactate, among others. In our study, metabolomic analysis from organoids shows that the observed changes in the metabolites are in line with the clinical observations of LS patients. Changes in blood and cerebral spinal fluid concentration of lactate and pyruvate are common diagnostic tools for LS (Hattori et al., 2016) and other mitochondrial diseases (Barshop, 2004; Buzkova et al., 2018; Esterhuizen et al., 2017; Rahman and Rahman, 2018). Although changes in the NADH/NAD+ ratio, *de novo* nucleotide synthesis and in other metabolites from the ETC complex III and TCA cycle, were also identified, these were modest considering that the genetic alterations in the mutant cell lines should directly affect these pathways. This could point to metabolic compensatory mechanisms that could be engaged during development. Moreover, the disruption in the metabolic network observed in LS cerebral organoids correlates with the severity and mortality of the disease in the probands. Although aerobic glycolysis was identified as a significantly affected pathway in all the mutants, the effects of the MT-ATP6/PDH mutation reflected the importance of competent glycolysis to OXPHOS transition in early brain development.

The metabolic dysregulation of the affected tissues in LS may have a direct effect on mitochondrial morphology and function. Mitochondrial fragmentation is a hallmark of glycolytic cell types such as stem cells and cancer cells (Chen and Chan, 2017; Rastogi et al., 2019). Moreover, neurogenesis defects have been observed in the context of mitochondrial morphology dysregulation and are considered to be upstream regulators of self-renewal and cell fate decisions in stem cells (Iwata et al., 2020; Khacho et al., 2016). Also, the capacity of cells to undergo a metabolic switch during neurodevelopment is crucial for their survival and correct fate determination (Zheng et al., 2016b). The double mutant MT-ATP6/PDH showed a reduced energetic capacity in both iPSC and NPC stages that does not appear to affect their ability to differentiate into the three neural lineages nor increase their sensitivity to apoptotic stimuli. The MT-ATP6/PDH NPCs did not show major alterations of the mitochondrial network in two-dimensional cultures.

Energetic requirements have been shown to directly impact the capacity of NPCs to survive, migrate and differentiate (Zanotelli et al., 2018; Zanotelli et al., 2019). The effects of LS-causing mutations on mitochondrial network integrity and overall development of the neural lineage became more apparent in the three-dimensional systems. Tissue architecture, mechanical cues, cell-to-cell communication, nutrient accessibility, oxygen tension and morphogen gradients characteristic of three-dimensional systems help to recapitulate the microenvironment in the developing CNS in a manner that is not supported by two-dimensional neural differentiations (Pampaloni et al., 2007; Tibbitt and Anseth, 2012).

To our knowledge, this is the first time that mitochondrial morphology in the cortex has been analyzed in a human model system of LS brain development, and it highlights the crucial function of mitochondrial network plasticity for the proper specification of cell fate and survival. However, limitations in mitochondrial segmentation and resolution when using conventional confocal microscopy need to be addressed. Large interconnected areas of mitochondrial network can be mistaken with aggregated mitochondria, especially in high cellular density areas, due to limitations in the spatial resolution and thresholding of the images. However, increased accessibility to super resolution microscopy, two-dimensional and three-dimensional structured illumination microscopy, high content imaging, improved artificial intelligence and machine learning approaches may resolve these challenges (Chaudhry et al., 2020; Jakobs, 2006; Leonard et al., 2015).

Recent studies have shown (Paulsen et al., 2022) that human brain organoid models could be used to identify cell-type-specific developmental abnormalities that converge in a similar phenotype. Our study is significantly limited by the small sample number, a common challenge in the rare disease field. Thus, there is an urgent need of optimizing current approaches to streamline mitochondrial gene editing protocols, which would allow engineering several human iPSC and embryonic stem cell lines with mitochondrial-related mutations commonly found in patients. These advancements are a needed next step in the field of rare mitochondrial diseases. However, despite the heterogeneity of the cells used in this study, the results may have uncovered potentially common neurodevelopmental abnormalities shared across mitochondrial diseases caused by diverse mutations (Inak et al., 2021). As demonstrated recently (Paulsen et al., 2022), organoid models are useful biological tools to identify points of convergence in the neurobiological basis of mutations contributing to the pathology of complex diseases, such as rare mitochondrial diseases.

Taken together, our study sheds new light on the morphological and functional LS alterations impacting early events of neurogenesis. We identified new genetic alterations in LS samples using WES and mitochondrial DNA sequencing. We described the effects of LS mutations on early development, underscoring the crucial function of metabolism in human neurogenesis. Our work also provides a comprehensive phenotypic characterization of available patient samples to encourage their use as model systems for uncovering the mechanisms underlying neuronal cell death in the context of LS and as human platforms for drug discovery.

## MATERIALS AND METHODS

### Experimental model and subject details

The Coriell cell line IDs were as follows: GM01503, GM03672, GM1341. Information about the LS cell lines used in this study can be found in Table S1. Control skin fibroblast cell line AG16409 was also obtained from the Coriell Institute and analyzed for contamination. The donor was a 12-year-old apparently healthy Caucasian male. Cells were negative for mycoplasma.

Fibroblasts were maintained in Dulbecco's Modified Eagle Medium: Nutrient Mixture F-12 (DMEM/F-12; Gibco, 11330032) supplemented with 10% fetal bovine serum (Sigma-Aldrich, F2442) in 100 mm cell culture plates (Eppendorf, 0030702115) in a 37°C 5% CO_2_ incubator.

### Whole-exome sequencing

Fibroblast cell pellets from each cell line (>1 million cells) were shipped on dry ice for WES to Genewiz. The Illumina HiSeq-X was used to perform 150 nt paired-end sequencing.

### Mitochondrial sequencing

Fibroblast cell pellets from each patient (>1 million cells) were shipped on dry ice for mitochondrial sequencing to Girihlet. The sequencing configuration used was 80 bp single-end sequencing, 20 million reads per sample.

### Human iPSC generation and characterization

Human fibroblasts from healthy controls and patients were purchased (Coriell Institute). iPSCs were derived from human fibroblasts using a Sendai virus-based reprogramming kit (CytoTune-iPS Sendai Reprogramming Kit, A13780-01, Thermo Fisher Scientific), according to the manufacturer's instructions. After 3-4 weeks, 2-3 colonies per sample were transferred to fresh six-well plates and were expanded and gardened for three passages before freezing. All iPSC cell lines were maintained in E8 medium (Chen et al., 2011) in plates coated with Matrigel (Corning, 354277) at 37°C with 5% CO_2_. Culture medium was changed daily. Cells were checked daily for differentiation and were passaged every 3-4 days using Gentle Cell Dissociation Solution (StemCell Technologies, 07174). All experiments were performed under the supervision of the Vanderbilt Institutional Human Pluripotent Cell Research Oversight (VIHPCRO) Committee. Cells were checked for contamination periodically.

### Analysis of pluripotency

The pluripotency of each iPSC clone was determined using a microarray-based tool known as PluriTest (Thermo Fisher Scientific, A38154) as an alternative to the teratoma assay. Samples were outsourced to Thermo Fisher Scientific for PluriTest and further analysis. Low passage iPSC cell pellets (>1 million cells) were frozen and shipped on dry ice. In addition, the expression of pluripotency genes *POU5F1* and *NANOG* was assessed by qPCR.

### Analysis of chromosomal abnormalities

The presence of any chromosomal abnormalities in the newly generated iPSCs was determined using a microarray-based tool known as KaryoStat (Thermo Fisher Scientific, A38153) as an alternative to chromosomal G-banding. Low passage iPSC pellets (>1 million cells) were frozen and shipped on dry ice to Thermo Fisher Scientific for KaryoStat and further analysis.

### Trilineage differentiation

The STEMdiff Trilineage differentiation kit (StemCell Technologies, 05230) was used to functionally validate the ability of newly established iPSCs to differentiate into three germ layers, as per the manufacturer's instructions. Single-cell suspensions of 2×10^6^ cells/well, 5×10^5^ cells/well, 2×10^6^ cells/well were seeded for ectoderm, mesoderm and endoderm, respectively, in their corresponding medium at day 0 in six-well plates. The cultures were maintained for 7 days, 5 days and 5 days for ectoderm, mesoderm and endoderm, respectively. The differentiation was assessed by qPCR.

### NPC differentiation and multipotency characterization

For monolayer differentiation of the iPSCs into NPCs, cells were dissociated into single cells using Gentle Cell Dissociation Reagent (StemCell Technologies, 07174) for 8 min at 37°C. Live cell counts were performed using Trypan Blue (0.4%) staining (Invitrogen, T10282) using a Countess Automated Cell Counter. Cells were then seeded in a Matrigel-coated six-well plate (Eppendorf, 0030720113) to 2.5×10^6^ cells/well in STEMdiff™ SMADi Neural Induction Medium (StemCell Technologies, 08581) (Chambers et al., 2009) supplemented with ROCK inhibitor. Daily media changes were performed and passaging of the cells was carried out every 7-9 days. Cells for NPC marker analysis were collected at the end of the first 9 days of differentiation.

For multipotency analysis, culture media was changed to NeuroCult medium (StemCell Technologies, 05751) and maintained for 4 weeks. Samples were then fixed and stained for the neuronal marker TUBB3 and the oligodendrocyte progenitor marker OLIG2. Astrocyte differentiation was performed by seeding on a Matrigel-coated plate 1.5×10^6^ cells/cm^2^ (TCW et al., 2017). The following day, the media was changed to Astrocyte medium (ScienCell, 1801) and maintained for 20 days. Full media changes were carried out every 2 days. Samples were then fixed and stained for the astrocyte marker S100β. Images were acquired using a Nikon Instruments Ti2 inverted fluorescence widefield microscope equipped with a Plan Apo Lambda 20×0.75 NA objective, DS-Qi2 camera (Nikon Instruments) and X-Cite 120LED light source (Excelitas). The differentiation was also assessed by qPCR.

### NR differentiation

To generate NRs, we dissociated the cells into a single-cell suspension and seeded 3.0×10^6^ cells/well of an AggreWell 800 in dual SMAD inhibitor media. EBs were incubated at 37°C with 5% CO_2_, with minimal disruption during the first 48 h. Media changes, 50-75% of the total volume, were performed every 2 days. On day 5, EBs were harvested according to the manufacturer's protocol and transferred to a 35 mm imaging plate (Cellvis, D35-14-1.5-N) coated with Matrigel. Daily media changes were performed up to day 9, when cells were fixed with 100% ice-cold methanol (Thermo Fisher Scientific, A454-4). Images were acquired on a Nikon Instruments Ti2 inverted fluorescence microscope, equipped with a Yokogawa X1 spinning disk head, Andor DU-897 EMCCD, Plan Apo Lambda 0.75 NA 20× objective for representative figures, and Plan Fluor 0.45 NA 10× objective for NR quantification, piezo Z-stage, as well as 405-, 488-, 561- and 647-nm lasers. Acquisition and analysis were performed using NIS-Elements software (Nikon Instruments). NR quantification was accomplished by scripting a segmentation-based image analysis routine to detect, enumerate and measure rosette lumen area based on the ZO-1 signal. Briefly, max intensity projections of each field were generated, followed by GPU-based denoising of the resulting image. Intensity-based thresholding was then applied based on criteria established for ZO-1 signal segmentation using control images. Restrictions on resultant binaries were implemented to throw out binaries intersecting image borders, morphometries deviating severely from rosette-associated geometries, as well as for those not meeting minimum size requirements. This routine could be run in batch across many image stacks to increase the sample size and robust nature of the data. Measured data was exported to Excel for further analysis.

### Cerebral organoids

Cerebral organoids were generated as described in Romero-Morales et al. (2019 preprint) with some modifications. Briefly, organoids were generated using the STEMdiff™ Cerebral Organoid Kit (StemCell Technologies, 08571, 08570). iPSCs were dissociated into single cells using Gentle Cell Dissociation Reagent (StemCell Technologies, 07174) for 8 min at 37°C. Homogeneous and reproducible EBs were generated using a 24-well plate AggreWell 800 (StemCell Technologies, 34815). On day 7, high-quality EBs were embedded in Matrigel. On day 10, the Matrigel coat was broken by vigorously pipetting up and down and the healthy organoids were transferred to a 60 mm low attachment culture plate (Eppendorf, 003070119). The plates were then moved to a 37°C incubator and to a Celltron benchtop shaker for CO_2_ incubators (Infors USA, I69222) set at 85 rpm. Full media changes were performed every 3-4 days. Transmitted-light images were acquired using an EVOS^®^ XL Core Imaging System. The software used for processing was ImageJ.

For qPCR, all organoids were pooled together for RNA extraction. Day 40 cerebral organoids were used for metabolomics, four organoids per genotype were run and analyzed individually.

### Organoid tissue preparation and immunohistochemistry

Tissue preparation was performed as described in Romero-Morales et al. (2019 preprint). Briefly, organoids were fixed in 4% paraformaldehyde in phosphate buffered saline (PBS), washed three times with PBS, and then incubated in 30% sucrose solution overnight at 4°C. Organoids were embedded in 7.5% gelatin/10% sucrose solution (Sigma-Aldrich, G1890-100G and S7903-250G) and sectioned using a cryostat (Leica CM1950) at 15 μm thickness. For immunostaining, slides were washed with PBS before permeabilization with 0.2% Triton X-100 in PBS for 1 h. Tissues were blocked with blocking medium consisting of 10% donkey serum in PBS with 0.1% Tween-20 (PBST) for 30 min. Incubation with primary and secondary antibodies was carried out using standard methods (for details see Table S6). Confocal images of the organoids were acquired using the aforementioned spinning disk microscope with Plan Fluor 10×0.45 NA and Plan Apo Lambda 0.75 NA 20× objectives (macrostructures) and Apo TIRF 1.49 NA 100× objective (mitochondria imaging). NIS-Elements software was used for image acquisition and rendering.

### Bioenergetics assay (Seahorse assay)

The Seahorse Cell Mito Stress Test (Agilent, 103015-100) was conducted to assess mitochondrial function as described previously (Joshi et al., 2020). Human iPSCs were replated in E8 medium and human NPCs were replated in in STEMdiff SMADi Neural Induction medium at 8.0×10^4^ cells/well on Seahorse XF96 cell culture microplates (Agilent) 48 h before the assay. A minimum of six technical replicates per cell line was used per assay. One day before the assay, Seahorse XFe96 extracellular flux assay cartridge (Agilent) was hydrated with 200 μl/well of water in a non-CO_2_ incubator overnight.

On the day of the assay, Seahorse XF Calibrant was added to Seahorse XFe96 extracellular flux assay cartridge for 1 h before loading the drug treatments. Seahorse medium (Agilent) with 1 mM pyruvate, 2 mM glutamine and 10 mM glucose warmed to 37°C was added to the cells 1 h before the assay and the plate was incubated in a non-CO_2_ incubator. Appropriate concentrations of oligomycin (1.5 µM), FCCP (1.5 µM) and Rot/AA (0.5 µM) were added to Seahorse XFe96 extracellular flux assay cartridge and cartridge was loaded into XF Extracellular Flux Analyzer. After a calibration step, the cell plate was loaded into the XF Extracellular Flux Analyzer to assess mitochondrial function. Resulting data was analyzed using GraphPad PRISM.

### Mitochondrial imaging and quantification

Mitochondrial imaging was performed by fixing the NPCs at 90% confluency and staining with anti-mitochondria (Abcam, ab92824, 1:200 dilution). SIM was accomplished in 3D-SIM mode on a Nikon Instruments N-SIM, equipped with an Apo TIRF 100× SR 1.49NA objective, DU-897 EMCCD camera (Andor), 405 nm and 561 nm lasers. Images presented herein are maximum intensity projections after image stacks were first acquired (five phase shifts and three rotations of diffraction grating, 120 nm/axial step via piezo) and subsequent stack reconstruction in NIS-Elements. Other than linear intensity scaling, no further image processing was performed post-reconstruction.

For the mitochondrial imaging in brain organoids, confocal images of the organoids were acquired using the aforementioned spinning disk microscope with Apo TIRF 1.49 NA 100× objective. NIS-Elements software was used for image acquisition and rendering.

NIS-Elements General Analysis (GA3) was used for the post-processing and quantification. For monolayer NPC differentiation, mitochondrial quantification was performed as described in Rasmussen et al. (2020). Briefly, quantification was performed by segmenting mitochondria in three-dimensions and skeletonization of the resulting three-dimensional mask. For the organoid mitochondrial quantification an area segmentation was performed to analyze the mitochondria in the overlapping SOX2+ area. Several parameters such as skeleton major axis and sphericity were exported into Excel. Post-processing was performed using GraphPad Prism 9, including the robust regression and outlier removal (ROUT) method for the day 30 organoids, with a false discovery rate (FDR) of 1%.

The workflows used for each condition can be found in Figs S8 and S9.

### RNA extraction and synthesis of cDNA

Cells cultured in six-well plates were collected after a wash with PBS, using 600 μl Trizol reagent. The samples were spun down at 12,000 ***g*** after the addition of 130 μl of chloroform and incubated at room temperature for 3 min. The aqueous phase of the sample was collected 200 μl at a time until reaching the edge of phase separation. RNA precipitation was carried out by incubating with 300 μl of isopropanol for 25 min, followed by centrifugation at 12,000 ***g*** for 10 min at 4°C. The RNA pellet was washed with ethanol, semi-dried and resuspended in 30 μl of DEPC water. After quantification and adjusting the volume of all the samples to 1 μg/μl, the samples were treated with DNAse (New England Biolabs, M0303), and 10 μl of this volume was used to generate cDNA using the manufacturer's protocol (Thermo Fisher Scientific, 4368814). For RNA isolation from brain organoids, the same protocol mentioned above was followed with the volumes adjusted for 1 ml of Trizol.

### RT-qPCR

We used 1 μg of cDNA sample to run RT-qPCR for the primers mentioned in Table S6. QuantStudio 3 Real-Time PCR machine, SYBR green master mix (Thermo Fisher Scientific, 4364346) and the manufacturer's instructions were used to set up the assay.

### Immunocytochemistry

Cells were fixed with 4% paraformaldehyde (Electron Microscopy Sciences, 15710-S) in PBS for 20 min at 4°C. Blocking and permeabilization were carried out in 5% donkey serum (Jackson ImmunoResearch, 017-000-121)+0.3% Triton X-100 (Sigma-Aldrich, T9284) in Tris-buffered saline for 1 h at room temperature. After this, cells were incubated with primary antibodies at the concentrations stated in Table S6 overnight at 4°C. After incubation, the cells were washed three times with PBS and then incubated with the corresponding secondary antibodies for 1 h. Cells were mounted in Vectashield (Vector Laboratories, H-1000) before imaging.

### Western blotting

Cultured cells were lysed in 1% Triton buffer containing PMSF (Thermo Fisher Scientific, 36978), PhosSTOP (Roche, 4906837001) and protease inhibitor cocktail (Roche, 4693132001). Protein concentrations were determined using the bicinchoninic acid (BCA) method (Thermo Fisher Scientific, 23227). Gel samples were prepared by mixing 30 μg of protein with LDS sample buffer (Life Technologies, NP0007) and 2-mercaptoethanol (Bio-Rad, 1610710) and boiled at 95°C for 5 min. Samples were run on 4-20% Mini-PROTEAN TGX precast gels (Bio-Rad, 4561096) and transferred onto polyvinylidene difluoride (PVDF) membrane (Bio-Rad, 1620177) overnight at 4°C. Membranes were blocked in 5% milk in Tris-buffered saline with 0.1% Tween (TBST) before primary antibody incubation. Antibodies used for western blotting are described in Table S6.

### Cell titer blue assay

After the 24 h exposure to individual treatments of 50 μM etoposide, 80 μM CCCP, 100 ng/ml nocodazole and 5 ng/ml neocarzinostatin, 20 μl of Cell Titer Blue reagent from Cell Titer Blue assay (Promega, G8081) was added to each well of a 96-well plate. Background fluorescence was calculated by adding 10% Triton in PBS to at least three wells without cells. The fluorescence generated by the reduction of resazurin to resorufin by live cells was measured using a Beckman coulter DTX 880 multimode plate reader (Beckman Coulter; 570/600 nm).

### Metabolomics analysis

Day 40 brain organoids, at least four individual organoids per genotype, were collected, rinsed with ice-cold sterile 0.9% NaCl and flash-frozen in liquid nitrogen. For metabolite extraction, cells were resuspended in 225 μl of cold 80% HPLC-grade methanol/20% HPLC-grade water per 1×10^6^ cells. After resuspension, cells were flash-frozen in liquid nitrogen and thawed rapidly in a 37°C water bath three times. Next, debris was removed by centrifugation at max speed in a tabletop microcentrifuge at 4°C for 15 min. Metabolite-containing supernatant was transferred to a new tube, dried and resuspended in 50% acetonitrile while the pellet was used for protein quantification. Samples were analyzed using Ultra-High-Performance Liquid Chromatography and High-Resolution Mass Spectrometry and Tandem Mass Spectrometry (UHPLC-MS/MS). Specifically, the system consisted of a Thermo Q-Exactive in line with an electrospray source and an Ultimate3000 (Thermo Fisher Scientific) series HPLC consisting of a binary pump, degasser and auto-sampler outfitted with an Xbridge Amide column (Waters; dimensions of 4.6 mm×100 mm and a 3.5 μm particle size). Mobile phase A contained 95% (vol/vol) water, 5% (vol/vol) acetonitrile, 10 mM ammonium hydroxide, 10 mM ammonium acetate (pH 9.0); and mobile phase B was 100% acetonitrile. The gradient was as follows: 0 min, 15% A; 2.5 min, 30% A; 7 min, 43% A; 16 min, 62% A; 16.1-18 min, 75% A; 18-25 min, 15% A with a flow rate of 400 μl/min. The capillary of the electrospray ionization (ESI) source was set to 275°C, with sheath gas at 45 arbitrary units, auxiliary gas at 5 arbitrary units and the spray voltage at 4.0 kV. In positive/negative polarity switching mode, an m/z scan range from 70 to 850 was chosen, and MS1 data was collected at a resolution of 70,000. The automatic gain control (AGC) target was set at 1×10^6^ and the maximum injection time was 200 ms. The top five precursor ions were subsequently fragmented, in a data-dependent manner, using the higher energy collisional dissociation (HCD) cell set to 30% normalized collision energy in MS2 at a resolution power of 17,500. Data acquisition and analysis were carried out using Xcalibur 4.1 software and Tracefinder 4.1 software, respectively (both from Thermo Fisher Scientific). The peak area for each detected metabolite was normalized by the total ion current, which was determined by the integration of all of the recorded peaks within the acquisition window.

Normalized data was uploaded to MetaboAnalyst (https://www.metaboanalyst.ca/home.xhtml) for analysis. Samples were normalized to control, and a one-way ANOVA was performed to compare between the groups. Fisher's least significant difference method (Fisher's LSD) was performed as a post-hoc comparison. Enrichment and pathway analysis was also performed using this platform.

### Bioinformatic analysis

Bioinformatic analysis began with Variant Call Format (VCF) files provided by Genewiz (see ‘Whole-exome sequencing’ section), both for SNPs and indels. SnpSift version 4.3t (Cingolani et al., 2012) was used to process and filter these files for downstream analysis. Details extracted included gene symbol, Entrez gene ID and name, UniProt ID, Ensembl ID, chromosome and position, reference variant, alternative variant, quality of the call, allele name, type of SNP, impact of the SNP and the genotype of each sample. From these filtered outputs, we generated SNP/indel reports that allowed us to look at sample-specific SNPs and indels, as well as perform aggregate-level functions for grouping and statistical analysis.

To generate the SNP/indel circular chromosome plots, the top 20 genes that had variants in all three samples were plotted, ranked by frequency of variants per gene. The outside track is used to visualize the chromosomes and marked gene locations. For each sample, we used a single track to show the variant frequency as a circular scatter plot, with the height of the scatter points representative of the variant quality metric, which is a Phred-scaled probability that a REF/ALT polymorphism exists at the variant site. We used the same approach for visualization of SNPs in the mitochondrial chromosome.

### Quantification and statistical analysis

No statistical methods were used to pre-determine sample sizes. All experiments were performed with a minimum of three biological replicates unless specified. Statistical significance was determined by one- or two-way ANOVA as appropriate for each experiment. GraphPad Prism v8.1.2 was used for all statistical analysis and data visualization. Error bars in all scatter dot plot graphs represent the standard error of the mean or standard deviation as described for each Figure.

For NR experiments, the region of interest (ROI) was randomly selected using the nuclear (DAPI) staining channel. Images were processed with NIS-Elements software with our ‘Neural rosette lumen identification’ macro. Outliers were removed from the NR area analysis as post-processing quality control for the NIS-Elements macro using GraphPad Prism v8.1.2. The ROUT method was used with an FDR of 1%.

For cerebral organoid experiments, four independent batches were generated. At day 30 and day 100, at least five organoids per cell line were collected. Immunofluorescence images of at least three independent organoids were acquired per condition slide. Image processing was carried out using NIS-Elements and Fiji software.

Organoid efficiency evaluation was performed on day 10 using 4× transmitted-light images acquired using an EVOS XL microscope. Two observers were blinded to the cell line identifier and counted the number of normal and defective (no epithelial buds or more than 75% of the area is not developed) organoids. Criteria for normal and defective organoids was based on Lancaster and Knoblich (2014).

For the organoid quantification, images were processed with NIS-Elements using the GA3 tool. Three-dimensional thresholding macros were generated for each set of slides and quantified by either bright spot count (nuclear staining) or mean intensity of the ROI. To avoid false positive counts, only bright spot count where there was overlapping with DAPI was counted. GA3 analysis workflow can be found in Fig S10.

## Supplementary Material

Supplementary information

Reviewer comments
