# Supplementary material for: Human iPSC-derived cerebral organoids model features of Leigh syndrome and reveal abnormal corticogenesis
Source: Development. 2022 Jul 6;149(20):dev199914. doi: 10.1242/dev.199914 (PMC9357378; doi:10.1242/dev.199914)
Supplement: Supplementary information [file develop-149-199914-s1.pdf]

Supplemental Figure 1

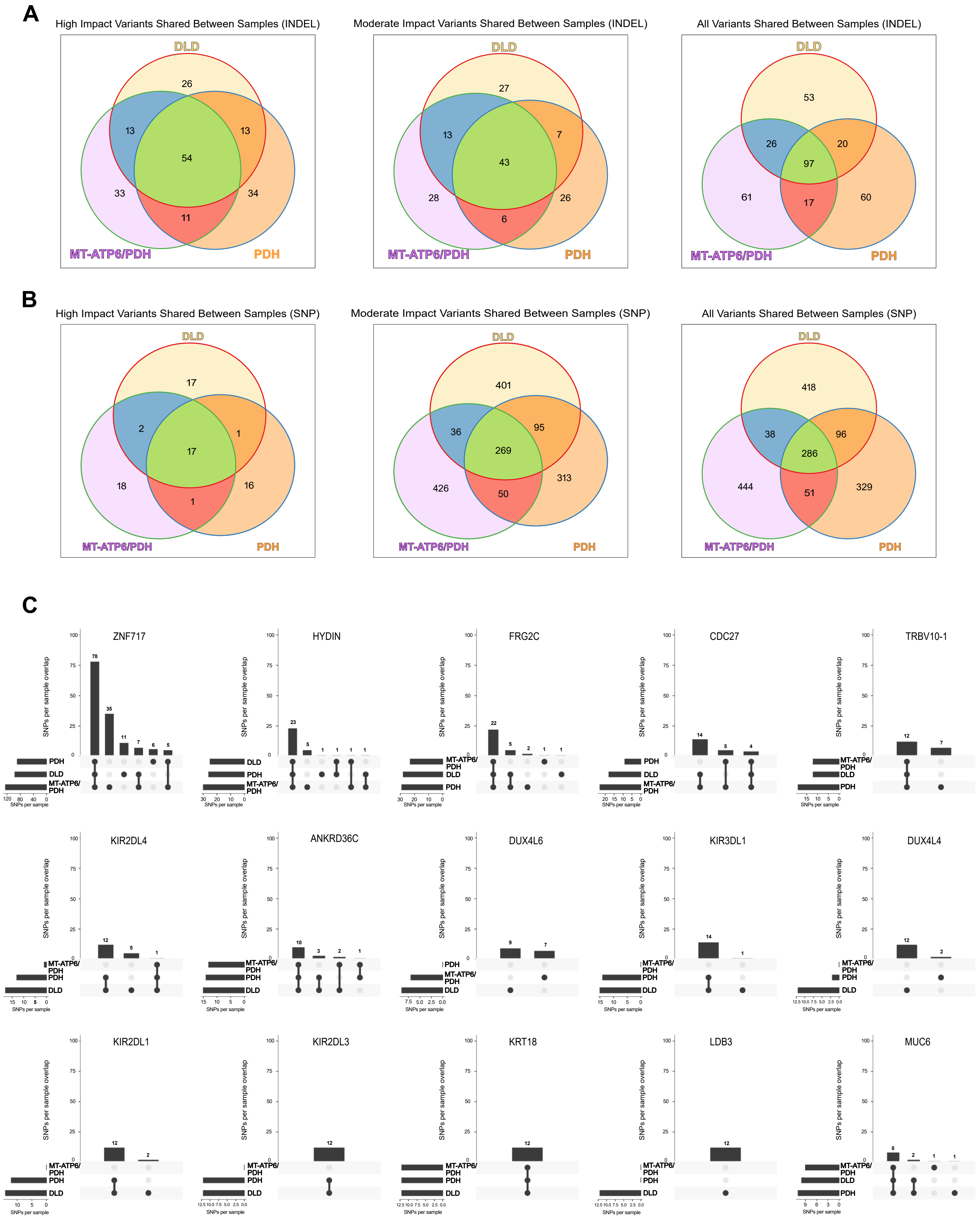

Development • Supplementary information

Fig. S1. Related to Fig. 1. A-B. Venn diagram of the distribution of high, moderate and all impact annotations across samples for INDELs (A) and SNPs (B). Pie charts of the annotation distributions within samples. C. Top 15 genes with high impact SNP variants identified in the WES analysis. Number of overlapping SNPs per sample are denoted in as vertical bars, while the number of SNPs present in each phenotype are noted in the horizontal bars. *ZNF717*: Zinc finger protein 717, *HYDIN*: HYDIN axonemal central pair apparatus protein, *FRG2C*: FSHD region gene 2 family member C, *CDC27*: Cell division cycle 27, *TRBV10-1*: T cell receptor beta variable 10-1, *KIR2DL4*: Killer cell immunoglobulin like receptor, two Ig domains and long cytoplasmic tail 4, *ANKRD36C*: Ankyrin repeat domain 36C, *DUX4L6*: Double homeobox 4 like 6, *KIR3DL1*: Killer cell immunoglobulin like receptor, three Ig domains and long cytoplasmic tail 1, *DUX4L4*: Double homeobox 4 like 4 , *KIR2DL1*: Killer cell immunoglobulin like receptor, two Ig domains and long cytoplasmic tail 1, *KIR2DL3*: Killer cell immunoglobulin like receptor, two Ig domains and long cytoplasmic tail 3, *KRT18*: Keratin 18, *LDB3*: LIM domain binding 3, *MUC6*: Mucin 6.

Development • Supplementary information

Supplemental Figure 2

A

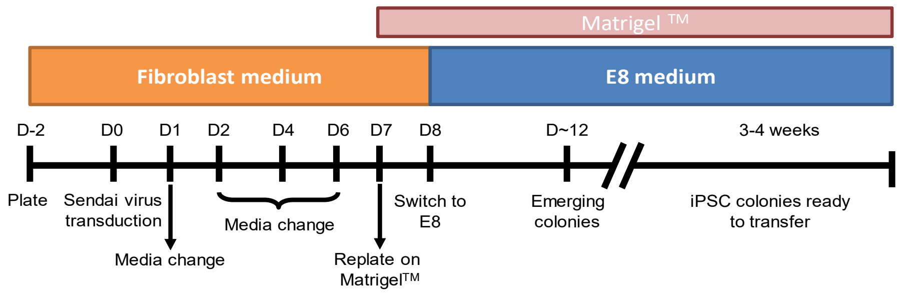

B

Sample	Pluritest Result	Pluricor	NovelCor
Control	Pass	38.62112	1.61562
PDH	Pass	37.78063	1.547368
DLD	Pass	36.43402	1.626429
MT-ATP6/ PDH	Pass	36.21865	1.509551
Non iPSC control	Fail	-48.9629	2.790166

C

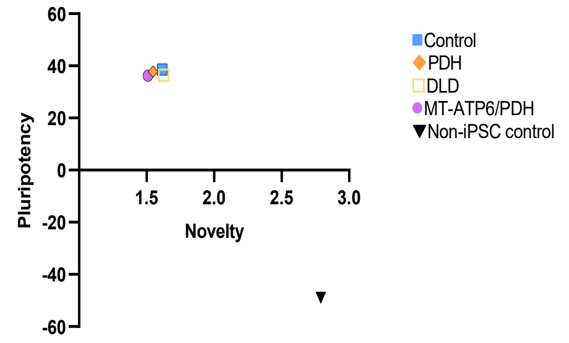

D

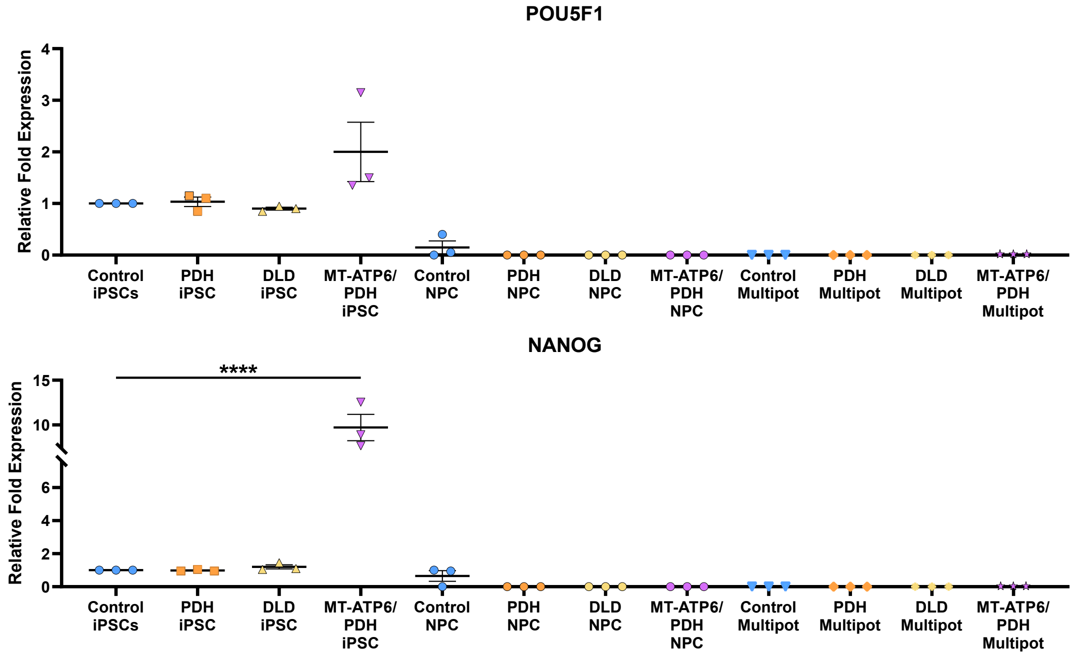

E

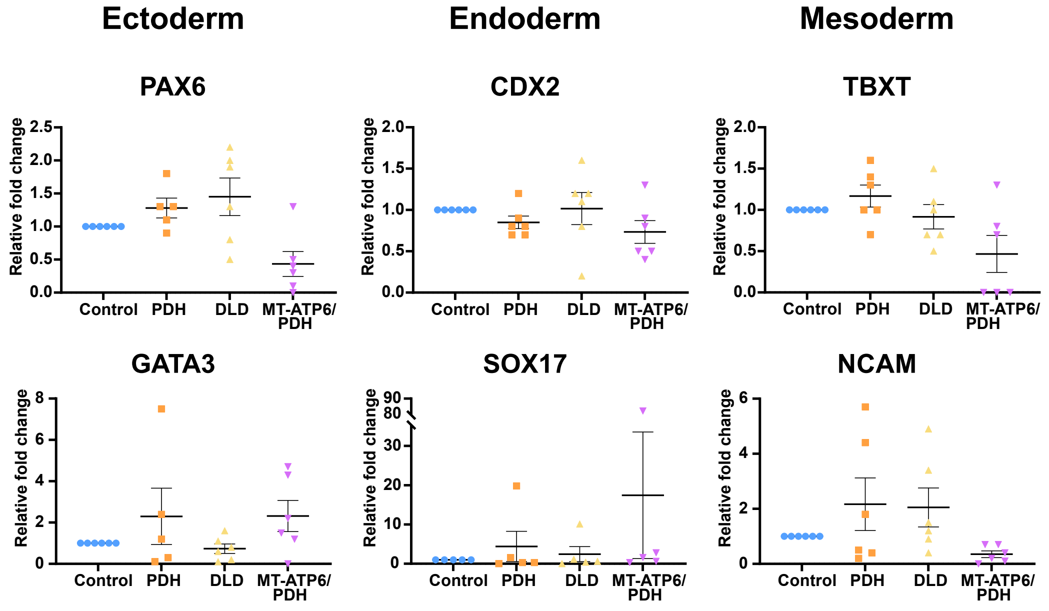

Development • Supplementary information

Fig. S2. Characterization of Leigh syndrome patient derived iPSCs. A. Schematic representation of the fibroblast reprogramming protocol. B-C. Pluripotency characterization of the LS iPSCs. Samples were analyzed against samples in a reference data set (The International Stem Cell Initiative, 2018) (B). The distribution of the samples compared with a non-iPSC control shows clustering of the samples in the high pluripotency and low novelty quadrant (C). D. qPCR for the pluripotency genes *POU5F1* and *NANOG* (p<0.001*).* E. Induced pluripotent stem cells derived from Leigh syndrome patient fibroblasts are capable of differentiation into specific lineages. RT-qPCR analysis for the ectodermal genes *GATA3* and *PAX6*, ectodermal genes *CDX2* and *SOX17* the mesodermal genes *TBXT* and *NCAM.* Fold change normalized to GPI and GAPDH as house-keeping genes. iPSC: induced pluripotent stem cells, NPC: neural progenitor cells, Multipot: neural multipotency differentiation. Graphs represent mean ± SEM from at least three independent experiments. *p<0.0uN **p<0.01N ***p<0.001N ****p<0.0001.

Development • Supplementary information

Supplemental Figure 3

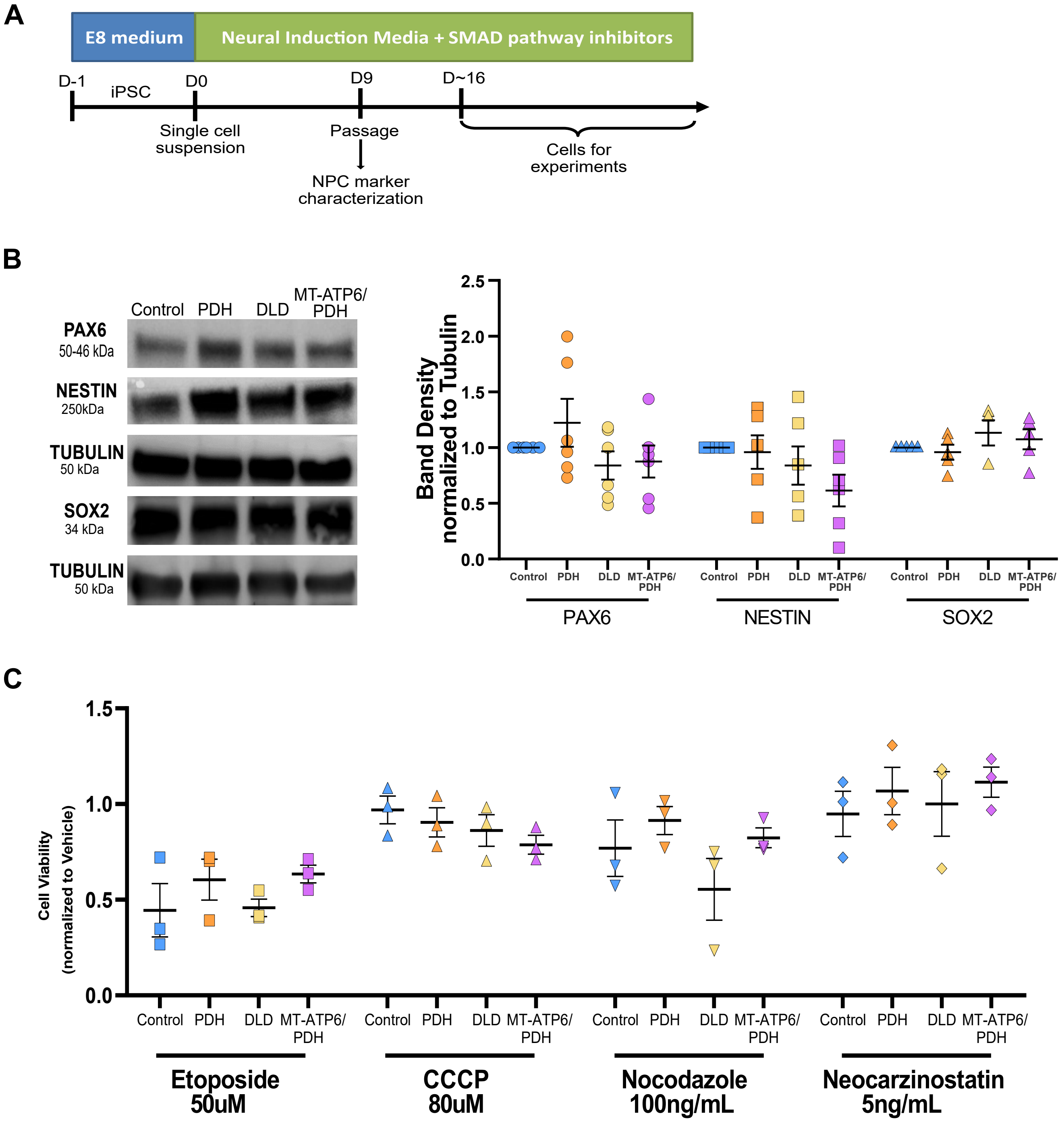

Fig. S3. Related to Fig. 2. Leigh syndrome patient derived NPCs are multipotent and do not present increased sensitivity to pharmacological stressors. A. Schematic of two-dimensional neural differentiation. B. Immunoblot of protein expression of neural markers PAX6, NESTIN, and SOX2 (Left). Quantification of protein expression of neural markers PAX6, NESTIN and SOX2. Band density normalized to TUBULIN as a loading control (Right). C. Leigh syndrome NPCs do not show enhanced sensitivity towards different stressors. LS NPCs have similar cell viability when exposed to the DNA damaging agents etoposide and neocarzinostatin, the mitochondrial toxicant CCCP, and the microtubule destabilizer nocodazole.

Supplemental Figure 4

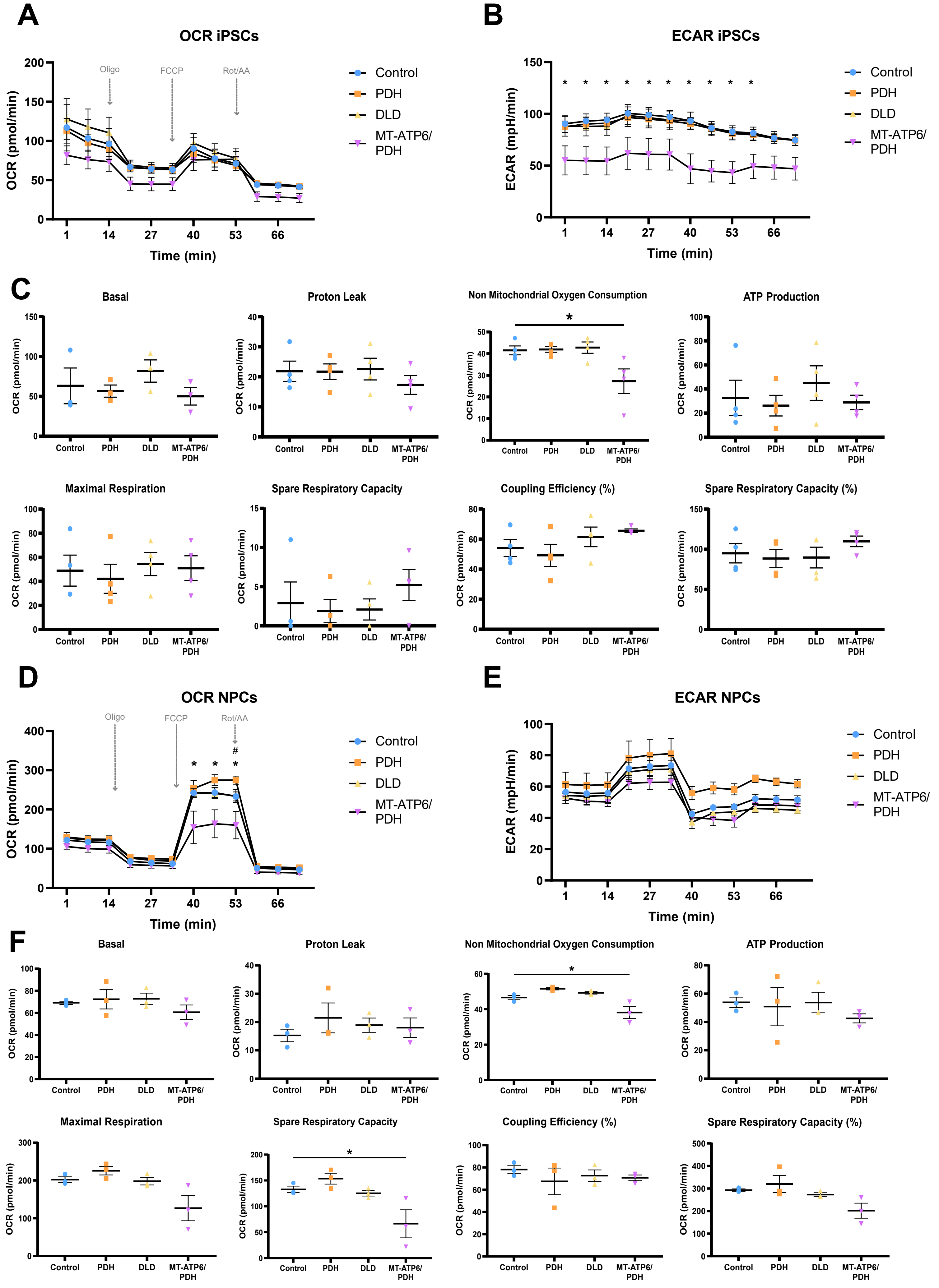

Development • Supplementary information

Fig. S4. Bioenergetic profile of LS patient derived iPSCs and NPCs. A. Analysis of oxygen consumption rate (OCR) in control and LS patient derived iPSCs. B. Extra cellular acidification rate (ECAR) for control and LS patient derived iPSCs. MT-ATP6/PDH iPSCs show reduced glycolytic capacity. C. Bioenergetic parameters for LS and control iPSCs. MT-ATP6/PDH derived iPSCs presented with reduced non-mitochondrial oxygen consumption compared to control (p=0.0284). D. Analysis of oxygen consumption in control and LS patient derived NPCs. MT-ATP6/PDH derived NPCs are deficient in OXPHOS energy generation compared to control. E. Extra cellular acidification rate (ECAR) for control and LS patient derived NPCs. F. Bioenergetic parameters for LS and control NPCs. MT-ATP6/PDH NPCs have reduced non-mitochondrial oxygen consumption (p=0.0354) and spare respiratory capacity when compared to control (p=0.0317). Graphs represent mean ± SEM from at least three independent experiments. ∗p<0.05; ∗∗p<0.01; ∗∗∗p<0.001; ∗∗∗∗p<0.0001.

Supplemental Figure 5

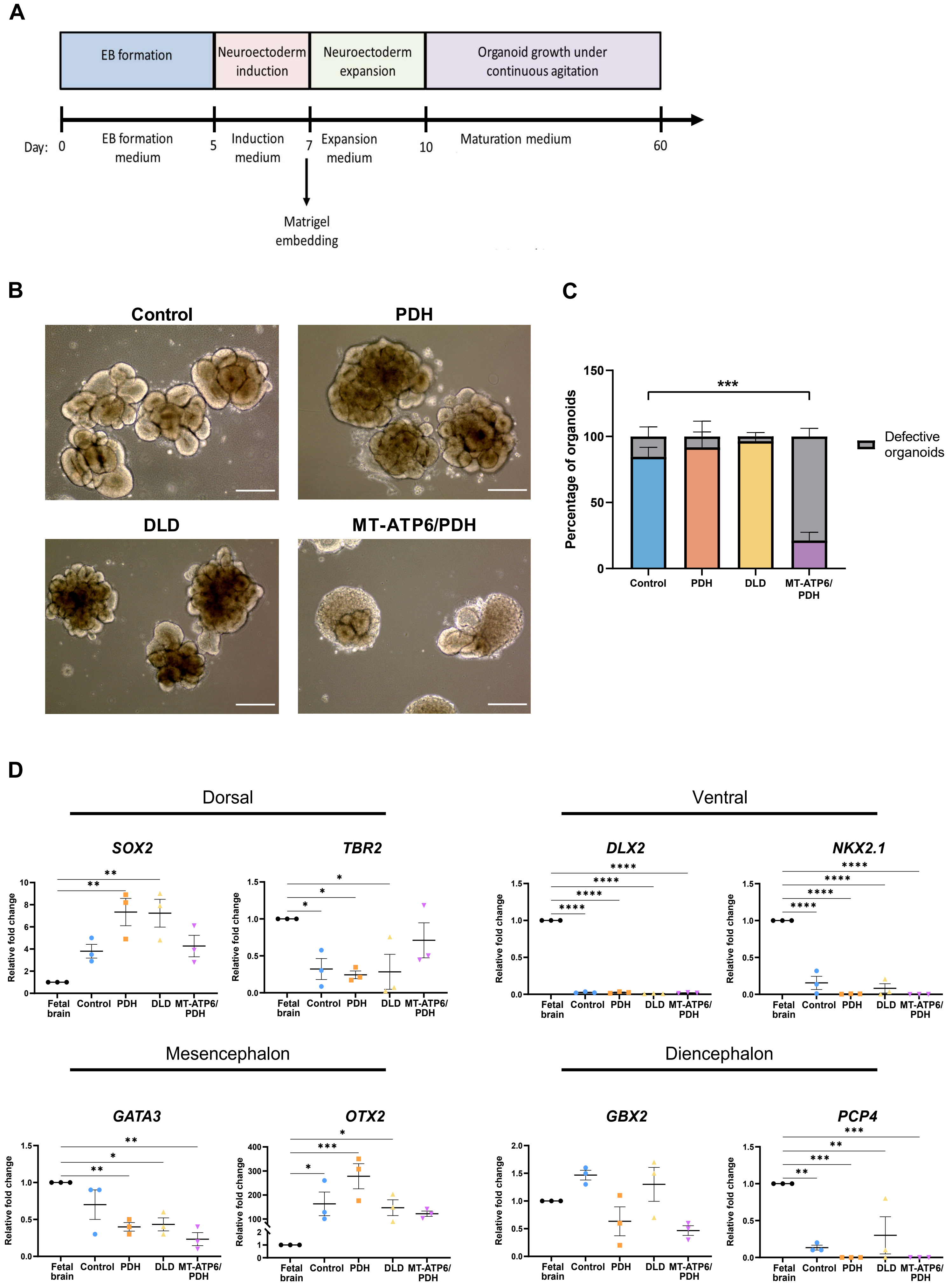

Development • Supplementary information

Fig. S5. MT-ATP6/PDH brain organoids display defective differentiation at day 10. A. Schematic of the brain organoid generation protocol. B. Brightfield images (4X) of day 10 brain organoids. MT-ATP6/PDH shows disorganized cellular growth that do not resemble neuroepithelial buds. Scale bar: 300µm. C. Quantification of the defective organoids at day 10 by cell line. D. Regional identity characterization of day 15 brain organoids. Forebrain (*SOX2* and *TBR2* for dorsal and *DLX2* and *NKX2.1* ventral telencephalon), diencephalon (*GBX2* and *PCP4*) and midbrain (mesencephalon, *GATA3* and *OTX2*) markers were assessed in all organoids and normalized to fetal brain RNA (20-33wks). *SOX2* expression was increased in all organoids compared to the fetal brain (PDH: p= 0.0026 and DLD: p= 0.0029). Expression of *TBR2* was lower in control organoids (p= 0.0479), PDH (p= 0.0275), and DLD (p= 0.0366). Expression of ventral markers *DLX2* and *NKX2.1* were expressed in very low values compared to fetal brain RNA (p<0.0001 in all cases). The early marker for mesencephalic fate *GATA3* was reduced in all three LS organoids (PDH: p= 0.0096, DLD: p= 0.0136, MT-ATP6/PDH p= 0.0019). *OTX2* expression was upregulated in the control (p= 0.0295), PDH (p= 0.0009) and DLD (p= 0.0494) organoids when compared to the fetal brain RNA expression. The diencephalon marker *GBX2* was expressed similarly among all genotypes, whereas *PCP4* was lower in all genotypes (control: p= 0.0010, PDH: p= 0.0003, DLD: p= 0.0048, MT-ATP6/PDH: p= 0.0003). Fold change normalized to GPI and GAPDH as house-keeping genes. Graphs represent mean ± SEM from three independent experiments. ∗p<0.05; ∗∗p<0.01; ∗∗∗p<0.001; ∗∗∗∗p<0.0001.

Supplemental Figure 6

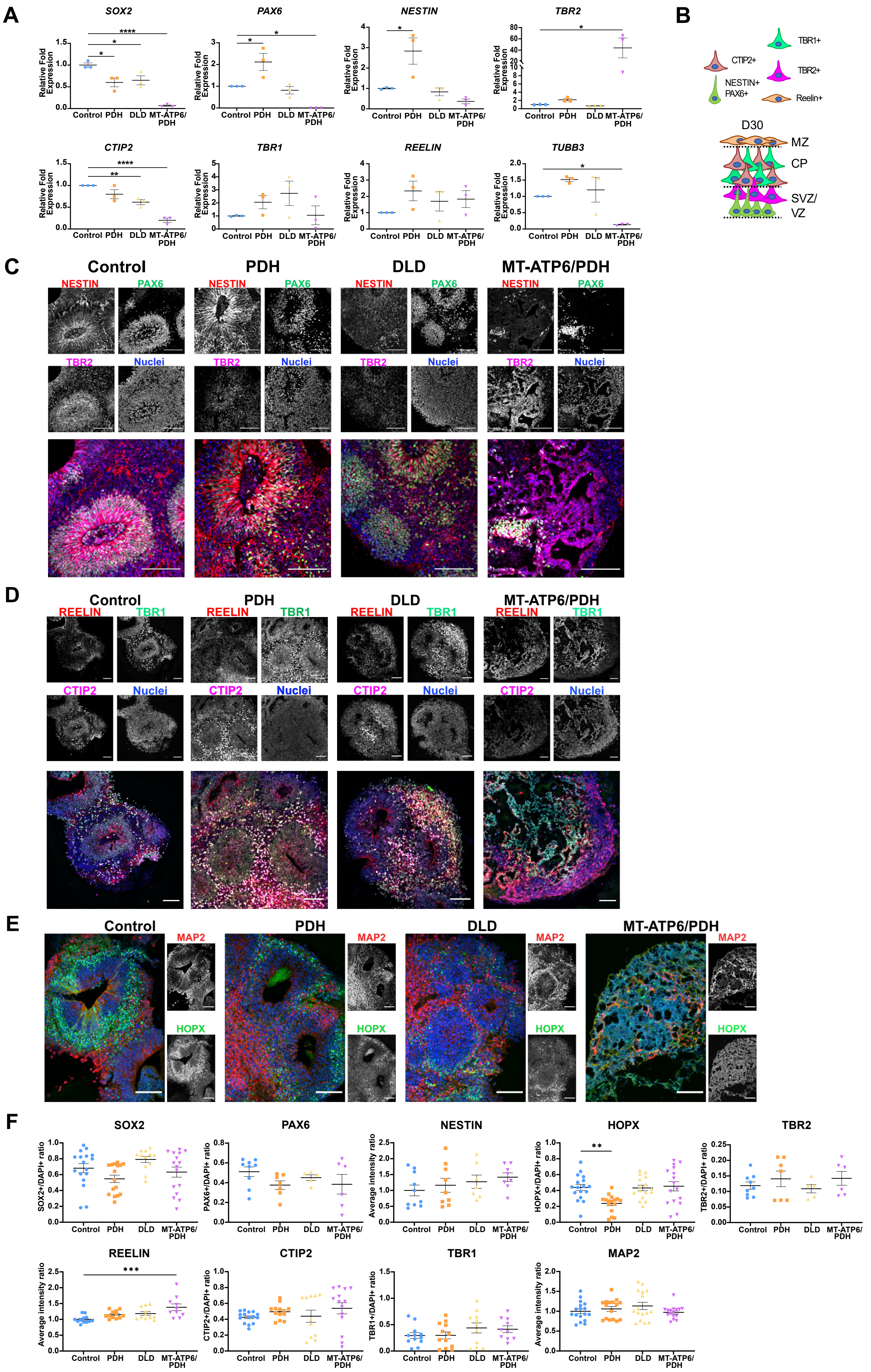

Fig. S6. Leigh syndrome brain organoids show defects in SVZ/VZ and CP formation. A. qPCR quantification of the day 30 organoids. Neural progenitor cell populations were evaluated by the expression of *SOX2*, *PAX6* and *NESTIN*. Intermediate progenitor cells were identified with the marker *TBR2*. Marginal zone marker *REELIN*, cortical plate markers *TBR1* and *CTIP2*, and neuronal marker *TUBB3* were also evaluated. Fold change normalized to GPI and GAPDH as house-keeping genes. B. Schematic representation of the expected organization of the brain organoids on day 30. C-E. Representative immunostaining confocal images of day 30 brain organoids. MT-ATP6/PDH mutant presents severe disorganization of the SVZ/VZ markers PAX6 and TBR2, as well as the neural progenitor marker NESTIN (C). Cajal-Retzius neurons positive for REELIN were observed in the surface of the organoids (D). Cortical plate markers CTIP2 and TBR1 (D), as well as outer radial glia marker HOPX and the neuronal marker MAP2 (E). For E, nuclei in merge image correspond to the blue channel. Scale bar: 100µm. Images were generated from at least three different organoids per genotype from 3 independent organoid batches. F. Quantification of day immunofluorescence staining for day 30 brain organoids. Outer radial glia marker HOPX was reduced in PDH organoids ($p=0.0032$) and MZ marker Reelin was increased ($p=0.0002$) in MT-ATP6/PDH mutant organoids. SVZ: subventricular zone, VZ: ventricular zone, CP: cortical plate, MZ: marginal zone. $*p<0.05$; $**p<0.01$; $***p<0.001$; $****p<0.0001$.

Development • Supplementary information

Supplemental Figure 7

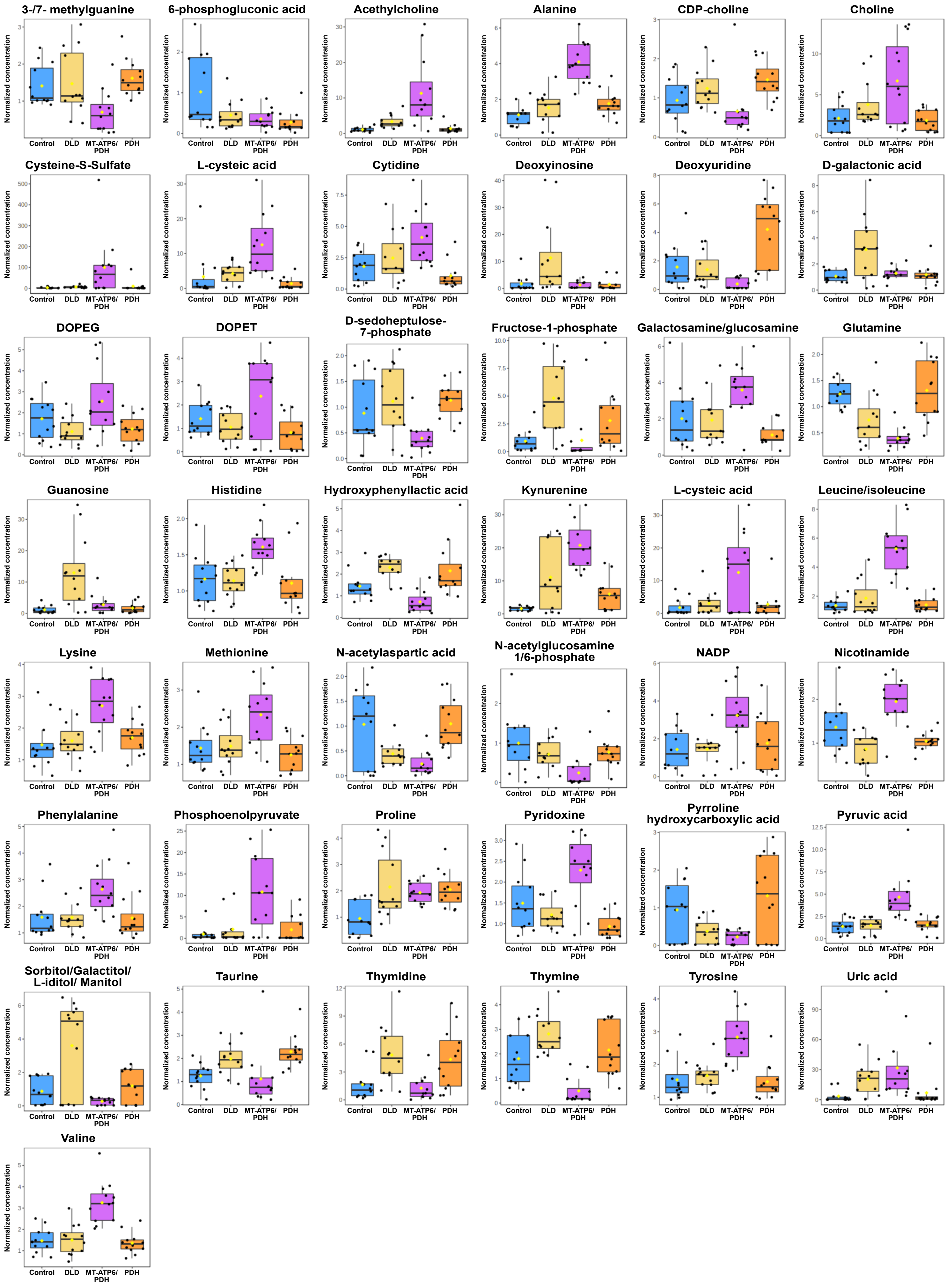

Development • Supplementary information

Fig. S7. Related to Fig. 7 and Table S2. Day 40 LS organoids show changes in their metabolic profiles. Individual graph bars for the 43 metabolites identified as statistically dysregulated ($p<0.05$ and FDR of 0.01) in LS Organoids when compared to control. Statistical values can be found in Supplemental Table 2. A total of three batches of 40-day organoids per line (4 independent organoids per line per batch) were analyzed as described in methods.

Development • Supplementary information

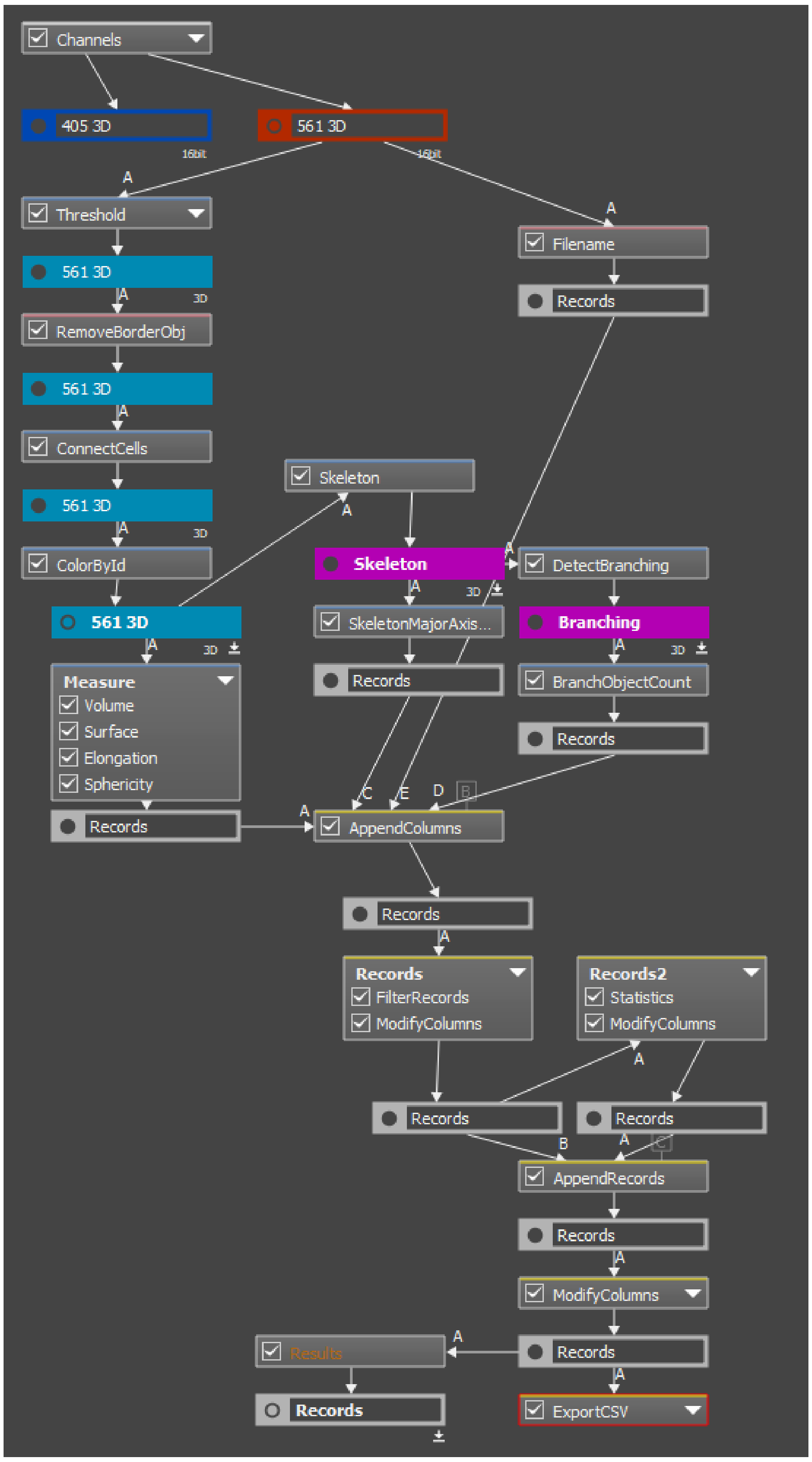

Fig. S8. Monolayer NPC mitochondrial quantification workflow.

Development • Supplementary information

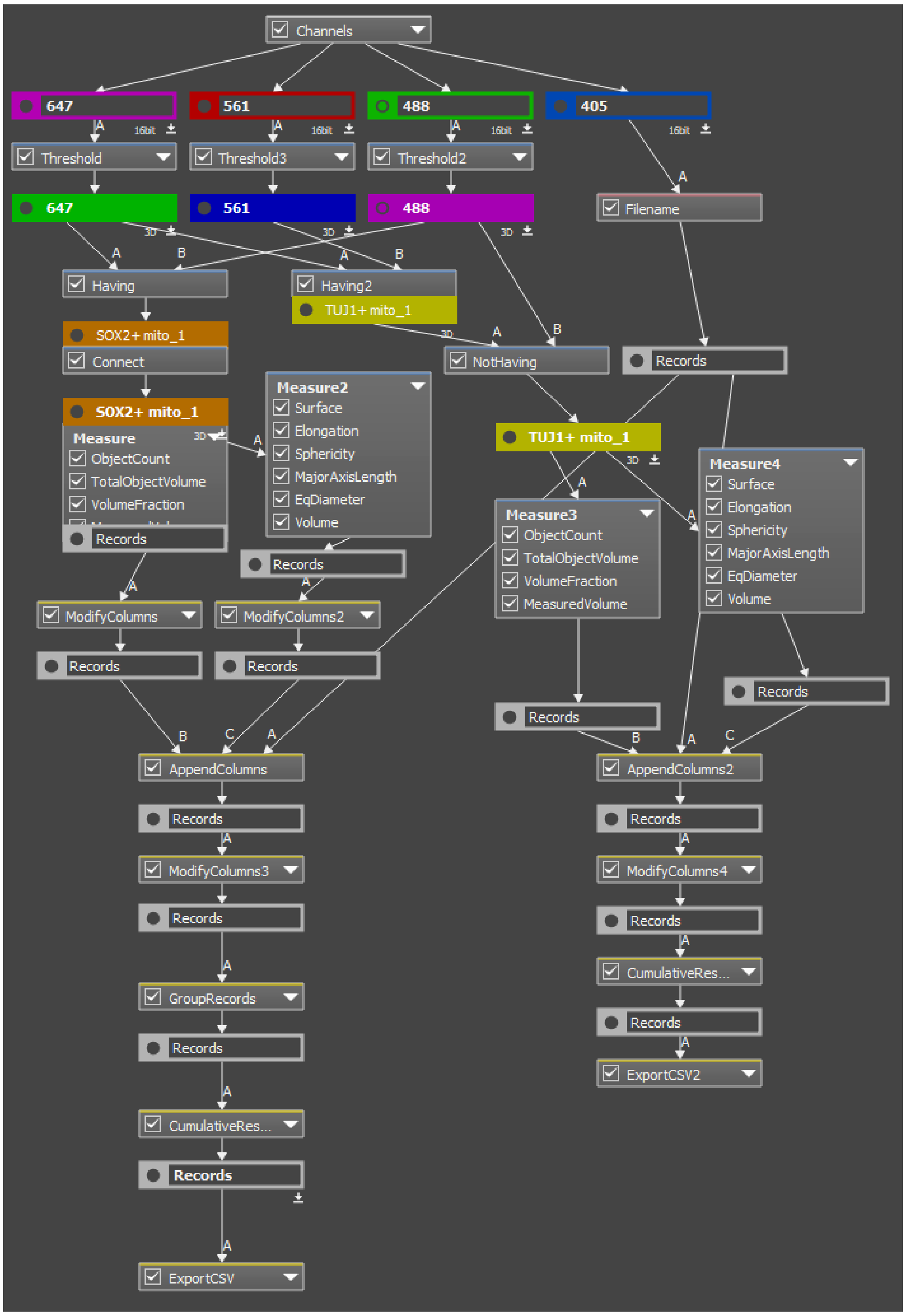

Fig. S9. Organoid NPC mitochondrial quantification workflow

Development • Supplementary information

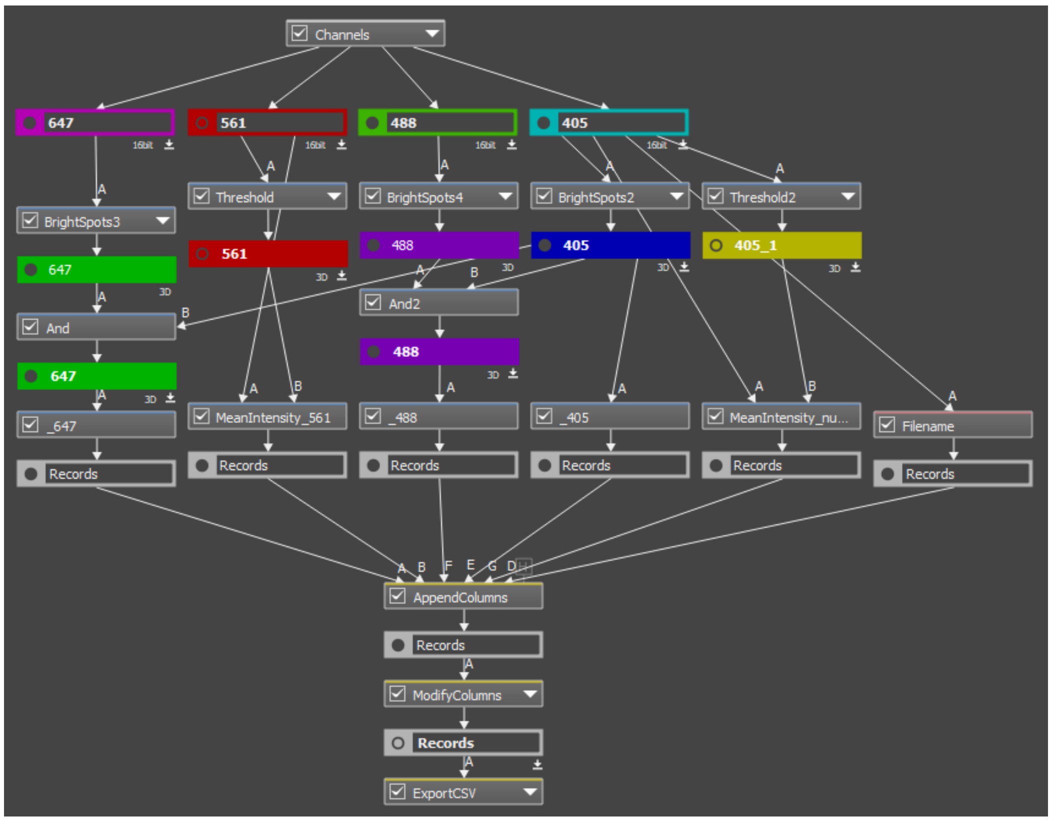

Fig. S10. Organoid quantification workflow for GA3, NIS Elements.

Table S1. Related to Fig. 1. Summary characteristics of the Leigh syndrome patient derived fibroblast cell lines including the patient phenotype at diagnosis, the mutations identified, and published literature using the cell line.

Cell line	Phenotype	Mutation	References
GM03672	1 y/o female, Caucasian. Developmental regression; microcephalic; elevated blood lactic acid and pyruvate; only affected family member	PDH (E1) LOF Pyruvate dehydrogenase (79delC, Arg27fs)	Hinman et al. 1989; Huh et al. 1990; Johnson et al. 2019
GM01503	3y/o female, Caucasian. Lactic acidosis, psychomotor delay; sister also affected. Subnormal activation of pyruvate dehydrogenase complex in disrupted fibroblasts	DLD (E3) Dihydrolipoamide dehydrogenase (A100G, Thr34Ala)	Cooper et al, 1969; Murphy JV, 1973; Sorbi& Blass, 1982; Hinman et al. 1989; Huh et al. 1990; Vo et al. 2007; Johnson et al. 2019
GM13411	8 month old male, Chinese. Lactic acidosis; developmental delay. Hypertrophic cardiomyopathy. Symmetric neural necrosis. Fibroblast: 90% heteroplasmy	MT-ATP6 (T8993G, Leu156Arg) PDH (E1) LOF Pyruvate dehydrogenase (79delC, Arg27fs)	Pastores et al. 1994; Iyer et al.2012; Ma et al. 2014; Galera-Monge et al. 2016; Zheng et al. 2016; Johnson et al. 2019

Table S2. Related to Fig. 7 and Fig. S7. Dysregulated metabolites in day 40 LS derived cerebral organoids. LC-MS measured metabolite peak areas were normalized to the total ion count (TIC) by sample and fold change was determined by dividing each LS TIC normalized peak area by the control TIC normalized peak area for each metabolite. One way ANOVA was utilized to identified metabolites that were significantly dysregulated (p<0.05). Post-hoc comparison column using Fisher's least significant difference method (Fisher's LSD) shows the comparisons between different levels that are significant given the p value threshold. Results shown are averages for 3 independent runs with 4 individual organoids per phenotype per run. FDR: False Discovery Rate.

Metabolite	f.value	p.value	-LOG10(p)	FDR	Fisher's LSD
leucine / leucineiso	32.463	3.17E-11	10.499	7.00E-09	MT-ATP6/PDH - Control; MT-ATP6/PDH - DLD; MT-ATP6/PDH - PDH
alanine	28.604	2.04E-10	9.6912	2.25E-08	MT-ATP6/PDH - Control; MT-ATP6/PDH - DLD; MT-ATP6/PDH - PDH
valine	18.839	5.25E-08	7.2801	3.87E-06	MT-ATP6/PDH - Control; MT-ATP6/PDH - DLD; MT-ATP6/PDH - PDH
kynurenine	17.496	1.27E-07	6.896	7.02E-06	DLD - Control; MT-ATP6/PDH - Control; MT-ATP6/PDH - DLD; MT-ATP6/PDH - PDH
thymine	13.799	1.77E-06	5.7523	7.82E-05	DLD - Control; Control - MT-ATP6/PDH; DLD - MT-ATP6/PDH; PDH - MT-ATP6/PDH
tyrosine	13.55	2.14E-06	5.6703	7.87E-05	MT-ATP6/PDH - Control; MT-ATP6/PDH - DLD; MT-ATP6/PDH - PDH
glutamine	13.161	2.88E-06	5.5408	9.09E-05	Control - DLD; Control - MT-ATP6/PDH; DLD - MT-ATP6/PDH; PDH - DLD; PDH - MT-ATP6/PDH
pyruvic acid	12.95	3.39E-06	5.4697	9.37E-05	MT-ATP6/PDH - Control; MT-ATP6/PDH - DLD; MT-ATP6/PDH - PDH
nicotinamide	12.723	4.05E-06	5.3925	9.95E-05	Control - DLD; MT-ATP6/PDH - Control; MT-ATP6/PDH - DLD; MT-ATP6/PDH - PDH
deoxyuridine	12.392	5.26E-06	5.2794	0.00011615	PDH - Control; PDH - DLD; PDH - MT-ATP6/PDH
pyridoxine	11.7	9.16E-06	5.0381	0.00018405	MT-ATP6/PDH - Control; Control - PDH; MT-ATP6/PDH - DLD; MT-ATP6/PDH - PDH
sorbitol / galactitol / L-iditol / manitol	11.289	1.28E-05	4.8919	0.00023619	DLD - Control; DLD - MT-ATP6/PDH; DLD - PDH
acetylcholine	10.88	1.80E-05	4.7444	0.00030625	MT-ATP6/PDH - Control; MT-ATP6/PDH - DLD; MT-ATP6/PDH - PDH
guanosine	10.42	2.66E-05	4.5755	0.00041955	DLD - Control; DLD - MT-ATP6/PDH; DLD - PDH
hydroxyphenyllactic acid	10.302	2.94E-05	4.5318	0.00043306	DLD - Control; Control - MT-ATP6/PDH; PDH - Control; DLD - MT-ATP6/PDH; PDH - MT-ATP6/PDH
phosphoenolpyruvate	9.3744	6.59E-05	4.1812	0.00091016	MT-ATP6/PDH - Control; MT-ATP6/PDH - DLD; MT-ATP6/PDH - PDH
cystine	8.3901	0.00016027	3.7951	0.0020835	MT-ATP6/PDH - Control; MT-ATP6/PDH - DLD; MT-ATP6/PDH - PDH
N-acetylaspartic acid	8.239	0.00018425	3.7346	0.0022622	Control - DLD; Control - MT-ATP6/PDH; PDH - DLD; PDH - MT-ATP6/PDH
lysine	7.7264	0.00029759	3.5264	0.0034614	MT-ATP6/PDH - Control; MT-ATP6/PDH - DLD; MT-ATP6/PDH - PDH
methionine	6.9562	0.00062316	3.2054	0.0067872	MT-ATP6/PDH - Control; MT-ATP6/PDH - DLD; MT-ATP6/PDH - PDH
galactosamine / glucosamine	6.921	0.00064493	3.1905	0.0067872	MT-ATP6/PDH - Control; MT-ATP6/PDH - DLD; MT-ATP6/PDH - PDH
cytidine	6.8316	0.00070382	3.1525	0.0070702	MT-ATP6/PDH - Control; MT-ATP6/PDH - DLD; MT-ATP6/PDH - PDH
histidine	6.6085	0.00087664	3.0572	0.0084224	MT-ATP6/PDH - Control; MT-ATP6/PDH - DLD; MT-ATP6/PDH - PDH
L-cysteic acid	6.5655	0.00091465	3.0387	0.0084224	MT-ATP6/PDH - Control; MT-ATP6/PDH - DLD; MT-ATP6/PDH - PDH
pyrroline hydroxycarboxylic acid	6.3042	0.0011864	2.9258	0.010293	Control - MT-ATP6/PDH; PDH - DLD; PDH - MT-ATP6/PDH
D-galactonic acid	6.2562	0.0012448	2.9049	0.010293	DLD - Control; DLD - MT-ATP6/PDH; DLD - PDH
thymidine	6.2461	0.0012575	2.9005	0.010293	DLD - Control; PDH - Control; DLD - MT-ATP6/PDH; PDH - MT-ATP6/PDH
choline	6.1696	0.0013579	2.8671	0.010718	MT-ATP6/PDH - Control; MT-ATP6/PDH - DLD; MT-ATP6/PDH - PDH
F1P	6.0835	0.001481	2.8295	0.011286	DLD - Control; DLD - MT-ATP6/PDH
proline	5.6016	0.0024208	2.616	0.017833	DLD - Control; MT-ATP6/PDH - Control; PDH - Control
taurine	5.3747	0.0030613	2.5141	0.021824	DLD - Control; PDH - Control; DLD - MT-ATP6/PDH; PDH - MT-ATP6/PDH
6-phosphogluconic acid	5.202	0.0036652	2.4359	0.025313	Control - DLD; Control - MT-ATP6/PDH; Control - PDH
NADP	5.1267	0.0039663	2.4016	0.026562	MT-ATP6/PDH - Control; MT-ATP6/PDH - DLD; MT-ATP6/PDH - PDH
uric acid	4.9978	0.0045425	2.3427	0.027934	DLD - Control; MT-ATP6/PDH - Control; MT-ATP6/PDH - PDH
phenylalanine	4.9917	0.0045714	2.3399	0.027934	MT-ATP6/PDH - Control; MT-ATP6/PDH - DLD; MT-ATP6/PDH - PDH
deoxyinosine	4.981	0.0046237	2.335	0.027934	DLD - Control; DLD - MT-ATP6/PDH; DLD - PDH
D-sedoheptulose-7-phosphate	4.9702	0.0046767	2.3301	0.027934	Control - MT-ATP6/PDH; DLD - MT-ATP6/PDH; PDH - MT-ATP6/PDH
DOPET	4.9279	0.0048903	2.3107	0.028441	MT-ATP6/PDH - Control; MT-ATP6/PDH - DLD; MT-ATP6/PDH - PDH
cysteine-S-sulfate	4.8891	0.0050957	2.2928	0.028876	MT-ATP6/PDH - Control; MT-ATP6/PDH - DLD; MT-ATP6/PDH - PDH
3- / 7-methylguanine	4.8504	0.0053089	2.275	0.029331	Control - MT-ATP6/PDH; DLD - MT-ATP6/PDH; PDH - MT-ATP6/PDH
N-acetylglucosamine 1/6-phosphate	4.8231	0.0054652	2.2624	0.029459	Control - MT-ATP6/PDH; DLD - MT-ATP6/PDH; PDH - MT-ATP6/PDH
DOPEG	4.4575	0.0080819	2.0925	0.042526	MT-ATP6/PDH - DLD; MT-ATP6/PDH - PDH
CDP-choline	4.3847	0.0087424	2.0584	0.044932	PDH - Control; DLD - MT-ATP6/PDH; PDH - MT-ATP6/PDH

Development • Supplementary information

Table S3. Related to Related to Fig. 7 and Fig. S7. Summary of the metabolic pathways analysis for metabolites enriched in day 40 PDH brain organoids. Statistical p values from enrichment analysis are adjusted for multiple hypothesis testing. Total: total number of compounds in the pathway. Hits: matched number from the uploaded data. Raw p: original p value calculated from the enrichment analysis. Holm p: p value adjusted by Holm-Bonferroni method. FDR p: adjusted p value using False Discovery Rate. Impact: pathway impact value calculated from pathway topology analysis.

Metabolite	Total	Expected	Hits	Raw p	-LOG10(p)	Holm adjust	FDR	Impact
Pyrimidine metabolism	39	0.20129	2	0.0157	1.8041	1	1	0.1172
Taurine and hypotaurine metabolis	8	0.04129	1	0.0406	1.391	1	1	0.4286
Vitamin B6 metabolism	9	0.046452	1	0.0456	1.3408	1	1	0.0784
Pentose phosphate pathway	22	0.11355	1	0.1083	0.96538	1	1	0.1196
Glycerophospholipid metabolism	36	0.18581	1	0.1717	0.76513	1	1	0.0193
Arginine and proline metabolism	38	0.19613	1	0.1805	0.74359	1	1	0.0778
Primary bile acid biosynthesis	46	0.23742	1	0.2146	0.66836	1	1	0.0076
Aminoacyl-tRNA biosynthesis	48	0.24774	1	0.2229	0.65181	1	1	0

m

Table S4. Related to Related to Fig. 7 and Fig. S7. Summary of the metabolic pathways analysis for metabolites enriched in day 40 DLD brain organoids. Statistical p values from enrichment analysis are adjusted for multiple hypothesis testing. Total: total number of compounds in the pathway. Hits: matched number from the uploaded data. Raw p: original p value calculated from the enrichment analysis. Holm p: p value adjusted by Holm-Bonferroni method. FDR p: adjusted p value using False Discovery Rate. Impact: pathway impact value calculated from pathway topology analysis.

Metabolite	Total	Expected	Hits	Raw p	-LOG10(p)	Holm adjust	FDR	Impact
Purine metabolism	65	0.62903	4	0.00271	2.5662	0.22805	0.228	0.0059
Pyrimidine metabolism	39	0.37742	3	0.00544	2.2645	0.45142	0.228	0.0971
Alanine, aspartate and glutamate metabol	28	0.27097	2	0.02858	1.5439	1	0.8	0.2003
D-Glutamine and D-glutamate metabolism	6	0.058065	1	0.05677	1.2459	1	0.916	0
Nitrogen metabolism	6	0.058065	1	0.05677	1.2459	1	0.916	0
Taurine and hypotaurine metabolism	8	0.077419	1	0.07501	1.1249	1	0.916	0.4286
Aminoacyl-tRNA biosynthesis	48	0.46452	2	0.07632	1.1174	1	0.916	0
Arginine biosynthesis	14	0.13548	1	0.12779	0.89352	1	1	0
Nicotinate and nicotinamide metabolism	15	0.14516	1	0.1363	0.86549	1	1	0.1943
Fructose and mannose metabolism	20	0.19355	1	0.17774	0.75021	1	1	0.0304
Pentose phosphate pathway	22	0.2129	1	0.19379	0.71267	1	1	0.1196
Glyoxylate and dicarboxylate metabolism	32	0.30968	1	0.26974	0.56905	1	1	0
Arginine and proline metabolism	38	0.36774	1	0.31205	0.50578	1	1	0.0778
Tryptophan metabolism	41	0.39677	1	0.33233	0.47842	1	1	0.0942
Primary bile acid biosynthesis	46	0.44516	1	0.36491	0.43782	1	1	0.0076

Development • Supplementary information

Table S5. Related to Related to Fig. 7 and Fig. S7. Summary of the metabolic pathways analysis for metabolites enriched in day 40 MT-ATP6/PDH brain organoids. Statistical p values from enrichment analysis are adjusted for multiple hypothesis testing. Total: total number of compounds in the pathway. Hits: matched number from the uploaded data. Raw p: original p value calculated from the enrichment analysis. Holm p: p value adjusted by Holm-Bonferroni method. FDR p: adjusted p value using False Discovery Rate. Impact: pathway impact value calculated from pathway topology analysis.

Metabolite	Total	Expected	Hits	Raw p	-LOG10(p)	Holm adjust	FDR	Impact
Aminoacyl-tRNA biosynthesis	48	0.71226	6	4.35E-05	4.3617	0.0036523	0.0036523	0
Alanine, aspartate and glutamate metabolism	28	0.41548	4	0.00059684	3.2241	0.049538	0.025067	0.20032
Cysteine and methionine metabolism	33	0.48968	3	0.011653	1.9336	0.95556	0.32629	0.10446
Arginine and proline metabolism	38	0.56387	3	0.017166	1.7653	1	0.33051	0.13058
Nicotinate and nicotinamide metabolism	15	0.22258	2	0.019673	1.7061	1	0.33051	0.1943
Citrate cycle (TCA cycle)	20	0.29677	2	0.034034	1.4681	1	0.47648	0.04634
Pyruvate metabolism	22	0.32645	2	0.040643	1.391	1	0.48771	0.20684
Glycolysis / Gluconeogenesis	26	0.38581	2	0.055171	1.2583	1	0.54228	0.20594
Phenylalanine, tyrosine and tryptophan biosynthesis	4	0.059355	1	0.058102	1.2358	1	0.54228	0.5
Glyoxylate and dicarboxylate metabolism	32	0.47484	2	0.079815	1.0979	1	0.55522	0
Glycine, serine and threonine metabolism	33	0.48968	2	0.084213	1.0746	1	0.55522	0
D-Glutamine and D-glutamate metabolism	6	0.089032	1	0.085928	1.0659	1	0.55522	0
Nitrogen metabolism	6	0.089032	1	0.085928	1.0659	1	0.55522	0
Glycerophospholipid metabolism	36	0.53419	2	0.097841	1.0095	1	0.5848	0.02582
Pyrimidine metabolism	39	0.57871	2	0.11206	0.95053	1	0.5848	0.03727
Taurine and hypotaurine metabolism	8	0.11871	1	0.11297	0.94705	1	0.5848	0
Ubiquinone and other terpenoid-quinone biosynthesis	9	0.13355	1	0.1262	0.89895	1	0.5848	0
Tyrosine metabolism	42	0.62323	2	0.12681	0.89685	1	0.5848	0.13972
Phenylalanine metabolism	10	0.14839	1	0.13924	0.85624	1	0.5848	0
Biotin metabolism	10	0.14839	1	0.13924	0.85624	1	0.5848	0
Arginine biosynthesis	14	0.20774	1	0.18957	0.72223	1	0.75827	0
Purine metabolism	65	0.96452	2	0.25062	0.60098	1	0.9489	0
Selenocompound metabolism	20	0.29677	1	0.25982	0.58533	1	0.9489	0
Pentose phosphate pathway	22	0.32645	1	0.28191	0.54989	1	0.98669	0.11955
Lysine degradation	25	0.37097	1	0.31387	0.50324	1	1	0
Glutathione metabolism	28	0.41548	1	0.34447	0.46285	1	1	0.0018
Tryptophan metabolism	41	0.60839	1	0.46262	0.33477	1	1	0.09417

Development • Supplementary information

Table S6. Key Resource Table

REAGENT or RESOURCE	SOURCE	IDENTIFIER
**ANTIBODIES**		
**Primary Antibodies (Immunocytochemistry)**		
Mouse anti-MAP2 (1:100)	Thermo Fisher Scientific	Cat # 131500, AB_2533001
Rabbit anti-GFAP (1:200)	Agilent Technologies	Cat # Z0334, AB_10013382
Rabbit anti-HOPX (1:2500)	Sigma-Aldrich	Cat # HPA030180, AB_10603770
Mouse anti-mitochondria (1:200)	Abcam	Cat # ab92824, AB_10562769
Rabbit anti-SOX2 (1:200)	Cell Signaling Technology	Cat # 5049S, AB_10828386
Rabbit anti-PAX6 (1:200)	Cell Signaling Technology	Cat # 60433, AB_2797599
Mouse anti-β3 TUBULIN (1:100)	Cell Signaling Technology	Cat # 4466, AB_10270973
Rabbit anti-S100 (1:300)	Abcam	Cat # ab868, AB_306716
Mouse anti-Olig2 (1:500)	Millipore Sigma	Cat # MABN50, AB_10807410
Rat anti-α TUBULIN (1:500)	Thermo Fisher Scientific	Cat # MA180017, AB_2210201
Mouse anti-ZO-1 (1:500)	Thermo Fisher Scientific	Cat # 339100, AB_2533147
Rabbit anti-CDK5RAP2 (1:500)	Bethyl Laboratories	Cat # IHC00063, AB_2076863
Mouse anti-NESTIN (1:100)	STEMCELL Technologies	Cat # 60091, AB_2650581
Chicken anti-TBR2 (1:100)	Millipore Sigma	Cat # AB15894, AB_10615604
Mouse anti-REELIN (1:200)	Millipore Sigma	Cat # MAB5366, AB_2285132
Rat anti-CTIP2 (1:200)	Abcam	Cat # ab18465, AB_2064130
Rabbit anti-TBR1 (1:200)	Abcam	Cat # ab31940, AB_2200219
Rabbit anti-TOM20 (1:200)	Cell Signaling Technology	Cat # 42406, AB_2687663
Mouse anti-SATB2 (1:100)	Abcam	Cat # ab51502, AB_882455
Mouse anti-BRN2 (POU3F2) (1:100)	Millipore Sigma	Cat # MABD51, AB_11204531
Mouse anti-CASP (CUX1) (1:100)	Abcam	Cat # ab54583, AB_941209
Rabbit anti-ALDH1L1 (1:50)	Cell Signaling Technology	Cat # 85828S
**Secondary Antibodies (Immunocytochemistry)**		
Goat anti Chicken Alexa Fluor 647 (1:500)	Thermo Fisher Scientific	Cat # A-21449, AB_2535866
Goat anti Rat Alexa Fluor 647 (1:500)	Thermo Fisher Scientific	Cat # A-21247, AB_2535864
Donkey anti Rabbit Alexa Fluor 647 (1:500)	Thermo Fisher Scientific	Cat # A-31573, AB_2536183
Donkey anti Mouse Alexa Fluor 647 (1:500)	Thermo Fisher Scientific	Cat # A-31571, AB_162542
Donkey anti Rabbit Alexa Fluor 546 (1:500)	Thermo Fisher Scientific	Cat # A-10040, AB_2534016
Donkey anti Mouse Alexa Fluor 546 (1:500)	Thermo Fisher Scientific	Cat # A-10036, AB_2534012
Donkey anti Rabbit Alexa Fluor 488 (1:500)	Thermo Fisher Scientific	Cat # A-21206, AB_2535792
Donkey anti Mouse Alexa Fluor 488 (1:500)	Thermo Fisher Scientific	Cat # A-21202, AB_141607

Development • Supplementary information

**Primary Antibodies (Western Blotting)**		
Rabbit anti-PAX6 (1:300)	Cell Signaling Technology	Cat # 60433, AB_2797599
Mouse anti-NESTIN (1:500)	STEMCELL Technologies	Cat # 60091, AB_2650581
Rabbit anti-Sox2 (1:1000)	Cell Signaling Technology	Cat # 3579, AB_2195767
Mouse anti- α TUBULIN (1:2000)	Sigma-Aldrich	Cat # T9026, AB_477593
**Secondary Antibodies -HRP conjugated (Western Blotting)**		
Peroxidase AffiniPure Donkey Anti-Rabbit IgG (H+L) (1:5000)	Jackson ImmunoResearch Inc	Cat # 711-035-152, AB_10015282
Peroxidase AffiniPure Donkey Anti-Mouse IgG (H+L) (1:5000)	Jackson ImmunoResearch Inc	Cat # 715-035-151, AB_2340771
**CHEMICALS, PEPTIDE, AND RECOMBINANT PROTEINS**		
Y-27632 Rho/Rock pathway inhibitor	STEMCELL Technologies	Cat # 72307
Dorsomorphin	Millipore Sigma	Cat # P5499
SB431542	REPROCELL	Cat # 04-0010-10
Etoposide	Millipore Sigma	Cat # E1383
Carbonyl cyanide 3-chlorophenylhydrazone (CCCP)	Sigma Aldrich	Cat # C2759
Nocodazole	Sigma Aldrich	Cat # M1404
Neocarzinostatin	Sigma Aldrich	Cat # 9162
**CRITICAL COMMERCIAL ASSAYS AND KITS**		
PluriTest Assay	Thermo Fisher Scientific	Cat# A38154
KaryoStat Assay	Thermo Fisher Scientific	Cat# A38153
Mitochondrial DNA sequencing	Girihlet	
Whole Exome sequencing	Genewiz LLC	
CytoTune iPS 2.0 Sendai Reprogramming Kit	Thermo Fisher Scientific	Cat # A16517
STEMdiff Trilineage Differentiation Kit	STEMCELL Technologies	Cat# 05230
STEMdiff™ SMADi Neural Induction medium	STEMCELL Technologies	Cat# 08581
NeuroCult™ media	STEMCELL Technologies	Cat # 05752
Astrocyte medium	ScienCell	Cat # 1801
STEMdiff™ Cerebral Organoid Kit	STEMCELL Technologies	Cat # 08570
STEMdiff™ Cerebral Organoid Maturation Kit	STEMCELL Technologies	Cat # 08571
Seahorse Cell Mito Stress Test	Agilent	Cat # 103015-100
Seahorse XF DMEM medium pH 7.4	Agilent	Cat#103575-100
Seahorse XF 1.0 M glucose solution	Agilent	Cat#103577-100

Development • Supplementary information

Seahorse XF 100mM pyruvate solution	Agilent	Cat#103578-100
Seahorse XF 200 mM glutamine solution	Agilent	Cat#103579-100
Seahorse XF calibrant	Agilent	Cat#100840-000
Seahorse XF96 V3 PS cell culture microplates	Agilent	Cat#101085-004
CellTiter Blue Viability Assay	Promega	Cat # G8081
**DEPOSITED DATA**		
Raw and analyzed sequencing data	Done by Creative Solutions (J.P.C, Vanderbilt University	https://www.ncbi.nlm.nih.gov/sra/PRJNA626388<br>https://vandydata.github.io/Romero-Morales-Gama-Leigh-Syndrome-WES/
**EXPERIMENTAL MODELS: CELL LINES**		
AG16409 control fibroblasts – analyzed for contamination	Coriell Institute	https://www.coriell.org/0/Sections/Search/Sample_Detail.aspx?Ref=AG16409&Product=CC
GM13411 (MT-ATP6/PDH) fibroblasts – analyzed for contamination	Coriell Institute	https://www.coriell.org/0/Sections/Search/Sample_Detail.aspx?Ref=GM13411&Product=CC
GM03672 (PDH Mutant) fibroblasts – analyzed for contamination	Coriell Institute	https://www.coriell.org/0/Sections/Search/Sample_Detail.aspx?Ref=GM03672&Product=CC
GM01503 (DLD Mutant) fibroblasts – analyzed for contamination	Coriell Institute	https://www.coriell.org/0/Sections/Search/Sample_Detail.aspx?Ref=GM01503&Product=CC
**SEQUENCE-BASED REAGENTS**		
**Primers for Trilineage assay**		
POU5F1	Integrated DNA Technologies	Forward GGGCTCTCCCATGCATTCAAAC<br>Reverse CACCTTCCCTCCAACCAGTTGC
NANOG	Integrated DNA Technologies	Forward TGGGATTTACAGGCGTGAGCCAC<br>Reverse AAGCAAAGCCTCCCAATCCCAAAC
GAPDH	Integrated DNA Technologies	Harvard Primer Bank, ID: 378404907c2<br>Forward ACAACTTTGGTATCGTGGAAGG<br>Reverse GCCATCACGCCACAGTTTC
GPI	Integrated DNA Technologies	Forward GTGTACCTTCTAGTCCCGCC<br>Reverse GGTCAAGCTGAAGTGGTTGAAGC
GATA3	Integrated DNA Technologies	Forward TGGAGGAGGAATGCCAATGGG<br>Reverse GCCGGGTTAAACGAGCTGTTCTTG

Development • Supplementary information

NESTIN	Integrated DNA Technologies	Harvard Primer Bank, ID: 38176299c1<br>Forward CTGCTACCCTTGAGACACCTG<br>Reverse GGGCTCTGATCTCTGCATCTAC
PAX6	Integrated DNA Technologies	Harvard Primer Bank, ID: 189083679c1<br>Forward TGGGCAGGTATTACGAGACTG<br>Reverse ACTCCCGCTTATACTGGGCTA
CDX2	Integrated DNA Technologies	Forward CTGGAGCTGGAGAAGGAGTTTCAC<br>Reverse GACACTTCTCAGAGGACCTGGCTG
SOX17	Integrated DNA Technologies	Harvard Primer Bank, ID:145275218c1<br>Forward GTGGACCGCACGGAATTTG<br>Reverse GGAGATTCACACCGGAGTCA
TBXT	Integrated DNA Technologies	Forward ACAATGCCAGCCCACCTACCAG<br>Reverse CGTACTGGCTGTCCACGATGTCTG
NCAM	Integrated DNA Technologies	Harvard Primer Bank, ID:316659209c1<br>Forward GGGGTTGCTTGTCAGTAGC<br>Reverse TTCAGGTTCACCAATCGCTGT
**Primers for Multipotency**		
S100B	Integrated DNA Technologies	Harvard Primer Bank, ID: 114520588c1<br>Forward TGGCCCTCATCGACGTTTTC<br>Reverse ATGTTCAAAGAACTCGTGGCA
GFAP	Integrated DNA Technologies	Reference: (Marton et al., 2019)<br>Forward GGCAAAAGCACCAAAGACGG<br>Reverse GGCGGCGTTCCATTTACAAT
O4 (FOXO4)	Integrated DNA Technologies	Harvard Primer Bank, ID: 283436081c1<br>Forward GGCTGCCGCGATCATAGAC<br>Reverse GGCTGGTTAGCGATCTCTGG
OLIG2	Integrated DNA Technologies	Reference: (Marton et al., 2019)<br>Forward AAGGCAGTTGCTGTGGAAAC<br>Reverse GCAAACAGCTTAGCATTGCG
TUBB3	Integrated DNA Technologies	Harvard Primer Bank, ID: 308235961c1<br>Forward GGCCAAGGGTCACTACACG<br>Reverse GCAGTCGCAGTTTTCACACTC
MAP2	Integrated DNA Technologies	Harvard Primer Bank, ID: 87578393c1<br>Forward CTCAGCACCGCTAACAGAGG<br>Reverse CATTGGCGCTTCGGACAAG
**Primers for Brain Organoids**		
Reelin	Integrated DNA Technologies	Harvard Primer Bank, ID: 223718142c2<br>Forward ACATCTACAAGTGTTCAGGCATC<br>Reverse TGGTTACCAAACTGGTGGTCA
HOPX	Integrated DNA Technologies	Harvard Primer Bank, ID: 21311737a1<br>Forward GAGACCCAGGGTAGTGATTTGA<br>Reverse AAAAGTAATCGAAAGCCAAGCAC
CTIP2 (BCL11B)	Integrated DNA Technologies	Harvard Primer Bank, ID: 12597634c2<br>Forward TCCAGCTACATTTGCACAACA<br>Reverse GCTCCAGGTAGATGCGGAAG

Development • Supplementary information

TBR2 (EOMES)	Integrated DNA Technologies	Harvard Primer Bank, ID: 22538469c2<br>Forward GTGCCCACGTCTACCTGTG<br>Reverse CCTGCCCTGTTTCGTAATGAT
TBR1	Integrated DNA Technologies	Harvard Primer Bank, ID: 22547231c1<br>Forward GCAGCAGCTACCCACATTCA<br>Reverse AGGTTGTCAGTGGTCGAGATA
SOX2	Integrated DNA Technologies	Forward CCATGCAGGTTGACACCGTTG<br>Reverse TCGGCAGACTGATTCAAATAATACAG
SATB2	Integrated DNA Technologies	Harvard Primer Bank, ID: 289547595c2<br>Forward GACAGTGGCCGACATGCTAC<br>Reverse AGGCAAGTCTTCCAACTTTGAA
BRN2 (POU3F2)	Integrated DNA Technologies	Harvard Primer Bank, ID: 380254475c1<br>Forward CGGCGGATCAAACTGGGATTT<br>Reverse TTGCGCTGCGATCTTGTCTAT
CUX1	Integrated DNA Technologies	Harvard Primer Bank, ID: 321400113c1<br>Forward GAAGAACCAAGCCGAAACCAT<br>Reverse AGGCTCTGAACCTTATGCTCA
VIMENTIN	Integrated DNA Technologies	Harvard Primer Bank, ID: 240849334c2<br>Forward AGTCCACTGAGTACCGGAGAC<br>Reverse CATTTCACGCATCTGGCGTTC
SOX9	Integrated DNA Technologies	Harvard Primer Bank, ID: 182765453c1<br>Forward AGCGAACGCACATCAAGAC<br>Reverse CTGTAGGCGATCTGTTGGGG
ALDH1L1	Integrated DNA Technologies	Harvard Primer Bank, ID: 21614512c3<br>Forward TCCAGACCTTCCGCTACTTTG<br>Reverse CAGGGGATAGTTCCAGGGGAT
**Housekeeping primers**		
GAPDH	Integrated DNA Technologies	Harvard Primer Bank, ID: 378404907c2<br>Forward ACAACTTTGGTATCGTGGAAGG<br>Reverse GCCATCACGCCACAGTTTC
GPI (F1/R1)	Integrated DNA Technologies	Forward GTGTACCTTCTAGTCCCGCC<br>Reverse GGTCAAGCTGAAGTGGTTGAAGC
**SOFTWARE AND ALGORITHMS**		
Image Studio™ Lite	LI-COR	https://www.licor.com/bio/image-studio-lite/download
Fiji	Schindelin et al., 2012	https://imagej.net/Fiji
GraphPad Prism v8.1.2	GraphPad	https://www.graphpad.com/scientific-software/prism/
NIS-Elements	Nikon Instruments	https://www.microscope.healthcare.nikon.com/products/software/nis-elements

Development • Supplementary information

MetaboAnalyst 5.0	(Chong and Xia, 2018; Chong et al., 2018; Chong et al., 2019; Xia and Wishart, 2010; Xia and Wishart, 2011a; Xia and Wishart, 2011b; Xia et al., 2009)	https://www.metaboanalyst.ca/home.xhtml
SnpSift	(Cingolani et al., 2012)	http://snpeff.sourceforge.net/SnpSift.html
R 3.5.3	R Foundation	https://www.r-project.org/
BioCircos	(Cui et al., 2016)	https://cran.r-project.org/web/packages/BioCircos/index.html
Seahorse Wave	Agilent	https://www.agilent.com/en/product/cell-analysis/real-time-cell-metabolic-analysis/xf-software/seahorse-wave-desktop-software-740897
**OTHER**		
Mitotracker Red CMXRos	Fisher Scientific	Cat # M7512
Matrigel™	Corning	Cat # 354277
Gentle dissociation solution	STEMCELL Technologies	Cat # 07174
Seahorse XFe96 Analyzer	Agilent	N/A
Aggrewell™ 800 24-well plate	STEMCELL Technologies	Cat # 34815
AggreWell™ Rinsing Solution	STEMCELL Technologies	Cat # 07010
Human Fetal Brain Total RNA	Takara Bio	Cat # 636526

Development • Supplementary information
