# Supplementary material for: Human iPSC-derived cerebral organoids model features of Leigh syndrome and reveal abnormal corticogenesis
Source: Development. 2022 Jul 6;149(20):dev199914. doi: 10.1242/dev.199914 (PMC9357378; doi:10.1242/dev.199914)
Supplement: Reviewer comments [file develop-149-199914_review_history.pdf]

Human iPSC-derived cerebral organoids model features of Leigh syndrome and reveal abnormal corticogenesis

Alejandra I. Romero-Morales, Gabriella L. Robertson, Anuj Rastogi, Megan L. Rasmussen, Hoor Temuri, Gregory Scott McElroy, Ram Prosad Chakrabarty, Lawrence Hsu, Paula M. Almonacid, Bryan A. Millis, Navdeep S. Chandel, Jean-Philippe Cartailler and Vivian Gama
DOI: 10.1242/dev.199914

Editor: James Briscoe

Review timeline

Original submission:	17 June 2021
Editorial decision:	18 August 2021
First revision received:	30 November 2021
Editorial decision:	21 January 2022
Second revision received:	6 May 2022
Accepted:	18 May 2022

Original submission

First decision letter

MS ID#: DEVELOP/2021/199914

MS TITLE: Human iPSC-derived cerebral organoids model features of Leigh Syndrome and reveal abnormal corticogenesis

AUTHORS: Alejandra I. Romero-Morales, Gabriella L. Robertson, Anuj Rastogi, Megan L. Rasmussen, Hoor Temuri, Gregory S. McElroy, Ram P. Chakrabarty, Lawrence Hsu, Paula M. Almonacid, Bryan A. Millis, Navdeep Chandel, Jean-Philippe Cartailler, and Vivian Gama

I am sorry that it took longer than expected to give you an answer. I have been tracking down the reviews and have just gotten them. The referees' comments are appended below, or you can access them online: please go to BenchPress and click on the 'Manuscripts with Decisions' queue in the Author Area.

As you will see, the referees express considerable interest in your work, but have some significant criticisms and recommend a substantial revision of your manuscript before we can consider publication. If you are able to revise the manuscript along the lines suggested, which may involve further experiments, I will be happy receive a revised version of the manuscript. Your revised paper will be re-reviewed by one or more of the original referees, and acceptance of your manuscript will depend on your addressing satisfactorily the reviewers' major concerns. Please also note that Development will normally permit only one round of major revision.

We are aware that you may be experiencing disruption to the normal running of your lab that make experimental revisions challenging. If it would be helpful, we encourage you to contact us to discuss your revision in greater detail. Please send us a point-by-point response indicating where you are able to address concerns raised (either experimentally or by changes to the text) and where you will not be able to do so within the normal timeframe of a revision. We will then provide further guidance. Please also note that we are happy to extend revision timeframes as necessary.

© 2022. Published by The Company of Biologists under the terms of the Creative Commons Attribution License (https://creativecommons.org/licenses/by/4.0/).

Please attend to all of the reviewers' comments and ensure that you clearly highlight all changes made in the revised manuscript. Please avoid using 'Tracked changes' in Word files as these are lost in PDF conversion. I should be grateful if you would also provide a point-by-point response detailing how you have dealt with the points raised by the reviewers in the 'Response to Reviewers' box. If you do not agree with any of their criticisms or suggestions please explain clearly why this is so.

Reviewer 1

Advance summary and potential significance to field

This manuscript details the use of organoids derived from human samples as a model system to study the effects of mitochondrial disease mutations on brain development, which is otherwise not possible in humans.
This study is very interesting and highly relevant to the field. The use of organoids has recently gained much attention and has become a very useful tool for the understanding of human brain development and the effects of mutations in human patients on neural development and brain function. Furthermore, knowledge acquired in recent years has indicated the importance of mitochondria and mitochondrial morphology in neural stem cells and cortical development in mouse models, however, there remains limitations as to whether this may also be true in human brain development and human neurogenesis. This study, using LS as a model, paves the way for the understanding of mitochondrial dysfunction in the context of human brain physiology.

Comments for the author

The reviewer finds that this study is potentially suitable for publication in Development, however, revisions towards presentation of the data are suggested. Furthermore, the written text of this manuscript is difficult to understand and needs some major revisions in terms of clearly defining the results and interpretations. Many experiments are not clearly explained and statements of the results are incomplete and often very difficult to follow. In addition, some figures presented with associated quantification, at times, don't appear to match whereby the images clearly shows differences between mutant and control, however the quantification does not reflect that.
-In figure 2, authors state that "no significant differences were detected among the different lines" however the images do not seem to reflect that. One example is Sox17 for PDH. Is there truly no difference or is it the low sample number (n=3) with one clear outlier that is resulting in "no significant difference". The authors should be very careful about such claims, since there may in fact be a difference if proper sample size was acquired. In that case, one cannot publish that there is no difference.
-Same issues with figure 3, quantifications claim no difference however the images are not very reflective of that. Furthermore, it is not clear as to why the quantifications in this figure are done by measuring fluorescent intensity rather the cell number. This can be very misleading and accounts only for expression levels.
-Figure 5, Panel A shows decreased gene expression of many important markers. Is this due to actual changes in gene expression or simply a lower number of cells that express those markers? Along the same lines, if all these markers are low what cells are occupying all the DAPI seen. ie is it simply less expression of these markers or less cells that are defined by these markers?
-As indicated by the authors, mitochondrial visualization has not been done in human developing brains. This presents a very novel finding that should be made available in the main figures rather than being in the supplemental figures.
-it is not clear how mitochondrial length was measured. In particular, if the mutant ATP6/PDH has aggregated mitochondria this would pose a problem with the mitochondria length data and may lead to an overestimation of mitochondrial elongation in this scenario.
-The scales for mitochondrial measurements may not be correct. For example, in supp figure 5B the diameter of mitochondria is indicated as "um" and shows it to be on average about 3um in diameter. This would be a very thick mitochondria. Another example, the mitochondrial length graph shows the axis as a measurement scale of "mm".
-on the subject of mitochondrial morphology, the results indicate clear mitochondrial structural changes in the different mutants. The authors should discuss this important finding and present possible explanations as to how this may be contributing to the phenotype.

© 2022. Published by The Company of Biologists under the terms of the Creative Commons Attribution License (https://creativecommons.org/licenses/by/4.0/).

-the authors should explain the discrepancy surrounding their observation that 2D neurogenesis is not affected, meanwhile the organoid system shows many defects.
-authors say they use "NPC" and "progenitor" markers. However, NPC is defined as neural progenitor/precursor cells. Thus the distinction is not clear. If the authors are using NPC to define neural stem cells than that should be made clear.

Reviewer 2

Advance summary and potential significance to field

In the manuscript entitled “Human iPSC-derived cerebral organoids model features of Leigh Syndrome and reveal abnormal corticogenesis” Romero-Morales et al. work with 4 commercially available fibroblast cell lines to model Leigh Syndrome. The authors report defects in neuroepithelium differentiation in 3D cortical organoids and cortical architecture. Strikingly the authors report a disturbed balance between neurogenesis and astrogliogenesis accompanied by abnormal mitochondrial morphologies. While I appreciate the need for human models to study diseases like Leigh Syndrom I do have major concerns with the conclusions presented as outlined below.

Comments for the author

Major:
Proper controls should go far beyond a single age-matched control fibroblast line in particular since different iPSC, even healthy control iPSC lines, show dramatically distinct propensities to differentiate into one or the other brain region (Kanton et al. Nature 2019). Gene correction in the experimental iPSC lines or introduction of the patient-specific mutations in control iPSC lines is absolutely essential to substantiate the claims raised by the body of data, at least where possible. As a minimum for the nuclear encoded genes this should be possible, while I do understand the technical difficulties to do this for mitochondrial encoded genes.
Moreover, given the Kanton et al. 2019 paper, the authors should include at least a battery of qPCR analysis to determine the regional identities of the organoids generated.
PDH patient shows microcephaly, and this is nicely recapitulated by the organoid system. However, also the other LS-patient derived organoids show decreased organoid size while these patients seem not to suffer from microcephaly. How can this be interpreted? And I guess also here this data demands more specific controls.
The increase in astrocytic markers on protein level (p180ff, Fig3) while no decrease in NPC, neuronal or oligodenroglial markers, as well as no significant mRNA deregulation of astrocytic markers, rather hints towards an astrogliosis and not towards a disturbed differentiation. To claim an effect on glial differentiation this has to be substantiated with a experiments such as BrdU birthdating experiment (e.g. 48h BrdU treatment how many BrdU+ cells express astrocytic markers, and is that changed between the conditions), or extensive clonal analysis, or live-imaging combined with endpoint analysis (following single NPCs and their progeny and define the fate at the end of the movie by fixing, and staining (e.g. SOX2, TUBB3, S100B))
The experimental data show a bigger EB diameter, what does this mean, or could this mean. The authors should either comment on this in the discussion or leave it out? The same is true for lumen size. This is a very interesting aspect but what is the biological significance of this? This should be discussed?
MT-ATP6/PDH organoids show more defective organoids. In the phenotypic description in supplemental Figure 6A,C these organoids show less to no expression of all assessed markers except TBR2. At the same time the TBR2 staining is not convincing, in particular in MT-ATP6/PDH as it shows strong non-nuclear labelling. These organoids seem to show almost no neuropeithalial organization. The tissue architecture looks like it could be choroid plexus. I think it would be worthwhile to check on RNA level for TTR expression.
The pictures shown in Supplemental Figure 6 C,D,E don’t support the quantification shown in Supplemental Figure 6F for MT-ATP6/PDH. E.g. there is barely a MAP2 positive cell in the picture in suppFig6E, but the quantification shows no change. Either these pictures are not representative, but they go very well along with the qPCR data, or and that seems more likely the quantification in suppFig6e is flawed.

© 2022. Published by The Company of Biologists under the terms of the Creative Commons Attribution License (https://creativecommons.org/licenses/by/4.0/).

Moreover, having a specific difference in the levels of HOPX, but not in PAX6, SOX2 or the resulting cell types, TBR1, CTIP2, MAP2 at d30 is hard to explain (more HOPX at the expense of which other cell type(s)?
Increased self-renewal of oRGs?)
Cerebral organoids are not a suitable system to study a syndrome which is described as “Focal, bilateral lesions in one or more CNS regions including brainstem, thalamus, basal ganglia, cerebellum and spinal cord” (line 72-73). As referred to by the introduction of the manuscript, LS is not affecting the cerebral cortex. Is this correct? And if so, how can a system recapitulating early human cerebral cortex development serve as a model? Or in other words what is the significance of the presented data for the disease etiology? The way the manuscript is presented does not explain this to the reader.

Minor.
The abbreviation for Leigh syndrome, as defined in the manuscript, is LS (line 53). Any tissue, organoid or cell cannot be LS-derived. That would mean that this cell, tissue, organoid is derived from a syndrome, which evidently is never the case, they are much rather derived from LS-patients.
The number of neural rosettes per field of view is changed if the ventricular lumen is changed. To accurately determine the number of neural rosettes, these structures should be quantified per section and not per field of view.
In my opinion iPSC reprogramming and differentiation potential of these cells should go into supplementary data, since it does not add much to the core message of the paper.
p.value in graphs. Either give real numbers not > or <, or categorize by *, **, ***. Having actual numbers combined with < is consistent as <0.0001 could also be put as ***.
The intro of the discussion is unnecessarily long and unspecific, taking away space for more specific aspects which in the current manuscript are not discussed.

First revision

<u>Author response to reviewers' comments</u>

We are thankful to the reviewers of Development for their comments and suggestions to our manuscript. The insightful reviews significantly improved our manuscript. Below you can find a detailed response to all reviewers’ comments.

Reviewer 1 Advance summary and potential significance to field
This manuscript details the use of organoids derived from human samples as a model system to study the effects of mitochondrial disease mutations on brain development, which is otherwise not possible in humans. This study is very interesting and highly relevant to the field. The use of organoids has recently gained much attention and has become a very useful tool for the understanding of human brain development and the effects of mutations in human patients on neural development and brain function. Furthermore, knowledge acquired in recent years has indicated the importance of mitochondria and mitochondrial morphology in neural stem cells and cortical development in mouse models, however, there remains limitations as to whether this may also be true in human brain development and human neurogenesis. This study, using LS as a model, paves the way for the understanding of mitochondrial dysfunction in the context of human brain physiology.

Reviewer 1 Comments for the author
The reviewer finds that this study is potentially suitable for publication in Development, however, revisions towards presentation of the data are suggested. Furthermore, the written text of this manuscript is difficult to understand and needs some major revisions in terms of clearly defining the results and interpretations. Many experiments are not clearly explained and statements of the results are incomplete and often very difficult to follow. In addition, some figures presented with associated quantification, at times, don't appear to match, whereby the images clearly shows differences between mutant and control, however the quantification does not reflect that.

© 2022. Published by The Company of Biologists under the terms of the Creative Commons Attribution License (https://creativecommons.org/licenses/by/4.0/).

We thank Reviewer 1 for the insightful comments. We have made major changes to data quantification, interpretation, and written text to increase clarity of results and interpretations. We hope that these changes are now appropriate for our manuscript to be published in Development.

1) In Figure 2, authors state that "no significant differences were detected among the different lines" however the images do not seem to reflect that. One example is Sox17 for PDH. Is there truly no difference or is it the low sample number (n=3) with one clear outlier that is resulting in "no significant difference". The authors should be very careful about such claims, since there may in fact be a difference if proper sample size was acquired. In that case, one cannot publish that there is no difference.

We agree with Reviewer 1 that the low sample number could be a confounding factor with these data. We repeated the trilineage differentiation experiment with new clones to validate these results and increase the sample number in order to increase the statistical analysis power. Although all cell lines can generate three germ layer markers using our current protocol (Joshi et al., 2020; Kuang et al., 2019; Ortolano et al., 2021; Roberts et al., 2019), we observed an inherent variability in the differentiation efficiency among clones. This variability can be due to several factors: 1) the time of differentiation is short in the protocol we used, 2) variability from having different genetic backgrounds, 3) heteroplasmy or potential X-linked gene silencing (Juchniewicz et al., 2021; Lissens et al., 2000; Migeon, 2020). In order to convey our results at a population level and to avoid unintentional bias, we removed the immunofluorescence images and increased the number of data points in the RT qPCR analysis (see new Supplementary figure 2). Variation in the differentiation potential of the cell lines is intriguing and should be examined in future experiments with fetal tissues of the appropriate lineages as positive controls. The effects of the LS mutations in other highly metabolic tissues, such as muscle, kidney, and liver, should be examined to have a complete picture of this devastating disease. Unfortunately, additional lines with these mutations are not available at public resources. We believe that since these lines are commercially available and widely used in research studies, a detailed characterization is useful to the field. A high throughput analysis of several LS patients-derived lines is needed to have a clear understanding of the impact of mitochondrial mutations on these tissues. Our laboratory is keen in adapting the CRISPR technology to engineer single point mutations in mitochondrial genes in a control background to avoid genomic variations between patients for future studies. However, this effort will take significant time.

2) Same issues with figure 3, quantifications claim no difference however the images are not very reflective of that. Furthermore, it is not clear as to why the quantifications in this figure are done by measuring fluorescent intensity rather the cell number. This can be very misleading and accounts only for expression levels.

Reviewer 1 brings up a good point. We quantify the data in Figure 3 by cell number for the nuclear makers PAX6 and OLIG2. We observed a slight increase in the PAX6+/DAPI+ nuclei ratio for the PDH cell line ($p=0.0494$). Figure 2A and figure legend were updated to reflect this new finding.
We maintained the fluorescent intensity measurement for cytoplasmatic markers as demarcating individual cells without a membrane marker would be challenging. The total fluorescent intensity was normalized to the DAPI intensity in each case to account for cellular density.

3) Figure 5, Panel A shows decreased gene expression of many important markers. Is this due to actual changes in gene expression or simply a lower number of cells that express those markers? Along the same lines, if all these markers are low what cells are occupying all the DAPI seen. ie is it simply less expression of these markers or less cells that are defined by these markers?

The reviewer raises an important point. Based on immunofluorescence analyses, it does not seem the number of cells found at the cortical-like areas is decreased. These cells appear to express low levels of these markers both at the gene and protein level. Whether this is simply less expression of the markers or less cells defined by the markers is fascinating and worth examining further. This could be done in future studies by single cell mass cytometry and scRNAseq which could help us identify the identity of these cells. We include this point in the discussion section of the paper.

© 2022. Published by The Company of Biologists under the terms of the Creative Commons Attribution License (https://creativecommons.org/licenses/by/4.0/).

4) As indicated by the authors, mitochondrial visualization has not been done in human developing brains. This presents a very novel finding that should be made available in the main figures rather than being in the supplemental figures.

We agree with this point. This figure is now included as a main figure of the manuscript.

5) it is not clear how mitochondrial length was measured. In particular, if the mutant ATP6/PDH has aggregated mitochondria this would pose a problem with the mitochondria length data and may lead to an overestimation of mitochondrial elongation in this scenario.

We apologize for the oversight. During paper preparation, we omitted to include the detailed method of mitochondrial morphology estimation we have developed and optimized in our laboratory. We now include a detailed description of the methods section as well as a diagram of the workflow that was utilized for the mitochondrial quantification. Briefly, quantification of mitochondrial morphology was performed by segmenting mitochondria in 3D and performed skeletonization of the resulting 3D mask. Skeleton major axis and sphericity measurements were exported into Excel, and the data was filtered and analyzed in PRISM. We updated the discussion to include the caveats of our mitochondrial quantification method.

6) The scales for mitochondrial measurements may not be correct. For example, in supp figure 5B the diameter of mitochondria is indicated as "um" and shows it to be on average about 3um in diameter. This would be a very thick mitochondria. Another example, the mitochondrial length graph shows the axis as a measurement scale of "mm".

We appreciate this comment. One of the limitations of our quantification analysis is a reduced ability to segment individual mitochondria. It is hard to threshold and segment the mitochondria near the lumen, since there are so many, and the cells are very close to each other. The upper values in our measurements can be attributed to large, interconnected areas of mitochondria that the threshold can't distinguish one from another rather than swollen or thick individual mitochondrion. We updated the discussion to describe the caveats of measuring mitochondrial parameters in organoids.
Regarding the mitochondrial graph axis, we apologize for the mistake. All measurements were performed in μm. The manuscript and figures have been corrected to reflect the correct scales.

7) on the subject of mitochondrial morphology, the results indicate clear mitochondrial structural changes in the different mutants. The authors should discuss this important finding and present possible explanations as to how this may be contributing to the phenotype.

We thank the reviewer for this suggestion. We extended our discussion on these findings as requested and also included new bioenergetics data at the iPSC and NPC stages (New Supplemental Figure 4).

8) the authors should explain the discrepancy surrounding their observation that 2D neurogenesis is not affected, meanwhile the organoid system shows many defects.

We agree with this point. We extended the discussion on these findings as requested.

9) authors say they use "NPC" and "progenitor" markers. However, NPC is defined as neural progenitor/precursor cells. Thus, the distinction is not clear. If the authors are using NPC to define neural stem cells than that should be made clear.

We revised the manuscript to clarify that the populations referred to as NPCs are a mixed population of neural progenitor and precursor cells. We hope that this change clarifies that we are looking at both cells with different neural lineage capacity and stages.

Reviewer 2 Advance summary and potential significance to field In the manuscript entitled “Human iPSC-derived cerebral organoids model features of Leigh Syndrome and reveal abnormal corticogenesis” Romero-Morales et al. work with 4 commercially available fibroblast cell lines to model Leigh Syndrome. The authors report defects in neuroepithelium differentiation in 3D cortical organoids and cortical architecture. Strikingly the authors report a disturbed balance between

© 2022. Published by The Company of Biologists under the terms of the Creative Commons Attribution License (https://creativecommons.org/licenses/by/4.0/).

neurogenesis and astrogliogenesis accompanied by abnormal mitochondrial morphologies. While I appreciate the need for human models to study diseases like Leigh Syndrome, I do have major concerns with the conclusions presented as outlined below.

Reviewer 2 Comments for the author Major: Proper controls should go far beyond a single age-matched control fibroblast line in particular since different iPSC, even healthy control iPSC lines, show dramatically distinct propensities to differentiate into one or the other brain region (Kanton et al. Nature 2019).

10) Gene correction in the experimental iPSC lines or introduction of the patient-specific mutations in control iPSC lines is absolutely essential to substantiate the claims raised by the body of data, at least where possible. As a minimum for the nuclear encoded genes this should be possible, while I do understand the technical difficulties to do this for mitochondrial encoded genes.

Reviewer 2 brings up an important point. Isogenic lines indeed provide a homogenous genetic background. However, as the reviewer points out generating mutations in the mitochondrial genome is not trivial, with the first report just recently published in the literature (Mok et al., 2020). To address the reviewer's concern, we attempted to generate a PDHαE1 knockout iPSC line in the well characterized male line GM25256 (Coriell Institute). Immediately after CRISPR, we detect a slight reduction of ~50% in the protein expression of PDHαE1 in the KO pool compared to no sgRNA control (Response to reviewers Figure 1). Unfortunately, clone isolation did not result in KO clones, confirming the lethal phenotype. After several passages, the KO population in the pool was lost and the PDHαE1 expression was restored to control levels. Previous reports in mouse models have shown that global PDHαE1 deletions are embryonic lethal for both sexes and in male mosaic animals (Johnson et al., 2001; Pliss et al., 2013). Conditional KO of PDHαE1 in the brain (Nestin Cre) also show marked dysregulation in neurogenesis, maturation, and migration (Pliss et al., 2004). Global DLD KO is also lethal in mouse models by 7.5 dpc (Johnson et al., 1997). Unfortunately, introducing the single mutations in a wildtype background has proven to be technically challenging. As stated previously, since these lines are the only ones that are commercially available at the moment, a detailed characterization will be useful to the field. Leigh syndrome has been considered an early-onset neurodegenerative disease where the neurons develop normally and then start degenerating because of free radical damage. Our study and the study with SURF mutations of our colleague Alessandro Prigione (Inak et al., 2021) show that there is an additional neurodevelopmental component to this disease. We hope that the reviewer would agree that performing genome engineering experiments would be extremely labor and cost-intensive.
To control for other mutations and genetic alterations, we reviewed the WES and mitochondrial data and included the annotation impact of the high and moderate impact variants shared between samples in both INDELS and SNPs (Supplemental Figure S1 A & B). We also included the top 15 high impact SNPs variants (Supplemental Figure S1C). Moreover, a link to a data repository with the raw and analyzed WES and mitochondrial results is included in the manuscript as a tool for researchers in the field.

11) Moreover, given the Kanton et al. 2019 paper, the authors should include at least a battery of qPCR analysis to determine the regional identities of the organoids generated.

We agree with the reviewer. The Kanton study is a major advance for the field and while qPCR analysis would be informative, we worry that the results could be misleading due to the heterogeneity of the samples used in this study. As suggested, by reviewer 2, we are planning to introduce these LS-associated mutations in a control background and generate organoids from these iPSCs to precisely identify the effects on the mutations on various regional identities. Alejandra Romero-Morales (first author of this study) has also adapted the protocols to generate dorsal and ventral organoids, a system that would be invaluable to discern this interesting aspect of brain development and the impact of mitochondrial dysregulation on brain regional identity.

PDH patient shows microcephaly, and this is nicely recapitulated by the organoid system. However, also the other LS-patient derived organoids show decreased organoid size while these patients seem not to suffer from microcephaly. How can this be interpreted? And I guess also here this data demands more specific controls.

© 2022. Published by The Company of Biologists under the terms of the Creative Commons Attribution License (https://creativecommons.org/licenses/by/4.0/).

Our findings imply that there might be an exhaustion of the NPC pool over time. This would explain the lack of organized progenitor growth and impaired neuronal differentiation, as well as the overall reduced size of the organoids. However, additional experiments are needed to make these conclusions. Reviewer 1 is correct that microcephaly was not reported in patients with other mutations. Considering the concerns of Reviewer 1 and 2, we will remove this data from the paper.

12) The increase in astrocytic markers on protein level (p180ff, Fig3) while no decrease in NPC, neuronal or oligodenroglial markers, as well as no significant mRNA deregulation of astrocytic markers, rather hints towards an astrogliosis and not towards a disturbed differentiation. To claim an effect on glial differentiation this has to be substantiated with a experiments such as BrdU birthdating experiment (e.g. 48h BrdU treatment how many BrdU+ cells express astrocytic markers, and is that changed between the conditions), or extensive clonal analysis, or live-imaging combined with endpoint analysis (following single NPCs and their progeny and define the fate at the end of the movie by fixing, and staining (e.g. SOX2, TUBB3, S100B))

The speculation of gliosis is intriguing, but we agreed that it would require a new suite of experiments to conclusively demonstrate it. We will describe this possibility in the discussion of the paper and present it as a potential direction of this study.

13) The experimental data show a bigger EB diameter, what does this mean, or could this mean. The authors should either comment on this in the discussion or leave it out? The same is true for lumen size. This is a very interesting aspect but what is the biological significance of this? This should be discussed? MT-ATP6/PDH organoids show more defective organoids. In the phenotypic description in supplemental

Considering the general concerns presented by reviewer 2 regarding the EB and organoid size estimation, we decided to remove these data from the paper.

14) Figure 6A,C these organoids show less to no expression of all assessed markers except TBR2. At the same time the TBR2 staining is not convincing, in particular in MT-ATP6/PDH as it shows strong non-nuclear labelling.

Regarding the mRNA expression of TBR2, we modified the graph to include a break in the X axis to better portrait the expression of this marker in the Control, PDH and DLD. Because of the high expression in the double mutant, the previous scale failed to show the expression in the other genotypes.

In the case of the TBR2 staining, we agree that there is non-nuclear labelling. The antibody that we used (Millipore Sigma, cat # AB15894) has been cited in multiple human brain and brain organoid/spheroid papers (Albert et al., 2017; Bershteyn et al., 2017; Camp et al., 2015; Florio et al., 2016; Johnson et al., 2015; Joshi et al., 2020; Lancaster et al., 2017; Micali et al., 2020; Renner et al., 2017). To specifically avoid overestimating the TBR2+ cells, we quantified the nuclei where the TBR2 staining overlapped with DAPI in 3D. We had included more details of the quantification in the Methods section.

15) These organoids seem to show almost no neuroepithelial organization. The tissue architecture looks like it could be choroid plexus. I think it would be worthwhile to check on RNA level for TTR expression.

This an intriguing observation that overlaps with our response to point 11. We are keen in following these suggestions in future studies with gene-edited cells.

16) The pictures shown in Supplemental Figure 6 C,D,E don't support the quantification shown in Supplemental Figure 6F for MT-ATP6/PDH. E.g. there is barely a MAP2 positive cell in the picture in suppFig6E, but the quantification shows no change. Either these pictures are not representative, but they go very well along with the qPCR data, or and that seems more likely the quantification in suppFig6e is flawed.

We thank the reviewer for this comment. We reviewed and reanalyzed our immunofluorescent (IF) images. We updated the graphs and text as needed. We have observed that while the IF

© 2022. Published by The Company of Biologists under the terms of the Creative Commons Attribution License (https://creativecommons.org/licenses/by/4.0/).

quantification trends similarly to the RT -qPCR data it doesn't always recapitulate it. Differences may be due to the fact that RT-qPCR data is generated using whole organoids and the IF is focused on regions of interest. We have revised all IF representative images.

17) Moreover, having a specific difference in the levels of HOPX, but not in PAX6, SOX2 or the resulting cell types, TBR1, CTIP2, MAP2 at d30 is hard to explain (more HOPX at the expense of which other cell type(s)? Increased self-renewal of oRGs?)

At day 30 we do not observe an increase in HOPX. Our first quantification showed reduced percentage of HOPX+ cells in PDH and MT-ATP6/PDH organoids, while our current quantification shows reduction only in PDH organoids. This reduction may be associated to a slight, yet not significant, increase in the percentage of neurons (MAP2, CTIP and Reelin). But more studies are needed to get to this specific point more accurately.

18) Cerebral organoids are not a suitable system to study a syndrome which is described as "Focal, bilateral lesions in one or more CNS regions including brainstem, thalamus, basal ganglia, cerebellum and spinal cord" (line 72-73). As referred to by the introduction of the manuscript, LS is not affecting the cerebral cortex. Is this correct? And if so, how can a system recapitulating early human cerebral cortex development serve as a model? Or in other words what is the significance of the presented data for the disease etiology? The way the manuscript is presented does not explain this to the reader.

The lesions that have been associated with LS can appear in different regions of the CNS, including the cerebral cortex (Alves et al., 2020; Lake et al., 2016; Sofou et al., 2018). Imaging studies have showed 21% of the patients had cerebral cortical lesions and 100% of the cerebral cortical involvement was statistically associated with mtDNA etiology (Alves et al., 2020). This highlights the advantage of using cerebral organoids for the study of LS. We have updated the manuscript to convey this point.

Leigh syndrome has been considered an early-onset neurodegenerative disease where the neurons develop normally and then start degenerating because of free radical damage. Our study and the study with SURF mutations of our colleague Alessandro Prigione (Inak et al., 2021) show that there is an additional neurodevelopmental component to this disease.

Minor.

19) The abbreviation for Leigh syndrome, as defined in the manuscript, is LS (line 53). Any tissue, organoid or cell cannot be LS-derived. That would mean that this cell, tissue, organoid is derived from a syndrome, which evidently is never the case, they are much rather derived from LS-patients.

We agree with this point. We updated the manuscript to reflect the changes.

20) The number of neural rosettes per field of view is changed if the ventricular lumen is changed. To accurately determine the number of neural rosettes, these structures should be quantified per section and not per field of view.

As stated in the Method section, the NR quantification was done using images acquired at 10X magnification, this to ensure the inclusion of all or most of the EB in the ROI. Representative figures in the main figure are 20X images that were taken to better appreciate the differences in morphology. We have included examples of the 10X images in the Response to Reviewers Figure 2 to assure that we are considering most of the EB in the quantification and had included the differences in magnification in the figure legend for further clarification.

21) In my opinion iPSC reprogramming and differentiation potential of these cells should go into supplementary data, since it does not add much to the core message of the paper.

Agreed. We updated the manuscript to reflect the changes.

© 2022. Published by The Company of Biologists under the terms of the Creative Commons Attribution License (https://creativecommons.org/licenses/by/4.0/).

22) p.value in graphs. Either give real numbers not > or <, or categorize by *, **, ***. Having actual numbers combined with < is consistent as < 0.0001 could also be put as ***.

This is a good point. We updated the manuscript to reflect the changes.

23) The intro of the discussion is unnecessarily long and unspecific, taking away space for more specific aspects which in the current manuscript are not discussed.

We updated the manuscript to reflect the changes.

References

Albert, M., Kalebic, N., Florio, M., Lakshmanaperumal, N., Haffner, C., Brandl, H., Henry, I. and Huttner, W. B. (2017). Epigenome profiling and editing of neocortical progenitor cells during development. EMBO J. 36, 2642–2658.
Alves, C. A. P. F., Teixeira, S. R., Martin-Saavedra, J. S., Guimarães Gonçalves, F., Lo Russo, F., Muraresku, C., McCormick, E. M., Falk, M. J., Zolkipli-Cunningham, Z., Ganetzky, R., et al. (2020). Pediatric Leigh Syndrome: Neuroimaging Features and Genetic Correlations. Ann. Neurol. 88, 218-232.
Bershteyn, M., Nowakowski, T. J., Pollen, A. A., Di Lullo, E., Nene, A., Wynshaw-Boris, A. and Kriegstein, A. R. (2017). Human iPSC-Derived Cerebral Organoids Model Cellular Features of Lissencephaly and Reveal Prolonged Mitosis of Outer Radial Glia. Cell Stem Cell 20, 435-449.e4.
Camp, J. G., Badsha, F., Florio, M., Kanton, S., Gerber, T., Wilsch-Bräuninger, M., Lewitus, E., Sykes, A., Hevers, W., Lancaster, M., et al. (2015). Human cerebral organoids recapitulate gene expression programs of fetal neocortex development. Proc. Natl. Acad. Sci. 112, 201520760.
Florio, M., Namba, T., Paabo, S., Hiller, M. and Huttner, W. B. (2016). A single splice site mutation in human-specific ARHGAP11B causes basal progenitor amplification. Sci. Adv. 2,.
Inak, G., Rybak-Wolf, A., Lisowski, P., Pentimalli, T. M., Jüttner, R., Glažar, P., Uppal, K., Bottani, E., Brunetti, D., Secker, C., et al. (2021). Defective metabolic programming impairs early neuronal morphogenesis in neural cultures and an organoid model of Leigh syndrome. Nat. Commun. 12, 1–22.
Johnson, M. T., Yang, H. S., Magnuson, T. and Patel, M. S. (1997). Targeted disruption of the murine dihydrolipoamide dehydrogenase gene (Dld) results in perigastrulation lethality. Proc. Natl. Acad. Sci. U. S. A. 94, 14512–14517.
Johnson, M. T., Mahmood, S., Hyatt, S. L., Yang, H. S., Soloway, P. D., Hanson, R. W. and Patel, M. S. (2001). Inactivation of the murine pyruvate dehydrogenase (Pdha1) gene and its effect on early embryonic development. Mol. Genet. Metab. 74, 293–302.
Johnson, M. B., Wang, P. P., Atabay, K. D., Murphy, E. A., Doan, R. N., Hecht, J. L. and Walsh, C. A. (2015). Single-cell analysis reveals transcriptional heterogeneity of neural progenitors in human cortex. Nat. Neurosci. 18, 637–646.
Joshi, P., Bodnya, C., Rasmussen, M. L., Romero-Morales, A. I., Bright, A. and Gama, V. (2020). Modeling the function of BAX and BAK in early human brain development using iPSC-derived systems. Cell Death Dis. 11, 808.
Juchniewicz, P., Piotrowska, E., Kloska, A., Podlacha, M., Mantej, J., Węgrzyn, G., Tukaj, S. and Jakóbkiewicz-banecka, J. (2021). Dosage compensation in females with X-linked metabolic disorders. Int. J. Mol. Sci. 22, 4514.
Kuang, Y.-L., Munoz, A., Nalula, G., Santostefano, K. E., Sanghez, V., Sanchez, G., Terada, N., Mattis, A. N., Iacovino, M., Iribarren, C., et al. (2019). Evaluation of commonly used ectoderm markers in iPSC trilineage differentiation. Stem Cell Res. 37, 101434.
Lake, N. J., Compton, A. G., Rahman, S. and Thorburn, D. R. (2016). Leigh syndrome: One disorder, more than 75 monogenic causes. Ann. Neurol. 79, 190–203.
Lancaster, M. A., Corsini, N. S., Wolfinger, S., Gustafson, E. H., Phillips, A. W., Burkard, T. R., Otani, T., Livesey, F. J. and Knoblich, J. A. (2017). Guided self-organization and cortical plate formation in human brain organoids. Nat. Publ. Gr. 35,.
Lissens, W., De Meirleir, L., Seneca, S., Liebaers, I., Brown, G. K., Brown, R. M., Ito, M., Naito, E., Kuroda, Y., Kerr, D. S., et al. (2000). Mutations in the X-linked pyruvate dehydrogenase (E1) α subunit gene (PDHA1) in patients with a pyruvate dehydrogenase complex deficiency. Hum. Mutat. 15, 209–219.
Micali, N., Kim, S. K., Diaz-Bustamante, M., Stein-O'Brien, G., Seo, S., Shin, J. H., Rash, B. G., Ma, S., Wang, Y., Olivares, N. A., et al. (2020). Variation of Human Neural Stem Cells Generating

© 2022. Published by The Company of Biologists under the terms of the Creative Commons Attribution License (https://creativecommons.org/licenses/by/4.0/).

Organizer States In Vitro before Committing to Cortical Excitatory or Inhibitory Neuronal Fates. Cell Rep. 31,.
Migeon, B. R. (2020). X-linked diseases: susceptible females. Genet. Med. 22, 1156–1174.
Mok, B. Y., de Moraes, M. H., Zeng, J., Bosch, D. E., Kotrys, A. V., Raguram, A., Hsu, F., Radey, M. C., Peterson, S. B., Mootha, V. K., et al. (2020). A bacterial cytidine deaminase toxin enables CRISPR-free mitochondrial base editing. Nature 583, 631–637.
Ortolano, N. A., Romero-Morales, A. I., Rasmussen, M. L., Bodnya, C., Kline, L. A., Joshi, P., Connelly, J. P., Rose, K. L., Pruett-Miller, S. M. and Gama, V. (2021). A proteomics approach for the identification of cullin-9 (CUL9) related signaling pathways in induced pluripotent stem cell models. PLoS One 16,.
Pliss, L., Pentney, R. J., Johnson, M. T. and Patel, M. S. (2004). Biochemical and structural brain alterations in female mice with cerebral pyruvate dehydrogenase deficiency. J. Neurochem. 91, 1082–1091.
Pliss, L., Hausknecht, K. A., Stachowiak, M. K., Dlugos, C. A., Richards, J. B. and Patel, M. S. (2013). Cerebral Developmental Abnormalities in a Mouse with Systemic Pyruvate Dehydrogenase Deficiency. PLoS One 8, e67473.
Renner, M., Lancaster, M. A., Bian, S., Choi, H., Ku, T., Peer, A., Chung, K. and Knoblich, J. A. (2017). Self-organized developmental patterning and differentiation in cerebral organoids. EMBO J.
Roberts, B., Hendershott, M. C., Arakaki, J., Gerbin, K. A., Malik, H., Nelson, A., Gehring, J., Hookway, C., Ludmann, S. A., Yang, R., et al. (2019). Fluorescent Gene Tagging of Transcriptionally Silent Genes in hiPSCs. Stem Cell Reports 12, 1145–1158.
Sofou, K., De Coo, I. F. M., Ostergaard, E., Isohanni, P., Naess, K., De Meirleir, L., Tzoulis, C., Uusimaa, J., Lönnqvist, T., Bindoff, L. A., et al. (2018). Phenotype-genotype correlations in leigh syndrome: New insights from a multicentre study of 96 patients. J. Med. Genet. 55, 21–27.

<u>Second decision letter</u>

MS ID#: DEVELOP/2021/199914

MS TITLE: Human iPSC-derived cerebral organoids model features of Leigh Syndrome and reveal abnormal corticogenesis

AUTHORS: Alejandra I. Romero-Morales, Gabriella L. Robertson, Anuj Rastogi, Megan L. Rasmussen, Hoor Temuri, Gregory S. McElroy, Ram P. Chakrabarty, Lawrence Hsu, Paula M. Almonacid, Bryan A. Millis, Navdeep Chandel, Jean-Philippe Cartailler, and Vivian Gama

I apologise for the delay in making a decision on your manuscript. I'm afraid Dr Arlotta has had a family emergency and is unavailable for a few weeks. I have taken over as handling editor. I have now had the opportunity to read your paper and the referees comments. The referees' comments are appended below, or you can access them online: please go to BenchPress and click on the 'Manuscripts with Decisions' queue in the Author Area.

As you will see, the referees continue to express considerable interest in your work, but Reviewer 2 remains concerned about the regional identity of the organoids. Having read the manuscript myself, I agree with Reviewer 2. It seems to be essential to test the regional identity of the organoids derived from different IPS lines in order to support the conclusion that the mutations lead to abnormal corticogenesis. As Reviewer 2 suggests this could be achieved using a panel of Q-PCR primers for informative markers. I would encourage you to revise the manuscript along the lines suggested. I will be happy to receive a revised version of the manuscript. Your revised paper will be re-reviewed by one or more of the original referees, and acceptance of your manuscript will depend on your addressing satisfactorily the reviewers' major concerns.

We are aware that you may be experiencing disruption to the normal running of your lab that make experimental revisions challenging. If it would be helpful, we encourage you to contact us to discuss your revision in greater detail. Please send us a point-by-point response indicating where you are able to address concerns raised (either experimentally or by changes to the text) and

© 2022. Published by The Company of Biologists under the terms of the Creative Commons Attribution License (https://creativecommons.org/licenses/by/4.0/).

where you will not be able to do so within the normal timeframe of a revision. We will then provide further guidance. Please also note that we are happy to extend revision timeframes as necessary.

Please attend to all of the reviewers' comments and ensure that you clearly highlight all changes made in the revised manuscript. Please avoid using 'Tracked changes' in Word files as these are lost in PDF conversion. I should be grateful if you would also provide a point-by-point response detailing how you have dealt with the points raised by the reviewers in the 'Response to Reviewers' box. If you do not agree with any of their criticisms or suggestions please explain clearly why this is so.

Reviewer 1

Advance summary and potential significance to field

The authors have done a good job at addressing the reviewer concerns. The reviewer has no further comments and finds the manuscript suitable for publication.

Comments for the author

No comments

Reviewer 2

Advance summary and potential significance to field

Using the brain organoid model system the author Romero-Morales et al. delineate cellular and molecular changes that go along with mutations associate with Leigh Syndrom The impact of these mutation on early human brain development and the accompanying molecular, and there in particular metabolic, changes are of interest not only to neurobiologists but to developmental biologists in general.

Comments for the author

In the revised version of the manuscript entitled ‘Human iPSC-derived cerebral organoids model features of Leigh Syndrome and reveal abnormal corticogenesis’ the authors Romero-Morales et al. addressed a considerable fraction of the reservations I had towards the manuscript in the first submission. However the most crucial one was not addressed experimentally, i.e. the regional identity of the compared organoids. I appreciate the effort the authors put into generating isogenic lines and understand the pitfalls in doing this during the short time window of a revision. Hence, I also understand and agree with the reasoning the authors put forward in their response to the reviewers. On the other hand I do not agree and do not understand the reasoning why ‘qPCR analysis would be informative, we worry that the results could be misleading due to the heterogeneity of samples used in this study’. It is the fastest, cheapest and most doable set experiment to ascertain that the comparison made in this study, i.e. between organoids from independent lines with only one control line, are valid and that the conclusions that corticogeneis is abnormal is justified. After the dramatic difference seen in the Kanton et al. 2019 paper it is simply indispensable to clarify the regional identity of organoids from different IPS lines one is comparing to each other. To put it very blunt and provokativ, in mouse neurodevelopment no author would put forward the concept that the midbrain of Leigh Syndrom model mice has less Ctip2+ cells than the forebrain of controls.

Minor points:
Fig2B: in the figure the y-axis is labelled with ‘mean fluorescence intensity normalized to nuclear dapi’ in all plots. In the figure legend it says ‘positive nuclei number for nuclear markers, and mean fluorescence intensity for cytoplasmatic markers were normalized to the nuclear DAPI intensity/number and the intensity values of control’. I guess there is a mistake in the labelling of the y-axis in the PAX6 and OLIG2 plot.
Morever, in the figure the information which comparison is significantly different as is stated in the text

© 2022. Published by The Company of Biologists under the terms of the Creative Commons Attribution License (https://creativecommons.org/licenses/by/4.0/).

(line 166) is missing.
The cartoon in FigS6 suggests that PAX6+ and Nestin+ cells are 2 distinct populations of cells, while in fact PAX6+ cells are Nestin+, as also your stainings shown in FigS6C show.
MT-ATP6/PDH organoids show a lot of cyst like structures in Fig5B. increase in TBR2 might also reflect a difference in the likelihood of forming neural structures i.e. cyst like looking mesodermal structures

---

Second revision

Author response to reviewers' comments

We thank the Reviewers of Development for their time and feedback on our manuscript. Below you can find a detailed response to all reviewers' comments.

Reviewer 1
Advance summary and potential significance to field
The authors have done a good job at addressing the reviewer concerns. The reviewer has no further comments and finds the manuscript suitable for publication.

Comments for the author
No comments

We thank Reviewer 1 for their support in finding our manuscript suitable for publication in Development.

Reviewer 2
Advance summary and potential significance to field
Using the brain organoid model system the author Romero-Morales et al. delineate cellular and molecular changes that go along with mutations associate with Leigh Syndrome. The impact of these mutation on early human brain development and the accompanying molecular, and there in particular metabolic, changes are of interest not only to neurobiologists but to developmental biologists in general.

Reviewer 2 Comments for the author
In the revised version of the manuscript entitled ‘Human iPSC-derived cerebral organoids model features of Leigh Syndrome and reveal abnormal corticogenesis’ the authors Romero-Morales et al. addressed a considerable fraction of the reservations I had towards the manuscript in the first submission. However, the most crucial one was not addressed experimentally, i.e. the regional identity of the compared organoids. I appreciate the effort the authors put into generating isogenic lines and understand the pitfalls in doing this during the short time window of a revision. Hence, I also understand and agree with the reasoning the authors put forward in their response to the reviewers. On the other hand I do not agree and do not understand the reasoning why ‘qPCR analysis would be informative, we worry that the results could be misleading due to the heterogeneity of samples used in this study’. It is the fastest, cheapest and most doable set experiment to ascertain that the comparison made in this study, i.e. between organoids from independent lines with only one control line, are valid and that the conclusions that corticogenesis is abnormal is justified. After the dramatic difference seen in the Kanton et al. 2019 paper it is simply indispensable to clarify the regional identity of organoids from different IPS lines one is comparing to each other. To put it very blunt and provokativ, in mouse neurodevelopment no author would put forward the concept that the midbrain of Leigh Syndrome model mice has less Ctip2+ cells than the forebrain of controls.

We thank Reviewer 2 for their appreciation of the potential impact of our study but also for the critical comments raised, which have improved our interpretation of the data presented in this manuscript. We agree with Reviewer 2 that comparing the regional identity of the organoids is an important issue. Using the Kanton reference as a guide, and as recommended by Reviewer 2, we validated a few primers corresponding to markers of dorsal and ventral identity, as well as markers

© 2022. Published by The Company of Biologists under the terms of the Creative Commons Attribution License (https://creativecommons.org/licenses/by/4.0/).

of diencephalon and mesencephalon. In parallel to primer validation, we generated new batches of day 15 organoids and we have now included new data for reviewers, demonstrating that at this early developmental stage all samples have equivalent potential to give rise to the dorsal telencephalic identity (Figures 1 and 2 – for reviewers only). We think these new findings should be included in the new version of the paper. To complement these data, we also generated two new batches of day 30 organoids to test similar markers of regional identity with fetal brain as control, to complement the data already presented in Supp Figure 6. At this developmental stage, the double mutant organoids have lower expression of PAX6 and SOX2 genes compared to other genotypes (as we previously reported in the manuscript), but a similar expression to fetal brain. At the protein level, both PAX6 and SOX2 show similar expression in all the genotypes (Supplemental Figure 6F of the manuscript). Altogether, these data indicate that Leigh syndrome-associated mutations do not compromise the ability of cells at early stages to commit to a telencephalic fate. At later developmental times (day 30 organoids), the MT-ATP6/PDH double mutant has a statistically significant increase in the expression of GATA3 (Figure 3 to reviewers only) and of TBR2 (Supp Figure 6). GATA3, an early marker for mesencephalic fate, was significantly upregulated in the double mutant (p= 0.0005) when compared to the control. GATA3 is a key regulator gene of CNS development, and its expression is elevated during the early stages of development (Kizil et al., 2012; Nardelli et al., 1999; Zhao et al., 2008). As GATA3 is also expressed in non-neural ectoderm at the early stages of development (Ealy et al., 2016; Hackland et al., 2017; Neave et al., 1995; Sheng and Stern, 1999). Lastly, diencephalon markers GBX2 and PCP4 were also expressed at similar levels among all the genotypes. We agree with Reviewer 2 about the importance of discussing our data through the lens of the Kanton findings. While we believe our study represents an advance in the field, it is significantly limited by the small sample number, a common challenge in the rare diseases field. Thus, we have now discussed the urgent need of optimizing current approaches to streamline mitochondrial gene editing protocols, which would allow the engineering of several human iPSCs and ESCs lines with mitochondrial-related mutations commonly found in patients. These advancements are a needed next step in the field of rare mitochondrial diseases. We propose that the heterogeneity of the samples used in our study could prove advantageous when analyzed from a different perspective. Despite the heterogeneity of the cells used in this study, a point correctly raised by Reviewer 2, our research may have uncovered potentially common neurodevelopmental abnormalities shared across mitochondrial diseases caused by different mutations. It is becoming evident that organoid models may serve to find points of convergence in the neurobiological basis of how mitochondrial-related mutations contribute to the pathology of mitochondrial rare diseases.

Minor points:
1. Fig2B: in the figure the y-axis is labelled with 'mean fluorescence intensity normalized to nuclear dapi' in all plots. In the figure legend it says 'positive nuclei number for nuclear markers, and mean fluorescence intensity for cytoplasmatic markers were normalized to the nuclear DAPI intensity/number and the intensity values of control'. I guess there is a mistake in the labelling of the y-axis in the PAX6 and OLIG2 plot.

We thank Reviewer 2 for pointing out this error. The labels were corrected in the new version.

2. Moreover, in the figure the information which comparison is significantly different as is stated in the text (line 166) is missing.

We apologize for this oversight. We provided this information in the new version.

3. The cartoon in FigS6 suggests that PAX6+ and Nestin+ cells are 2 distinct populations of cells, while in fact PAX6+ cells are Nestin+, as also your stainings shown in FigS6C show.

The figure was corrected accordingly.

4. MT-ATP6/PDH organoids show a lot of cyst like structures in Fig5B. increase in TBR2 might also reflect a difference in the likelihood of forming neural structures i.e. cyst like looking mesodermal structures
From Lancaster 2021: Interestingly, there was a correlation between the abundance of non-neural mesodermal and endodermal cells and the quality and reliability of resultant organoids. Thus, we sought to improve the reliable generation of neuroectoderm at an early stage. Well-organized

© 2022. Published by The Company of Biologists under the terms of the Creative Commons Attribution License (https://creativecommons.org/licenses/by/4.0/).

neuroectoderm tends to form initially around the surface of the early embryoid body. In order to increase the relative abundance of this surface tissue, we previously experimented with different approaches including simply starting with fewer cells to generate smaller spheres, which led to more reliable formation of neural identities, and interestingly generated organoids with primarily a telecephalic identity.

We thank reviewer 2 for bringing up this point. Before beginning our project, we consulted with Dr. Lancaster about the best way to improve the reliable generation of organoids with a primarily telencephalic identity. The protocol used in our study was adapted after stringent optimization of the starting cell number as kindly recommended by Dr. Lancaster previous to their publication of 2021.

Third decision letter

MS ID#: DEVELOP/2021/199914

MS TITLE: Human iPSC-derived cerebral organoids model features of Leigh Syndrome and reveal abnormal corticogenesis

AUTHORS: Alejandra I. Romero-Morales, Gabriella L. Robertson, Anuj Rastogi, Megan L. Rasmussen, Hoor Temuri, Gregory S. McElroy, Ram P. Chakrabarty, Lawrence Hsu, Paula M. Almonacid, Bryan A. Millis, Navdeep Chandel, Jean-Philippe Cartailler, and Vivian Gama
ARTICLE TYPE: Techniques and Resources Article

I am happy to tell you that your manuscript has been accepted for publication in Development, pending our standard ethics checks.

Reviewer 2

Advance summary and potential significance to field

the authors addressed my concerns. I would have included the results of the qPCRs in the supplemental figures, given that these infos are valuable for the interpretation of the data.

However, I find the manuscript in its current version suitable for publication in development.

Comments for the author

no comments

© 2022. Published by The Company of Biologists under the terms of the Creative Commons Attribution License (https://creativecommons.org/licenses/by/4.0/).
